# Multi-level immune response network in mild-moderate Chronic Obstructive Pulmonary Disease (COPD)

**DOI:** 10.1186/s12931-019-1105-z

**Published:** 2019-07-12

**Authors:** Tamara Cruz, Alejandra López-Giraldo, Guillaume Noell, Sandra Casas-Recasens, Tamara Garcia, Laureano Molins, Manel Juan, Marco A. Fernandez, Alvar Agustí, Rosa Faner

**Affiliations:** 1 CIBER Enfermedades Respiratorias, Barcelona, Spain; 2 Institut de Recerca Biomedica August Pi i Sunyer (IDIBAPS), Barcelona, Spain; 3 Respiratory Institute, Hospital Clinic, University of Barcelona, Barcelona, Spain; 4 Immunology Service, Centre Diagnostic Biomèdic, Hospital Clinic, University of Barcelona, Barcelona, Spain; 5 Flow Cytometry Facility, Institut de Recerca Germans Trias I Pujol, Barcelona, Spain; 6 CIBERES, IDIBAPS-CELLEX. Facultat de Medicina P2A, c/Casanova 143, 08036 Barcelona, Spain

**Keywords:** Chronic bronchitis, Emphysema, Flow cytometry, Transcriptome, Network analysis

## Abstract

**Background:**

Chronic Obstructive Pulmonary Disease (COPD) is associated with an abnormal pulmonary and systemic immune response to tobacco smoking. Yet, how do immune cells relate within and between these two biological compartments, how the pulmonary infiltrate influences the lung transcriptome, and what is the role of active smoking vs. presence of disease is unclear.

**Methods:**

To investigate these questions, we simultaneously collected lung tissue and blood from 65 individuals stratified by smoking habit and presence of the disease. The immune cell composition of both tissues was assessed by flow cytometry, whole lung transcriptome was determined with Affymetrix arrays, and we used Weighted Gene Co-expression Network Analysis (WGCNA) to integrate results.

**Results:**

Main results showed that: *(1)* current smoking and the presence of COPD were both independently associated with a reduction in the proportion of lung T cells and an increase of macrophages, specifically those expressing CD80 + CD163+; *(2)* changes in the proportion of infiltrating macrophages, smoking status or the level of airflow limitation were associated to different WGCNA modules, which were enriched in iron ion transport, extracellular matrix and cilium organization gene ontologies; and, *(3)* circulating white blood cells counts were correlated with lung macrophages and T cells.

**Conclusions:**

Mild-moderated COPD lung immune infiltrate is associated with the active smoking status and presence of disease; is associated with changes in whole lung tissue transcriptome and marginally reflected in blood.

**Electronic supplementary material:**

The online version of this article (10.1186/s12931-019-1105-z) contains supplementary material, which is available to authorized users.

## Background

Tobacco smoking is the main environmental risk factor for COPD, albeit not all smokers develop the disease [1, 2]. It is currently accepted that, in so-called “susceptible smokers”, tobacco smoking triggers an abnormal pulmonary and systemic immune response that eventually damages the lung parenchyma and leads to persistent airflow limitation and COPD [1–5]. Yet, some aspects of this pathogenic paradigm are still unclear and require specific research. For instance, although in cross-sectional studies [6–9] the number of inflammatory cells infiltrating the lung parenchyma increase in parallel with the severity of airflow limitation, how do these cells relate among themselves is unknown. Likewise, it is unclear how changes in the pulmonary immune cell network relate to changes in the whole lung transcriptome [10–13]. Besides, given that smoking per se has well recognized effects on the immune system [4, 14], it is important (and challenging) to disentangle the role of disease itself vs. that of active smoking. Finally, previous studies have tried to identify blood surrogate markers of lung changes [15, 16], but the relation between different immune cell types in the two compartments (i.e. lung and blood) has not been ever assessed.

We hypothesized that a data set that combined lung tissue and circulating blood measurements collected simultaneously in current and former smokers with COPD, as well as controls with normal lung function (current and never smokers), would be a unique asset to: *(1)* characterize the lung and blood immune cell composition in relation to both smoking status and presence of disease; *(2)* explore the relationship between the lung immune cell composition and the lung tissue transcriptome; and, finally, *(3)* construct a multi-level (lung and blood) immune cell correlation network to assess if the lung changes are reflected in the blood.

## Methods

Full methods are provided in the Additional file 1.

### Population and ethics

We prospectively collected clinically relevant information as well as lung tissue and blood samples from 65 patients who required thoracic surgery for lung cancer (Table 1). The lung tissue was from a non-affected location. All participants signed their informed consent, and the Ethics Committee of our institution approved the study (HCB-2012/7731).

**Table 1:** Clinical variables of the subjects enrolled in the analysis of the cellular immune response

	Non-Smokers	Smokers	COPD-FS	COPD-CS	*P* value
	*n* = 12	*n* = 9	*n* = 16	*n* = 28	
Age (years)	67.6 ± 9.5	58.0 ± 9.3	64.5 ± 8.1	67.8 ± 7.8	0.0587
Gender (M/F)	3/9	5/4	14/2	20/8	0.0051
Pack/year	–	36.8 ± 19.1	48.7 ± 19.9	51.1 ± 22.1	0.1830
BMI (Kg/m^2^)	28.0 ± 6.7	27.4 ± 5.4	24.9 ± 3.8	28.0 ± 3.5	0.0974
FEV_1_/FVC (%)	77.9 ± 4.1	79.1 ± 10.1	61.7 ± 6.5	59.9 ± 7.1	0.0000
FEV_1_ (% ref)	97.3 ± 8.1	95.1 ± 7.5	77.1 ± 13.0	77.3 ± 18.3	0.0001
DLCO (% ref.)	78.3 ± 16.2	78.9 ± 11.4	61.6 ± 9.6	71.4 ± 14.7	0.0021
WBC 10^9/L	7.5 ± 2.1	9.3 ± 4.01	6.5 ± 2.2	8.6 ± 2. 2	0.0193
Abs Neutrophil count (10^9/L)	5.0 ± 2.1	6.1 ± 4.0	4.0 ± 1.6	5.5 ± 1.8	0.0448
hsCRP mg/dl	0.72 ± 0.96	8.56 ± 18.09	0.62 ± 0.98	2.43 ± 4.94	0.0954

*FS* Former smokers**,** *CS* Current smokers**,** *WBC* white blood cell count**,** *hsCRP* high sensitivity C Reactive Protein**,** *BMI* Body Mass Index**,** *FEV*_*1*_ Forced expiratory volume 1 s**,** *FVC* forced vital capacity

*P* value of the kruskall wallis or chi square test

### Lung tissue processing

Fresh lung tissue was washed with PBS, enzymatically digested with 0.5 mg/ml Collagenase P and 0.1 mg/ml DNase I (Roche, Germany), incubated at 37 °C for 30 min, and mechanically digested with GentleMACS M-dissociator tubes (Miltenyi Biotech, Germany).

### Measurements

#### Lung function

Forced spirometry and carbon monoxide lung capacity (DLCO) were determined according to international recommendations [2] and reference values corresponded to a Mediterranean population [17].

#### Circulating inflammatory markers

Circulating venous blood was collected in EDTA tubes (BD, US) in the operating room before surgery started. Total white blood cells counts were quantified with an ADVIA-2120 system (Siemens, Germany). The plasma concentration of high sensitivity C Reactive Protein (hsCRP) was determined by ultra-sensitive quantitative turbidimetric test (Bayer Diagnostics, Germany).

#### Flow cytometry

Immune cell populations were analysed in parallel in blood and lung tissue. Lung tissue homogenates (5·10^5^ cells) or 50 μl of peripheral blood were stained with the corresponding fluorescently conjugate monoclonal antibody mix (Additional file 1: Table S1), incubated at 4 °C for 30 min and washed. A minimum of 2·10^5^ cells per tube were acquired in a FacsCanto (BD Biosciences, US), data was analyzed using FlowJo v10 software (FlowJo LLC, US) Additional file 2: Figures S1-S6.

#### Transcriptomic analysis

Total RNA was isolated with PureLink RNA-MiniKit (Life Technologies, US), quantified by Nanodrop (Thermo Scientific, Germany). RNA samples with integrity numbers (RIN) ≥ 7 (Agilent technologies, Germany), where analysed with the Affymetrix GeneChip® Human Genome U219 Array Plate. Only from 53 individuals the mRNA quality was adequate for analysis (Additional file 1: Table S2). Raw microarray data has been deposited in GEO (GSE103174).

### Data analysis

Statistical analysis were performed using R [18]. Results are presented as n, proportion, or mean ± standard deviation. Differences between groups were assessed using the Kruskal-Wallis test, followed by *post-hoc* contrasts if necessary (Table 2). A linear model after log transformation of variables was used to ascertain the independent contribution of smoking status and presence of disease. *P* values < 0.05 were considered significant. Lung-blood immune cell correlation networks were built with R and visualized using Cytoscape as described elsewhere (Spearman coefficient r > |0.3| and a *p*-value < 0.05) [19].

**Table 2:** Post-hoc analysis on lung cell populations reported as % of CD45 gated cells

Cell population	Group 1	Group 2	*P* value (Mann-Whitney)
% Mϕ	CS-COPD	FS-COPD	*0.004*
% Mϕ	CS-COPD	NS	*0.005*
% Monocytes	CS-COPD	FS-COPD	*0.015*
% Monocytes	CS-COPD	NS	*0.009*
% T cells	CS-COPD	FS-COPD	*0.002*
% T cells	CS-COPD	NS	*0.006*
% T cells	CS-COPD	S	*0.024*
% T CD4+	CS-COPD	FS-COPD	*0.008*
% T CD8+	CS-COPD	S	*0.035*
% T CD8+	CS-COPD	NS	*0.046*
% Mϕ CD80+ CD163-	CS-COPD	FS-COPD	*0.055*
% Mϕ CD80+ CD163-	CS-COPD	NS	*0.040*
% Mϕ CD80+ CD163-	S	NS	*0.036*
% Mϕ CD80+ CD163-	FS-COPD	S	*0.019*
% Mϕ CD80 + CD163+	CS-COPD	FS-COPD	*0.000*
% Mϕ CD80 + CD163+	CS-COPD	NS	*0.000*

**Fig. 1:**
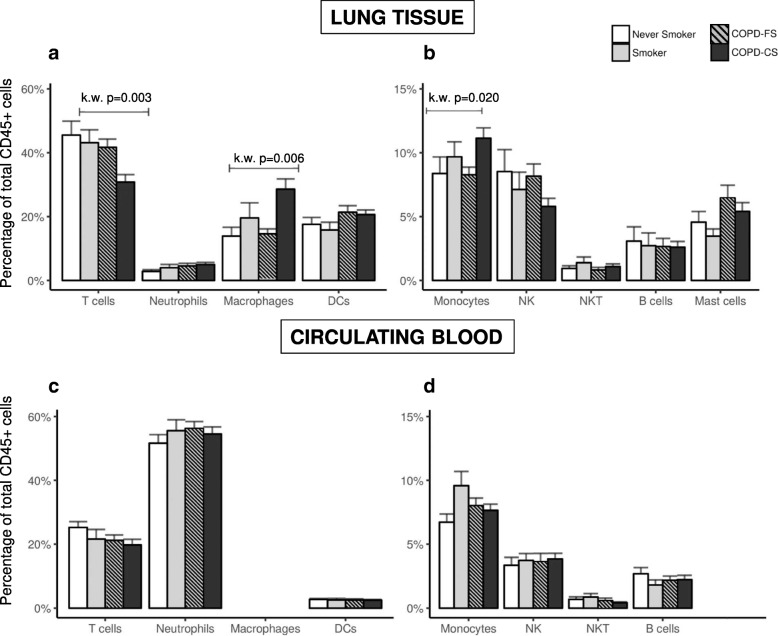
Proportion of immune cell populations (mean ± SD), expressed as percentage of total CD45+ cells, both in lung tissue (*Top panels* **a** and **b**) and circulating blood (*Bottom panels* **c** and **d**). Study groups: never smokers (white bars), smokers with normal lung function (grey bars), COPD-former smokers (COPD-FS, gray stripped bars) and COPD current smokers (COPD-CS, black bars). Note the different Y scales in right vs. left panels. For further explanations, see text

On populations that showed a significantly different distribution between the 4 study groups (Fig. 1 and Additional file 2: Figures S7-S8) a post-hoc analysis was performed using the Mann-Whitney on original values

*NS* non-smoker, *S* normal lung function smoker, *FS-COPD* Former smoker with COPD, *CS-COPD* Current Smoker with COPD

## Results

### Participant characteristics

Table 1 presents the main characteristics of participants. In patients with COPD the proportion of subjects with GOLD grade 1 airflow limitation (31% (*n* = 5) vs. 32% (*n* = 9)) and GOLD grade 2 (69% (*n* = 11) vs. 68% (*n* = 19)) was similar in former (FS) and current smokers (CS) patients, respectively. DLCO was mildly reduced in COPD patients. Circulating leukocyte and neutrophil counts and hsCRP plasma levels were increased in current smokers (with or without COPD) (Table 1).

### Characterization of lung and blood immune cells

Figure 1 (panels a and b) compares the proportion of the different lung immune cell populations studied, expressed as percentage of total CD45+ cells (hematopoietic lineage, a common practice in order to perform immunophenotyping in non-lymphoid tissues [20]) in the four groups investigated. Main differences were observed in current smokers with COPD (COPD-CS) who showed: *(1)* a significant reduction in the proportion of T-cells that involved both CD4+ and CD8+ T cells (Table 2 and Additional file 2: Figure S7); *(2)* a significant increase in the proportion of macrophages (Mϕ) (Fig. 1a), of whom a higher percentage expressed both CD80 + CD163+ (Table 2, Additional file 2: Figure S8) and a relative reduction of CD80 + CD163-Mϕ (Table 2, Additional file 2: Figure S8); and, *(3)* a different distribution of lung monocytes between study groups (Fig. 1b) due to an increase of monocytes in COPD-CS (Table 2, Additional file 2: Figure S8D).

As main changes were observed in COPD-CS, next we used a linear model in order to evaluate if both current smoking and presence of COPD have an independent effect. We observed that current smoking was inversely related to the proportion of lung T-lymphocytes (βeta = − 0.111, *p* value =0.008) and positively with that of macrophages (Mϕ) (βeta =0.210, *p* value =0.001) and monocytes (βeta =0.104, *p* value =0.005); while the presence of COPD had an independent but smaller effect (Mϕ, βeta =0.152, *p* value =0.05; T-lymphocytes, βeta = − 0.100, *p* value =0.065).

Finally, the proportion of the different immune cell populations in circulating blood was not different across groups (Fig. 1c and d).

### Relationship between lung immune cell population and whole lung transcriptome

To assess the association between the immune composition of the lung and the biological processes on-going in the lung, we measured the gene expression profile of the whole lung from 53 participants (82% of the total population studied, Additional file 1: Table S2) and used Weighted Gene Co-expression Network Analysis (WGCNA) to integrate the information. WGCNA defines a set of modules of co-expressed genes, and for each provides their “eigengene” value (i.e., the principal component of the expression matrix of the probes within the module). The eigengene can be related to the variables of interest [21], in our case FEV_1_, the smoking status and the percentage of lung macrophages (the cell population reporting more differences across study groups).

**Fig. 2:**
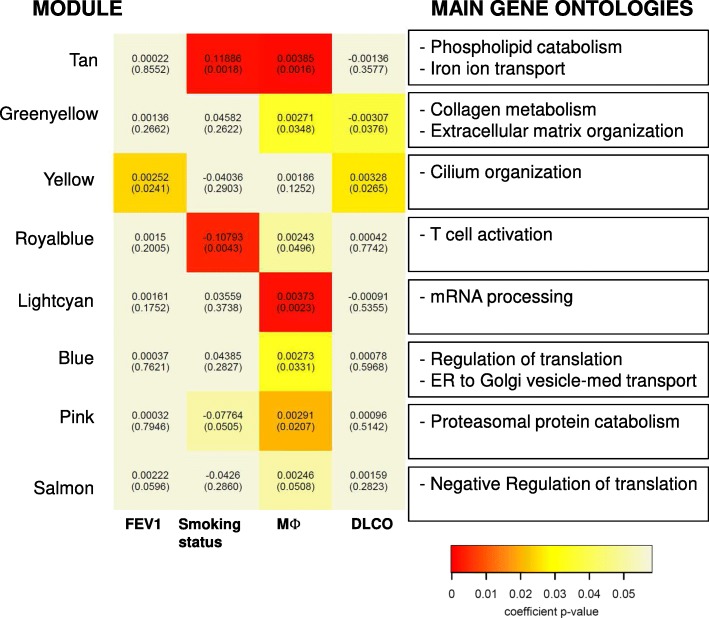
Heat-map of module association with: the level of FEV_1_% predicted, the smoking status, and the percentage of Macrophages (Mϕ)). The top number in each cell corresponds to the effect from the linear regression and the bottom number is the p-value. In the box, the summarizing terms for the Gene Ontology (GO) terms significantly enriched for this module obtained from the Revigo analysis are displayed. The full list of GO terms if provided in Additional file 1: Table S5. For further explanations, see text

WGCNA identified 23 modules (Additional file 1: Table S3), of them in eight the eigengene was significantly associated with the variables of interest (Fig. 2).

**Fig. 3:**
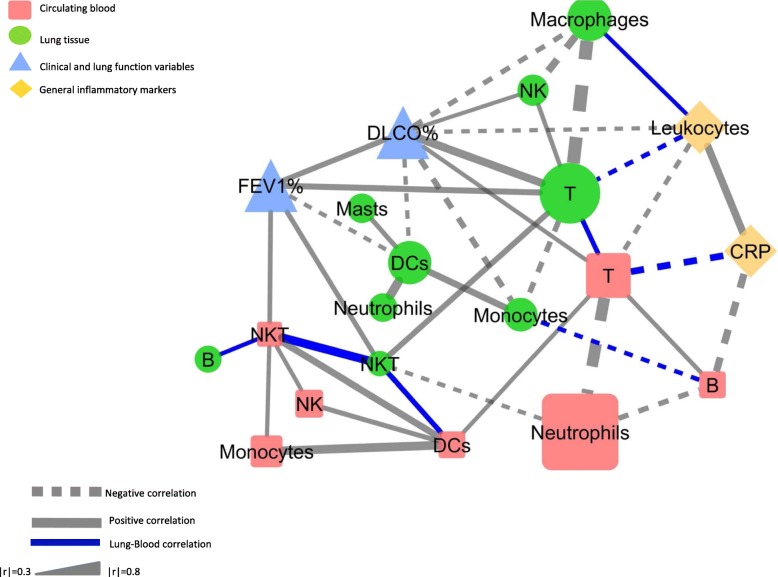
Multilevel (lung and blood) Spearman correlation network (|r| > 0.3, *p* < 0.05) for all participants (*n* = 65) of lung (green), peripheral blood (red) immune cell populations, lung function variables (blue) and general systemic inflamatory biomarkers (yellow). Positive correlations are represented with a continuous edge while the negatives are with a discontinuous edge. Edge witdth is proportional to the strength of the correlation. Node size is proportional to the mean percentatge of CD45% cells of the population. Blue links are used to highlight correlations between lung and blood markers. For further explanations, see text

Specifically, we observed that one module (*Yellow, n = 645* genes) was associated to the levels of FEV_1_ and DLCO % predicted. (Fig. 3) and was enriched in genes associated to the Cilium Organization Gene Ontology biological process term (GO) (Additional file 1: Tables S4-S5).

Three modules were associated both to the smoking status and percentage of infiltrating Mϕ (Fig. 3): *(i) Tan* (*n* = 211), enriched for iron metabolism and phospholipid metabolism GO terms (Additional file 1: Tables S4-S5), *(iii)* Royalblue (*n* = 86), related to T cell activation GO terms (Additional file 1: Tables S4-S5), *(iii) Lightcyan* (*n* = 140), related to mRNA processing GO terms (Additional file 1: Tables S4-S5).

Three modules were associated to the level of lung Mϕ expressing different surface markers (Fig. 2 and Additional file 2: Figure S9): *i) Greenyellow* (*n* = 227), especially related to CD80 + CD163+ Mϕ, with genes enriched in extracellular matrix organization and collagen metabolism GO terms (Additional file 1: Tables S4-S5). Interestingly this module also presented a negative correlation with the levels of DLCO % ref. *(ii) Blue (n =* 789), related to CD80-CD163+ Mϕ, with genes enriched in ER to Golgi vesicle transport and regulation of translation GO terms (S4-S5). *(iii) LightCyan (n =* 140), related to CD80-CD163+ Mϕ, with genes enriched in mRNA processing (S4-S5).

Finally, we assessed which of the module genes have been previously identified as differentially expressed in lung tissue of COPD (GSE47460). The 10 genes with highest differential expression (DE) included are reported in Table 3. Interestingly, the modules including more differentially expressed genes were the GreenYellow (*n* = 50) and the Yellow (*n* = 25) (Additional file 1: Table S6).

**Table 3:** Top Genes differentially expressed in Controls vs. COPD in the LTRC consortium dataset

	Module	symbol	adj.P.Val	logFC		Module	symbol	adj.P.Val	logFC
1	Yellow	FGG	5.54E-06	−1.92	1	Yellow	KRT4	2.10E-06	0.93
2	Greenyellow	PLA2G2A	2.21E-03	−1.03	2	Yellow	SLITRK6	1.76E-04	0.76
3	Blue	BNC1	9.91E-04	−0.99	3	Yellow	CDHR3	2.57E-02	0.70
4	Greenyellow	POU2AF1	6.61E-05	−0.93	4	Yellow	CFAP74	3.68E-02	0.60
5	Greenyellow	MZB1	1.96E-04	−0.84	5	Yellow	TNNI3	1.86E-03	0.56
6	Greenyellow	TNFRSF17	7.95E-04	−0.76	6	Yellow	SEC14L4	7.42E-05	0.55
7	Greenyellow	IGDCC4	4.07E-05	−0.68	7	Blue	PRPH	9.61E-06	0.54
8	Greenyellow	CPXM1	3.28E-03	−0.68	8	Yellow	SEC14L3	4.30E-02	0.53
9	Greenyellow	COL14A1	5.88E-05	−0.64	9	Yellow	PRMT8	1.07E-02	0.51
10	Greenyellow	CTHRC1	1.50E-03	−0.61	10	Yellow	VWA3A	3.99E-02	0.46

### Multi-level (blood and lung) immune cell correlation network

Finally, we build a multi-level correlation network [22] to investigate the potential relationships between immune cells and clinical variables in both lung and blood (Fig. 3) and observed that*: (1)* the strongest correlations were found between cells of the same tissue (percentage of lung Mϕ and T cells (r = − 0.81) and percentage of blood T cells and neutrophils (r = − 0.73); *(2)* eight of the 40 total connections (20%) involved lung and blood populations (Fig. 2, highlighted with blue edges). Of note, that the percentage of lung T cells was related to the percentage of blood T cells (r = 0.34), and the absolute counts of leukocytes (r = − 0.33). Interestingly, the absolute counts of blood leukocytes was also positively correlated with the proportion of lung Mϕ (r = 0.3); *(3)* FEV_1_% predicted was positively correlated with the proportion of lung T cells as well as lung and blood NKT cells, whereas it was negatively correlated with the proportion of lung DCs; and, finally *(4)* Carbon monoxide lung diffusing capacity (DLCO, % ref.), a well-validated surrogate marker of emphysema [23], was negatively correlated with the proportion of lung Mϕ, DCs and Monocytes, and positively correlated with those of lung T cells and peripheral blood T cells. It is of note that clinical variables were mostly correlated with lung tissue (not blood) populations (Fig. 3).

## Discussion

The main and novel observations of this study are that: *(1)* the composition of the immune cell infiltrate in the lung (but not in circulating blood) is significantly altered by active smoking and the presence of mild-moderate COPD. These changes are characterized by a reduction in the proportion of lung CD4+ and CD8+ T lymphocytes, and an increase in that of Mϕ, especially those expressing CD80 and CD163; *(2)* the percentage of lung tissue Mϕ is associated, independently of the airflow limitation and smoking status, to several modules of co-expressed genes in whole lung tissue including genes that have been previously associated with the pathobiology of COPD; and, finally *(3)* the immune cell multi-level correlation network shows some weak relations between lung and blood cells, the strongest one between the absolute number of blood leukocytes and the proportion of lung macrophages and T cells.

Many previous studies have investigated the pulmonary and systemic inflammatory response in COPD [6–8]. It is generally accepted that the absolute number of T-cells, both CD8+ cytotoxic T lymphocytes [24] and CD4+ T lymphocytes (polarized towards a TH1 response) [25], increase in patients with severe-very severe COPD. Our results in lung tissue show a reduction in the proportion of infiltrating T cells which was mostly related to smoking and only marginally to the presence of COPD. Apparently, therefore, our results do not agree with previous reports. However, to interpret them appropriately, it is important to consider that: *(1)* because our methodology does not allow the quantification of the absolute number of infiltrating cells, our results refer to their relative proportion (not to their absolute numbers); *(2)* we studied patients with mild-moderate, not severe-very severe, COPD. In fact, a recent study in bronchoalveolar lavage fluid (BALF) obtained from patients with mild-moderate COPD (GOLD grades 1–2) also reported a decrease in the proportion of T CD4+ cells and a clear effect of active smoking on cell population distribution [26]. This is indeed in line with our results and highlighting that GOLD grade and current smoking have both to be carefully considered when investigating the inflammatory response of COPD. On the other hand, it is well established that Mϕ are increased in BALF and lung tissue of COPD patients [27]. Our results are in keeping with these previous observations and extend them by showing that this increase is related independently both to active smoking and, to a lesser extent, to the presence of the disease.

Several previous studies have investigated lung transcriptomics in COPD [28, 29], but to our knowledge none has previously related the transcriptomic status with the proportion of infiltrating macrophages. Using WGCNA we identified four transcriptomic co-expression modules that were associated with the proportion of lung Mϕ independently of the level of airflow limitation and smoking status. Interestingly one module (*GreenYellow*) was associated with the percentage of CD80 + CD163+ macrophages and enriched in ontologies related to extracellular matrix organization and collagen metabolism. Interestingly this module presented a negative correlation with the levels of DLCO, in agreement with the findings of the multi-level correlation network. This module included genes differentially expressed in other lung tissue cohorts as: COL14A1, SFRP2 and CCDC80. Several reports have identified a differential expression of collagen genes in COPD [10], while SFRP2 has been related to a down-regulation of the WNT pathway and CCDC80 is expressed by alveolar macrophages [30]. Although this is a starting point for future research aimed at understanding mechanistically the pathogenic relevance of these associations, is in line with our previous reports of an abnormal catabasis in lung tissue macrophages of patients with COPD [31].

Finally, previous studies reported alterations in specific subsets of circulating blood cells, specifically an increase in total leukocytes and neutrophils in relation to current smoking has been described [5, 32]. But none used network analysis to relate lung and blood immune cells with clinical characteristics. Our results showed that circulating white cells (both total leukocyte counts) were negatively correlated with the percentage of lung T-cells and positively correlated with the percentage of lung Mϕ. Yet, the overall relation between the two compartments (lung and blood) is weak, so the blood immune cell composition cannot be used to predict the inflammatory status of the lung tissue.

The strengths of the study are that, for the first time it profiles a wide variety of immune cells, both in lung tissue and circulating blood, of a relatively large population of patients with mild moderated COPD and controls and uses multi-level network analysis to relate the immune response of the pulmonary and systemic compartments. Likewise, it is the first to investigate the lung transcriptome with the immune cell infiltrate. On the other hand, the limitations of the study are that all participants had surgically resectable lung cancer and that, although analysed lung samples were tumor-free, this can be a potentially confounding factor in our analysis. However, if this was the case, it might have affected similarly all samples analysed. Finally, it would have been interesting to assess how the immune infiltrate changes according to both current smoking and inhaled corticosteroid treatment (ICS) treatment, yet our cohort is underpowered to do these analysis as only COPD 8 patients were under ICS treatment.

## Conclusions

This study, by integrating high throughput analytical techniques in a unique, multi-level data set, provides novel information on the relation of the pulmonary and systemic inflammatory response that characterizes COPD. Our main findings highlight the role of lung macrophages and the active smoking status in the pathobiology of COPD. All in all, these observations contribute to a better understanding of COPD pathobiology.

## Additional files

Additional file 1: Online Supplement. (DOCX 576 kb)

Additional file 2: Supplementary Figures. (PDF 1969 kb)

## Data Availability

Data is available from GEO#(GSE103174) and from authors on a reasonable request.

## Abbreviations

BMI: Body mass index
COPD: Chronic obstructive pulmonary disease
DC: Dendritic cells
Mϕ: Macrophages
NKT: Natural killer T cells
WGCNA: Weighted Gene Co-expression Network Analysis
