# Supplementary material for: Multi-level immune response network in mild-moderate Chronic Obstructive Pulmonary Disease (COPD)
Source: Respir Res. 2019 Jul 12;20:152. doi: 10.1186/s12931-019-1105-z (PMC6626346; doi:10.1186/s12931-019-1105-z)
Supplement: Supplementary file 1 — Online Supplement. (DOCX 576 kb) [file 12931_2019_1105_MOESM1_ESM.docx]

Thursday, June 20, 2019

**On line supplement**

**MULTI-LEVEL Immune RESPONSE NETWORK IN PATIENTS WITH MILD-MODERATE CHRONIC OBSTRUCTIVE PULMONARY DISEASE (COPD)**

Tamara Cruz^1,2*^, Alejandra López-Giraldo^1-3*^, Guillaume Noel^1,2^, Sandra Casas-Recasens^1,2^, Tamara Garcia^1,2^, Laureano Molins ^1-3^, Manel Juan^4^, Marco A. Fernandez^5^, Alvar Agustí^1-3^, Rosa Faner^1,2^

**On-line methods and supplementary data:** 6 Tables, 9 Figures.

**Population and Ethics**

The diagnosis of COPD was established according to GOLD guidelines [1]. Exclusion criteria were treatment with systemic anti-inflammatory drugs, co-existence of other clinically significant inflammatory conditions and chemotherapy prior to surgery.

***Flow cytometry***

A haemolysis step was performed in the lung homogenates and the cellular suspension was filtered (serially with 100μm, 70 μm and 40 μm filters, BD, US). For blood samples, red blood cells were lysed (BD FACS^TM^ Lysing Solution, US).

In each tube we selected cells of hematopoietic lineage (CD45+) and then determined the frequency of Neutrophils, Macrophages (Mφ), Monocytes, Mast cells, Natural killer cells (NK cells), Natural killer T cells (NKT cells), Dendritic Cells (DCs), B cells and T cells. The gating strategy is detailed in the on-line supplement (Figures S1-S6). Three fluorescence minus one (FMO) controls were included to establish the gates of CD28 and Mφ markers. FMO tubes for Mφ allowed controlling for autofluorescence in the determination of CD80 and CD163 markers. In the used gating strategy the population of DCs may include NK cells that express CD11c and HLA-DR.

***Transcriptomics and WGCNA***

Microarray raw data was normalized with the robust multi-array average (RMA) method [2] and the probes in the lowest quartile of variability were removed. Then remaining probes were collapsed to genes using the collapseRows function **[3]** leading to a set of 16000 genes that were used to perform the WGCNA analysis**[3]**. In the WGCNA the adjacency matrix was built using the biweight midcorrelation (bicor), a softpower of 9, a DeepSplit for module identification of = 2 and a minimum module size n=30 genes. The module eigengene is defined as the first principal component of the expression matrix of the probes within the module. Each module eigengene was included in a linear regression model to identify modules associated with the level of FEV_1_ % predicted, the current or former smoking status or percentage of macrophages. Then the association with the percentage of macrophages (or its subtypes) adjusting as covariates for the level of airflow limitation (FEV_1_ % predicted) and the smoking status was computed [4]. The list of genes per module was used to compute the enrichment in gene ontology biological process using cluster profiler [5] and summarized in modules of interest using Revigo [6].

For the external validation of key genes, we computed the differentially expressed genes between COPD and controls on the LTRC dataset GEO# GSE47460; platform#GPL14550 with the GEO2R, considering significant a logFC>|0.3| and FDR p value < 0.05.

Table S1: Tubes and antibodies used for flow cytometry characterization of immune populations. The same staining was used in blood and lung tissue except for the macrophages (only in lung).

| **Tube, populations and volume** | **Dye** | **Brand and reference number** | **μl of antibody** |
| --- | --- | --- | --- |
| **#1: Neutrophils** | | | |
| CD45 | PECy7 | BD 560915 | 5 |
| CD16 | PE | BD 555407 | 5 |
| CD15 | APC | BD 551376 | 5 |
| CD66b | FITC | BD 555724 | 2.5 |
| **#2: T lymphocytes** | | | |
| CD45 | FITC | BD 555482 | 5 |
| CD3 | Alexa Fluor 750 | Beckman Coulter A66329 | 5 |
| CD4 | APC | BD 345771 | 5 |
| CD8 | PECy7 | BD 557750 | 2.5 |
| TcRγδ | PE | eBioscience 15-9986 | 2.5 |
| CD28 | PerCPCy5.5 | BD 560685 | 2.5 |
| **#3: B and NK lymphocytes** | | | |
| CD45 | FITC | BD 555482 | 5 |
| CD3 | Alexa Fluor 750 | Beckman Coulter A66329 | 5 |
| CD19 | PerCPCy5.5 | BD 561295 | 2.5 |
| CD16 | PE | BD 555407 | 5 |
| CD56 | PE | BD 555516 | 5 |
| **#4: NKT lymphocytes** | | | |
| CD45 | FITC | BD 555482 | 5 |
| CD3 | Alexa Fluor 750 | Beckman Coulter A66329 | 5 |
| CD56 | PE | BD 555516 | 5 |
| CD4 | APC | BD | 5 |
| CD8 | PECy7 | BD 557750 | 2.5 |
| **#5: Monocytes in blood** | | | |
| CD45 | FITC | BD 555482 | 5 |
| CD64 | PECy7 | BD 561191 | 2.5 |
| CD33 | Alexa Fluor 750 | Beckman Coulter A70200 | 5 |
| **# 6: Macrophages and Monocytes in lung** | | | |
| CD45 | FITC | BD 555482 | 5 |
| CD64 | PECy7 | BD 561191 | 2.5 |
| CD33 | Alexa Fluor 750 | Beckman Coulter A70200 | 5 |
| CD163 | Alexa Fluor 647 | BD 562669 | 2.5 |
| CD80 | PE | BD 557227 | 10 |
| **#7: Dendritic cells** | | | |
| CD45 | FITC | BD 555482 | 5 |
| CD33 | Alexa Fluor 750 | Beckman Coulter A70200 | 5 |
| CD11b | PECy7 | BD 557743 | 2.5 |
| CD11c | APC | BD 559877 | 5 |
| HLA-DR | PE | BD 555812 | 10 |
| **# 8: Mast Cells** |  |  |  |
| CD45 | FITC | BD 555482 | 5 |
| CD34 | PECy7 | BD 348811 | 5 |
| c-kit | PE | DAKO R 7145 | 10 |
| **# 9: FMO lymphocytes** | | | |
| CD45 | FITC | BD 555482 | 5 |
| CD3 | Alexa Fluor 750 | Beckman Coulter A66329 | 5 |
| CD4 | APC | BD 345771 | 5 |
| CD8 | PECy7 | BD 557750 | 2.5 |
| **# 10: FMO for CD80 in lung Macrophages** | | | |
| CD45 | FITC | BD 555482 | 5 |
| CD64 | PECy7 | BD 561191 | 2.5 |
| CD33 | Alexa Fluor 750 | Beckman Coulter A70200 | 5 |
| CD163 | Alexa Fluor 647 | BD 562669 | 2.5 |
| **# 11: FMO for CD163 in lung Macrophages** | | | |
| CD45 | FITC | BD 555482 | 5 |
| CD64 | PECy7 | BD 561191 | 2.5 |
| CD33 | Alexa Fluor 750 | Beckman Coulter A70200 | 5 |
| CD80 | PE | BD 557227 | 10 |

Table S2: Characteristics of subjects (n=53) analysed with WGCNA.

|  | **Non Smokers**  **n=10** | **Smokers**  **n=6** | **COPD-FSs**  **n=14** | **COPD-CS**  **n=23** |
| --- | --- | --- | --- | --- |
| Gender (M/F) | 1/9 | 3/3 | 12/2 | 15/8 |
| Age | 68.9 ± 9.9 | 61.3 ± 7.1 | 68.3 ± 7.7 | 64.9 ± 8.6 |
| Pack/year |  | 40 ± 21 | 50.3 ± 21.7 | 49.2 ± 21.2 |
| BMI | 28.8 ± 7.2 | 28.9 ± 6.2 | 27.9 ± 3.6 | 25.2 ± 4 |
| FEV1 (% ref) | 96.6 ± 8^****, ##^ | 95.8 ± 9 ^++, †^ | 77.2 ± 19.6 ^##, †^ | 75.7 ± 13.7 ^****, ++^ |
| FEV1/FVC (%) | 78.2 ± 4.4^****, ####^ | 82.8 ± 12.5 ^+, †^ | 59.5 ± 7.4^####, †^ | 61.5 ± 6.6^****, +^ |
| DLCO | 74.9 ± 13.2^*^ | 84.2 ± 8.5 ^+, †^ | 70.4 ± 15.5 ^†^ | 61.3 ± 9.4 ^*, +^ |

Differences between the four study groups were assessed with a Kruskal-Wallis test, followed by a Mann- Whitney post-hoc test if the Kruskal-Wallis p value was <0.05. In the table, post-hoc p values are provided. The different comparisons are denoted with the following symbols: (*) non-smokers vs. COPD current smokers (#) non-smokers vs. COPD former smokers (+)smokers vs. COPD current smokers and (†)smokers vs. COPD former smokers.p-values < 0.05 are indicated with one symbol, p-value < 0.005 with two symbols, p-value < 0.0005 with three symbols and p-value < 0.00005 with four symbols.

**Table S3:** Number of genes per WGCNA module and number of significantly enriched biological process gene ontologies (FRD <0.05).

| **Module Name** | **# Module genes** | **# Gene Ontology BP** |
| --- | --- | --- |
| Blue | 789 | 21 |
| Darkred | 62 | 0 |
| Green | 506 | 0 |
| Greenyellow | 227 | 240 |
| Lightcyan | 140 | 3 |
| Pink | 318 | 3 |
| Royalblue | 86 | 142 |
| Salmon | 199 | 7 |
| Tan | 211 | 96 |
| Yellow | 645 | 41 |
| Grey60 | 112 | 0 |
| Lightgreen | 105 | 0 |
| Darkgreen | 52 | 0 |
| Turquoise | 889 | 0 |
| Cyan | 160 | 0 |
| Red | 435 | 0 |
| Purple | 234 | 0 |
| Brown | 705 | 0 |
| Lightyellow | 87 | 0 |
| Black | 410 | 0 |
| Magenta | 262 | 0 |
| Darkturquoise | 43 | 0 |
| Midnightblue | 55 | 0 |

**Table S4.** Genes per module and Module Membership (MM) values for each.

| Module | Symbol | MM | Module | Symbol | MM | Module | Symbol | MM | Module | Symbol | MM | Module | Symbol | MM | Module | Symbol | MM | Module | Symbol | MM |
| --- | --- | --- | --- | --- | --- | --- | --- | --- | --- | --- | --- | --- | --- | --- | --- | --- | --- | --- | --- | --- |
| tan | ACP5 | 0.95 | greenyellow | PRRX1 | 0.93 | salmon | ZMAT1 | 0.92 | royalblue | IL23A | 0.93 | lightcyan | ACTB | 0.96 | blue | TM9SF2 | 0.95 | pink | DGKZ | 0.92 |
| tan | C1QA | 0.92 | greenyellow | CCDC80 | 0.92 | salmon | RSF1 | 0.92 | royalblue | TRAT1 | 0.91 | lightcyan | EIF4H | 0.95 | blue | CD46 | 0.93 | pink | GIGYF1 | 0.89 |
| tan | TREM2 | 0.91 | greenyellow | FBLN2 | 0.92 | salmon | TAOK2 | 0.87 | royalblue | PTPRCAP | 0.90 | lightcyan | RHOA | 0.94 | blue | C1GALT1C1 | 0.92 | pink | GNAI2 | 0.88 |
| tan | CAPG | 0.91 | greenyellow | PODN | 0.91 | salmon | SREK1IP1 | 0.85 | royalblue | CD3D | 0.90 | lightcyan | PGAM1 | 0.91 | blue | PRDX3 | 0.92 | pink | MAZ | 0.87 |
| tan | NCEH1 | 0.90 | greenyellow | TGFB3 | 0.91 | salmon | TMEM106B | 0.85 | royalblue | ACAP1 | 0.90 | lightcyan | CSNK2B | 0.90 | blue | ADAM10 | 0.92 | pink | ARPC4 | 0.87 |
| tan | FBP1 | 0.90 | greenyellow | MMP2 | 0.91 | salmon | ACVR2A | 0.85 | royalblue | CD2 | 0.90 | lightcyan | TPRG1L | 0.90 | blue | CAPZA1 | 0.92 | pink | RHOT2 | 0.86 |
| tan | GCHFR | 0.90 | greenyellow | COL15A1 | 0.90 | salmon | CWF19L2 | 0.84 | royalblue | LCK | 0.90 | lightcyan | GAPDH | 0.90 | blue | HNRNPK | 0.92 | pink | CLK2 | 0.86 |
| tan | DNASE2 | 0.90 | greenyellow | SERPINF1 | 0.90 | salmon | SMAD4 | 0.84 | royalblue | JAK3 | 0.89 | lightcyan | EIF3L | 0.90 | blue | SDHB | 0.91 | pink | KLHDC3 | 0.86 |
| tan | VSIG4 | 0.89 | greenyellow | FKBP10 | 0.90 | salmon | LUC7L3 | 0.82 | royalblue | INPP5D | 0.88 | lightcyan | UQCRC1 | 0.88 | blue | CNBP | 0.91 | pink | CTDNEP1 | 0.86 |
| tan | HEXB | 0.89 | greenyellow | FBN1 | 0.90 | salmon | TROVE2 | 0.81 | royalblue | CD48 | 0.88 | lightcyan | PHB2 | 0.88 | blue | DENND5A | 0.90 | pink | ARHGEF2 | 0.85 |
| tan | CTSD | 0.89 | greenyellow | COL14A1 | 0.90 | salmon | SRSF11 | 0.81 | royalblue | CD96 | 0.88 | lightcyan | ALKBH5 | 0.88 | blue | NCOA4 | 0.90 | pink | TOP3B | 0.84 |
| tan | RMDN3 | 0.89 | greenyellow | CXCL12 | 0.90 | salmon | LARP7 | 0.80 | royalblue | RASAL3 | 0.88 | lightcyan | WDR1 | 0.87 | blue | TMX1 | 0.90 | pink | ANKZF1 | 0.84 |
| tan | CTSZ | 0.88 | greenyellow | FBLN1 | 0.88 | salmon | PWAR6 | 0.80 | royalblue | LTB | 0.88 | lightcyan | CHMP7 | 0.87 | blue | GDI2 | 0.89 | pink | ZNF692 | 0.83 |
| tan | MARCO | 0.88 | greenyellow | COL1A2 | 0.88 | salmon | SMC3 | 0.80 | royalblue | AKNA | 0.87 | lightcyan | DAP | 0.86 | blue | ATP6AP2 | 0.89 | pink | MADD | 0.83 |
| tan | HEXA | 0.87 | greenyellow | COL3A1 | 0.88 | salmon | RCOR3 | 0.80 | royalblue | P2RY8 | 0.87 | lightcyan | SEC61A1 | 0.86 | blue | GYG1 | 0.89 | pink | CUL9 | 0.83 |
| tan | MS4A4A | 0.87 | greenyellow | SMOC2 | 0.87 | salmon | TTC17 | 0.80 | royalblue | FYB | 0.87 | lightcyan | NR1H2 | 0.85 | blue | ATP5C1 | 0.89 | pink | ACIN1 | 0.83 |
| tan | AKR1A1 | 0.86 | greenyellow | PDE2A | 0.87 | salmon | ZMYM4 | 0.80 | royalblue | ARHGAP9 | 0.87 | lightcyan | PTDSS1 | 0.85 | blue | RTN3 | 0.89 | pink | ACSS1 | 0.82 |
| tan | CD68 | 0.86 | greenyellow | HSPG2 | 0.87 | salmon | ZRANB2 | 0.79 | royalblue | CORO1A | 0.87 | lightcyan | EIF3H | 0.85 | blue | CAPZA2 | 0.88 | pink | ZNF384 | 0.82 |
| tan | HLA-DMA | 0.86 | greenyellow | CPXM2 | 0.87 | salmon | TNKS | 0.79 | royalblue | CD6 | 0.86 | lightcyan | SLC25A11 | 0.84 | blue | LEMD3 | 0.88 | pink | MAP2K7 | 0.82 |
| tan | CSTB | 0.85 | greenyellow | COL6A3 | 0.87 | salmon | CWC27 | 0.79 | royalblue | SEMA4D | 0.86 | lightcyan | EFCAB14 | 0.84 | blue | CMPK1 | 0.88 | pink | POLE | 0.82 |
| tan | ADTRP | 0.85 | greenyellow | OGN | 0.87 | salmon | ZNF260 | 0.79 | royalblue | CD5 | 0.86 | lightcyan | TRIM44 | 0.84 | blue | SRP9 | 0.88 | pink | CTDSP1 | 0.82 |
| tan | IDH1 | 0.85 | greenyellow | COL5A2 | 0.87 | salmon | THOC2 | 0.79 | royalblue | BCL11B | 0.86 | lightcyan | CFL1 | 0.84 | blue | MARCH7 | 0.88 | pink | INPPL1 | 0.82 |
| tan | HLA-DPA1 | 0.84 | greenyellow | HTRA1 | 0.87 | salmon | CREBZF | 0.78 | royalblue | SH2D1A | 0.86 | lightcyan | NAPA | 0.83 | blue | GOLPH3 | 0.88 | pink | LENG8 | 0.81 |
| tan | FTL | 0.84 | greenyellow | COL5A1 | 0.86 | salmon | RAB28 | 0.78 | royalblue | LIME1 | 0.86 | lightcyan | NONO | 0.83 | blue | CD164 | 0.88 | pink | CHMP1A | 0.81 |
| tan | CTSB | 0.83 | greenyellow | IGF1 | 0.86 | salmon | TBL1XR1 | 0.78 | royalblue | RUNX3 | 0.86 | lightcyan | EEF1G | 0.82 | blue | UTP6 | 0.88 | pink | GDI1 | 0.81 |
| tan | CCL18 | 0.83 | greenyellow | CD248 | 0.86 | salmon | TTC14 | 0.78 | royalblue | ARHGAP45 | 0.85 | lightcyan | EEF2 | 0.81 | blue | DYNC1LI1 | 0.88 | pink | RBM10 | 0.81 |
| tan | LGALS3 | 0.83 | greenyellow | LAMA2 | 0.86 | salmon | CASZ1 | 0.77 | royalblue | CCL5 | 0.85 | lightcyan | C10orf128 | 0.80 | blue | RAB14 | 0.88 | pink | YPEL3 | 0.81 |
| tan | HLA-DPB1 | 0.83 | greenyellow | MOXD1 | 0.86 | salmon | DDX6 | 0.77 | royalblue | IL2RG | 0.85 | lightcyan | CAB39 | 0.80 | blue | SEC23IP | 0.87 | pink | MAP2K2 | 0.81 |
| tan | FPR3 | 0.82 | greenyellow | THBS2 | 0.85 | salmon | CEP44 | 0.77 | royalblue | THEMIS | 0.85 | lightcyan | VAPB | 0.80 | blue | PRKAR1A | 0.87 | pink | KMT2A | 0.81 |
| tan | MDH1 | 0.81 | greenyellow | SH3PXD2A | 0.85 | salmon | CRBN | 0.76 | royalblue | SKAP1 | 0.84 | lightcyan | HK1 | 0.79 | blue | TMEM30A | 0.87 | pink | RXRB | 0.81 |
| tan | COL8A2 | 0.81 | greenyellow | COL16A1 | 0.85 | salmon | TNRC6A | 0.76 | royalblue | ICAM3 | 0.84 | lightcyan | CS | 0.79 | blue | PRPF4B | 0.87 | pink | POLRMT | 0.81 |
| tan | ACOT7 | 0.80 | greenyellow | BICC1 | 0.85 | salmon | KLHDC1 | 0.76 | royalblue | CARD11 | 0.84 | lightcyan | TFG | 0.79 | blue | UBXN4 | 0.87 | pink | CDK5RAP3 | 0.80 |
| tan | GPNMB | 0.80 | greenyellow | MGP | 0.85 | salmon | ZNF268 | 0.76 | royalblue | CLEC2D | 0.84 | lightcyan | TPD52L2 | 0.78 | blue | SDCBP | 0.87 | pink | LYPLA2 | 0.80 |
| tan | CREG1 | 0.80 | greenyellow | AEBP1 | 0.85 | salmon | NUCKS1 | 0.76 | royalblue | CCR7 | 0.84 | lightcyan | MTMR14 | 0.77 | blue | PPP1CC | 0.87 | pink | MKRN1 | 0.80 |
| tan | SLC38A6 | 0.80 | greenyellow | PLAT | 0.85 | salmon | RMDN1 | 0.75 | royalblue | SLA2 | 0.84 | lightcyan | TMEM109 | 0.77 | blue | ADIPOR1 | 0.87 | pink | HDAC5 | 0.80 |
| tan | ABHD12 | 0.79 | greenyellow | GLT8D2 | 0.84 | salmon | ARGLU1 | 0.75 | royalblue | WIPF1 | 0.84 | lightcyan | PABPC1 | 0.76 | blue | TMED2 | 0.86 | pink | SETD1B | 0.80 |
| tan | GALNT12 | 0.79 | greenyellow | SPON1 | 0.84 | salmon | UBE3B | 0.75 | royalblue | P2RY10 | 0.83 | lightcyan | CERS5 | 0.76 | blue | IL13RA1 | 0.86 | pink | FOXO4 | 0.80 |
| tan | CYP27A1 | 0.79 | greenyellow | ISLR | 0.84 | salmon | CHD9 | 0.75 | royalblue | TBC1D10C | 0.83 | lightcyan | PFKL | 0.74 | blue | RAB18 | 0.86 | pink | BORCS6 | 0.79 |
| tan | GPD1 | 0.79 | greenyellow | SRPX | 0.84 | salmon | GTPBP8 | 0.75 | royalblue | PSTPIP1 | 0.83 | lightcyan | SSU72 | 0.74 | blue | API5 | 0.86 | pink | SPSB3 | 0.79 |
| tan | PTPMT1 | 0.79 | greenyellow | ADGRA2 | 0.84 | salmon | EPM2AIP1 | 0.74 | royalblue | CST7 | 0.83 | lightcyan | HNRNPA1 | 0.74 | blue | PJA2 | 0.86 | pink | ZRSR2 | 0.79 |
| tan | LIPA | 0.79 | greenyellow | PDZRN3 | 0.84 | salmon | TOM1L2 | 0.74 | royalblue | CD3G | 0.83 | lightcyan | MYH9 | 0.74 | blue | TMBIM6 | 0.86 | pink | ZNF688 | 0.79 |
| tan | FUCA1 | 0.78 | greenyellow | SERPINE2 | 0.84 | salmon | PNISR | 0.74 | royalblue | IKZF1 | 0.82 | lightcyan | DMAP1 | 0.73 | blue | RAC1 | 0.86 | pink | DPP9 | 0.79 |
| tan | APOC1 | 0.78 | greenyellow | PCOLCE | 0.84 | salmon | CEP290 | 0.74 | royalblue | RASGRP1 | 0.82 | lightcyan | RNPS1 | 0.72 | blue | TPM3 | 0.86 | pink | C6orf136 | 0.78 |
| tan | CTSL | 0.77 | greenyellow | EMILIN1 | 0.84 | salmon | NPHP3 | 0.73 | royalblue | ADAM19 | 0.81 | lightcyan | NXF1 | 0.71 | blue | DAZAP2 | 0.86 | pink | MIB2 | 0.78 |
| tan | HLA-DMB | 0.77 | greenyellow | FGFR1 | 0.83 | salmon | MINDY2 | 0.73 | royalblue | ITK | 0.81 | lightcyan | SLC3A2 | 0.71 | blue | DERL1 | 0.86 | pink | RNPEPL1 | 0.78 |
| tan | CD163 | 0.77 | greenyellow | SEPT8 | 0.83 | salmon | PHF3 | 0.73 | royalblue | SIT1 | 0.81 | lightcyan | DNAJB12 | 0.71 | blue | NDUFS1 | 0.86 | pink | RBM5 | 0.78 |
| tan | APIP | 0.77 | greenyellow | COL6A1 | 0.82 | salmon | MTR | 0.72 | royalblue | ITGB7 | 0.81 | lightcyan | TGOLN2 | 0.71 | blue | PPP3R1 | 0.86 | pink | PPP1R9B | 0.78 |
| tan | AKR1B1 | 0.76 | greenyellow | MATN2 | 0.82 | salmon | ZNF322 | 0.72 | royalblue | ZAP70 | 0.81 | lightcyan | FLOT1 | 0.70 | blue | DPYD | 0.86 | pink | ATG13 | 0.78 |
| tan | FABP3 | 0.76 | greenyellow | KDELC2 | 0.82 | salmon | KTN1 | 0.72 | royalblue | PYHIN1 | 0.80 | lightcyan | PAF1 | 0.70 | blue | MBD2 | 0.86 | pink | SUGP2 | 0.77 |
| tan | STAC | 0.76 | greenyellow | SNED1 | 0.82 | salmon | SMARCAD1 | 0.72 | royalblue | SLAMF6 | 0.80 | lightcyan | RBM22 | 0.70 | blue | ZFAND6 | 0.86 | pink | PRR14 | 0.77 |
| tan | GPR137B | 0.76 | greenyellow | FSTL1 | 0.82 | salmon | THOC1 | 0.72 | royalblue | JAKMIP1 | 0.80 | lightcyan | C21orf33 | 0.68 | blue | SDHD | 0.86 | pink | OTUD5 | 0.77 |
| tan | MCOLN1 | 0.75 | greenyellow | RGMA | 0.81 | salmon | DCAF8 | 0.72 | royalblue | STAT4 | 0.80 | lightcyan | ABCF2 | 0.67 | blue | YME1L1 | 0.85 | pink | USP20 | 0.77 |
| tan | ATP6AP1 | 0.75 | greenyellow | SLIT3 | 0.81 | salmon | MSANTD2 | 0.72 | royalblue | CCR5 | 0.80 | lightcyan | DCAF12 | 0.67 | blue | VPS4B | 0.85 | pink | MAPK8IP3 | 0.77 |
| tan | FTH1 | 0.75 | greenyellow | PLTP | 0.81 | salmon | EIF5B | 0.72 | royalblue | GZMA | 0.79 | lightcyan | ST6GALNAC6 | 0.66 | blue | AZIN1 | 0.85 | pink | PPP1CA | 0.77 |
| tan | ALDH2 | 0.75 | greenyellow | IGFBP5 | 0.81 | salmon | BLCAP | 0.72 | royalblue | RBM38 | 0.79 | lightcyan | TINF2 | 0.66 | blue | PAIP2 | 0.85 | pink | FANCD2 | 0.77 |
| tan | COLGALT1 | 0.75 | greenyellow | ANGPTL2 | 0.81 | salmon | CTBP2 | 0.72 | royalblue | RHBDF2 | 0.78 | lightcyan | CYB5R3 | 0.65 | blue | SRSF10 | 0.85 | pink | EML3 | 0.77 |
| tan | HLA-DRB1 | 0.75 | greenyellow | MXRA8 | 0.81 | salmon | UBN2 | 0.72 | royalblue | NDC80 | 0.78 | lightcyan | MCM6 | 0.65 | blue | MBNL1 | 0.85 | pink | ATXN7L1 | 0.77 |
| tan | BCAT1 | 0.74 | greenyellow | APOD | 0.81 | salmon | USP33 | 0.71 | royalblue | NLRC5 | 0.77 | lightcyan | UNC45A | 0.65 | blue | VBP1 | 0.85 | pink | MCM3AP | 0.76 |
| tan | SIGLEC1 | 0.74 | greenyellow | C1S | 0.81 | salmon | FAM76B | 0.71 | royalblue | NLRC3 | 0.77 | lightcyan | RNF220 | 0.63 | blue | M6PR | 0.85 | pink | RNPC3 | 0.76 |
| tan | PLA2G15 | 0.74 | greenyellow | OLFM1 | 0.80 | salmon | EIF2D | 0.71 | royalblue | ITGAL | 0.77 | lightcyan | PLOD1 | 0.63 | blue | NPTN | 0.85 | pink | RNF40 | 0.76 |
| tan | BLVRA | 0.74 | greenyellow | LTBP1 | 0.80 | salmon | CPNE3 | 0.71 | royalblue | GPR171 | 0.76 | lightcyan | RAB2B | 0.63 | blue | TRAPPC8 | 0.85 | pink | LAT | 0.76 |
| tan | FFAR4 | 0.74 | greenyellow | MRC2 | 0.80 | salmon | DEK | 0.71 | royalblue | PBX4 | 0.76 | lightcyan | GOT2 | 0.62 | blue | MTDH | 0.84 | pink | CCDC69 | 0.76 |
| tan | PEPD | 0.73 | greenyellow | FOXC1 | 0.80 | salmon | ZC3H6 | 0.71 | royalblue | GZMK | 0.76 | lightcyan | PSMF1 | 0.61 | blue | UEVLD | 0.84 | pink | C19orf66 | 0.76 |
| tan | LRPAP1 | 0.73 | greenyellow | CFH | 0.80 | salmon | BIVM | 0.71 | royalblue | KLRB1 | 0.76 | lightcyan | SPRTN | 0.61 | blue | ZMPSTE24 | 0.84 | pink | DIP2A | 0.76 |
| tan | GGTA1P | 0.73 | greenyellow | SNX9 | 0.80 | salmon | NPAT | 0.70 | royalblue | GPR18 | 0.75 | lightcyan | WDR45B | 0.60 | blue | BTBD10 | 0.84 | pink | INTS1 | 0.75 |
| tan | CNDP2 | 0.73 | greenyellow | PTGFRN | 0.80 | salmon | ZNF662 | 0.70 | royalblue | IL2RB | 0.75 | lightcyan | MRPL4 | 0.60 | blue | MYL12A | 0.84 | pink | TNFRSF14 | 0.75 |
| tan | SCCPDH | 0.72 | greenyellow | SERPINH1 | 0.80 | salmon | CNOT2 | 0.70 | royalblue | DGKA | 0.75 | lightcyan | MTMR2 | 0.60 | blue | PQLC3 | 0.84 | pink | BAG6 | 0.75 |
| tan | ZWINT | 0.72 | greenyellow | C7 | 0.80 | salmon | TPR | 0.70 | royalblue | HCP5 | 0.75 | lightcyan | SYS1 | 0.59 | blue | TRAPPC3 | 0.84 | pink | CCDC57 | 0.75 |
| tan | CD74 | 0.72 | greenyellow | PLVAP | 0.80 | salmon | ZC2HC1A | 0.70 | royalblue | CD8A | 0.74 | lightcyan | SGTA | 0.59 | blue | NUP37 | 0.84 | pink | CABIN1 | 0.75 |
| tan | MCEMP1 | 0.72 | greenyellow | ECM1 | 0.80 | salmon | ZNF660 | 0.69 | royalblue | SH2D2A | 0.72 | lightcyan | FAM20B | 0.59 | blue | ABI1 | 0.84 | pink | TUBGCP6 | 0.75 |
| tan | SLC31A1 | 0.72 | greenyellow | PPIC | 0.79 | salmon | IREB2 | 0.69 | royalblue | CDCA7 | 0.72 | lightcyan | EIF3F | 0.57 | blue | FBXL5 | 0.83 | pink | TAZ | 0.75 |
| tan | MSR1 | 0.72 | greenyellow | ZFPM2 | 0.79 | salmon | NSUN6 | 0.69 | royalblue | TRAF5 | 0.72 | lightcyan | SRP68 | 0.57 | blue | SPCS1 | 0.83 | pink | TCOF1 | 0.74 |
| tan | SLFN11 | 0.72 | greenyellow | CPE | 0.79 | salmon | ZNF566 | 0.68 | royalblue | ADA | 0.72 | lightcyan | WIPI2 | 0.56 | blue | GHITM | 0.83 | pink | DNMT1 | 0.74 |
| tan | RDH11 | 0.72 | greenyellow | RSPO3 | 0.79 | salmon | ZNF175 | 0.67 | royalblue | CXCR6 | 0.72 | lightcyan | CCM2 | 0.56 | blue | FAM175B | 0.83 | pink | POLM | 0.74 |
| tan | CORO1C | 0.72 | greenyellow | SPRY1 | 0.79 | salmon | ZNF451 | 0.67 | royalblue | ZC3H12D | 0.71 | lightcyan | MICU1 | 0.55 | blue | VCPIP1 | 0.83 | pink | ZFPM1 | 0.74 |
| tan | CCDC88A | 0.72 | greenyellow | SULF1 | 0.79 | salmon | ZBTB41 | 0.67 | royalblue | APOBEC3H | 0.69 | lightcyan | PSEN2 | 0.54 | blue | UBA3 | 0.83 | pink | RAB1B | 0.74 |
| tan | RBP4 | 0.71 | greenyellow | BNC2 | 0.79 | salmon | PTCD3 | 0.67 | royalblue | SLAMF7 | 0.69 | lightcyan | SHMT2 | 0.54 | blue | ZNF207 | 0.83 | pink | FAM193B | 0.74 |
| tan | TMED3 | 0.71 | greenyellow | LOXL1 | 0.79 | salmon | GOLGB1 | 0.67 | royalblue | LAX1 | 0.68 | lightcyan | OCRL | 0.53 | blue | IST1 | 0.83 | pink | SELENOO | 0.74 |
| tan | GGA2 | 0.71 | greenyellow | CTSK | 0.79 | salmon | GCC2 | 0.67 | royalblue | CD27 | 0.67 | lightcyan | DUSP22 | 0.50 | blue | CELF1 | 0.83 | pink | VPS39 | 0.74 |
| tan | CXCL16 | 0.71 | greenyellow | SCARA5 | 0.79 | salmon | FANCL | 0.67 | royalblue | GZMH | 0.67 | lightcyan | KDELR2 | 0.49 | blue | RER1 | 0.83 | pink | C1orf35 | 0.74 |
| tan | ABCG1 | 0.71 | greenyellow | SELENOM | 0.79 | salmon | CSNK1G3 | 0.67 | royalblue | PIM2 | 0.66 | lightcyan | TUBB | 0.47 | blue | AP5M1 | 0.83 | pink | ZNF276 | 0.73 |
| tan | LGMN | 0.71 | greenyellow | SEMA3D | 0.79 | salmon | CDK14 | 0.66 | royalblue | LY9 | 0.64 | lightcyan | TSPAN4 | 0.40 | blue | ETFA | 0.82 | pink | NDOR1 | 0.73 |
| tan | FUCA2 | 0.70 | greenyellow | PTGIS | 0.79 | salmon | BUD13 | 0.66 | royalblue | TBX21 | 0.61 | lightcyan | C1R | 0.19 | blue | STAG2 | 0.82 | pink | KDM5C | 0.73 |
| tan | CDCP1 | 0.70 | greenyellow | LOXL2 | 0.78 | salmon | USP47 | 0.66 | royalblue | CXCR3 | 0.61 | lightcyan | FAM200B | -0.40 | blue | C5orf15 | 0.82 | pink | PCED1A | 0.73 |
| tan | ABCC3 | 0.70 | greenyellow | KDELR3 | 0.78 | salmon | FRG1 | 0.66 | royalblue | CTLA4 | 0.58 | lightcyan | CIR1 | -0.50 | blue | DCTN4 | 0.82 | pink | USF2 | 0.73 |
| tan | VDAC1 | 0.70 | greenyellow | EPHA3 | 0.78 | salmon | UBE2E3 | 0.66 | royalblue | CD40 | -0.06 | lightcyan | SNORD67 | -0.52 | blue | TOR1AIP1 | 0.82 | pink | YIPF3 | 0.73 |
| tan | HLA-DOA | 0.70 | greenyellow | FBXL7 | 0.78 | salmon | ATG5 | 0.66 |  |  |  | lightcyan | NFU1 | -0.54 | blue | C3orf38 | 0.82 | pink | GATA3 | 0.73 |
| tan | VAMP8 | 0.70 | greenyellow | GXYLT2 | 0.78 | salmon | ORC3 | 0.65 |  |  |  | lightcyan | AHI1 | -0.54 | blue | RAB5C | 0.82 | pink | MPHOSPH8 | 0.73 |
| tan | SLC47A1 | 0.69 | greenyellow | PTGDS | 0.78 | salmon | USP9X | 0.65 |  |  |  | lightcyan | ZNF527 | -0.54 | blue | SRSF1 | 0.82 | pink | TTC31 | 0.73 |
| tan | SUCNR1 | 0.69 | greenyellow | GPC6 | 0.78 | salmon | RUFY2 | 0.65 |  |  |  | lightcyan | NAV2 | -0.55 | blue | SEC24C | 0.82 | pink | PAN2 | 0.72 |
| tan | CLIP4 | 0.69 | greenyellow | PDGFRL | 0.77 | salmon | DOPEY1 | 0.65 |  |  |  | lightcyan | NDUFAF7 | -0.55 | blue | UNC93B1 | 0.82 | pink | TNK2 | 0.72 |
| tan | ACER3 | 0.69 | greenyellow | HSPB1 | 0.77 | salmon | RTTN | 0.64 |  |  |  | lightcyan | NCOA2 | -0.56 | blue | STX7 | 0.82 | pink | GSK3A | 0.72 |
| tan | SCD | 0.69 | greenyellow | NID2 | 0.77 | salmon | NEMF | 0.64 |  |  |  | lightcyan | RUFY3 | -0.57 | blue | PTGES3 | 0.82 | pink | ABHD10 | 0.72 |
| tan | SLAMF8 | 0.68 | greenyellow | LTBP2 | 0.77 | salmon | DMTF1 | 0.64 |  |  |  | lightcyan | ANKRD36B | -0.57 | blue | MAPRE1 | 0.81 | pink | DENND4B | 0.72 |
| tan | PCOLCE2 | 0.68 | greenyellow | ITM2C | 0.77 | salmon | GKAP1 | 0.64 |  |  |  | lightcyan | WDR19 | -0.58 | blue | SEC23A | 0.81 | pink | PPP1R35 | 0.72 |
| tan | HMMR | 0.68 | greenyellow | IGDCC4 | 0.77 | salmon | ZNF540 | 0.64 |  |  |  | lightcyan | PCGF3 | -0.58 | blue | RAD21 | 0.81 | pink | TFAP4 | 0.72 |
| tan | PHLDA3 | 0.68 | greenyellow | PAMR1 | 0.77 | salmon | NR2C1 | 0.63 |  |  |  | lightcyan | CMYA5 | -0.58 | blue | CAT | 0.81 | pink | ZFYVE27 | 0.72 |
| tan | SHTN1 | 0.67 | greenyellow | FAP | 0.77 | salmon | DND1 | 0.63 |  |  |  | lightcyan | HNRNPA2B1 | -0.59 | blue | GNB1 | 0.81 | pink | NPFF | 0.71 |
| tan | STEAP3 | 0.67 | greenyellow | DCN | 0.77 | salmon | CPEB2 | 0.63 |  |  |  | lightcyan | DYNLT1 | -0.60 | blue | SLC30A5 | 0.81 | pink | STK11 | 0.71 |
| tan | TTC39B | 0.67 | greenyellow | LOX | 0.76 | salmon | CCDC43 | 0.63 |  |  |  | lightcyan | MEA1 | -0.60 | blue | DENND1B | 0.81 | pink | ACSF3 | 0.71 |
| tan | FOLR2 | 0.67 | greenyellow | GAS1 | 0.76 | salmon | SYCP2 | 0.63 |  |  |  | lightcyan | RGPD4 | -0.60 | blue | RAP1A | 0.81 | pink | STK4 | 0.71 |
| tan | MYDGF | 0.67 | greenyellow | ECM2 | 0.76 | salmon | SETD4 | 0.63 |  |  |  | lightcyan | CCDC14 | -0.60 | blue | MAP3K2 | 0.81 | pink | METTL17 | 0.71 |
| tan | DNAJC5B | 0.66 | greenyellow | THY1 | 0.76 | salmon | CEP295 | 0.63 |  |  |  | lightcyan | GPATCH2L | -0.60 | blue | GFM2 | 0.81 | pink | SH2B1 | 0.71 |
| tan | DDB2 | 0.66 | greenyellow | NFASC | 0.76 | salmon | TCF12 | 0.63 |  |  |  | lightcyan | CWC22 | -0.60 | blue | CGGBP1 | 0.81 | pink | EXOC3 | 0.71 |
| tan | LACTB2 | 0.66 | greenyellow | TMEM119 | 0.76 | salmon | ZNF37A | 0.62 |  |  |  | lightcyan | MRPS21 | -0.61 | blue | PCMT1 | 0.81 | pink | SPOP | 0.71 |
| tan | NMB | 0.66 | greenyellow | COL6A2 | 0.75 | salmon | GOLGA8A | 0.62 |  |  |  | lightcyan | NFX1 | -0.61 | blue | TMED10 | 0.81 | pink | OBSCN | 0.71 |
| tan | FABP4 | 0.66 | greenyellow | ROR2 | 0.75 | salmon | FRA10AC1 | 0.62 |  |  |  | lightcyan | NDUFS5 | -0.61 | blue | DENR | 0.81 | pink | CEP135 | 0.71 |
| tan | ACOT4 | 0.65 | greenyellow | LUM | 0.75 | salmon | IGF2BP2 | 0.62 |  |  |  | lightcyan | TMA7 | -0.61 | blue | NUDT5 | 0.80 | pink | OGG1 | 0.71 |
| tan | ACP2 | 0.65 | greenyellow | LRRN4CL | 0.74 | salmon | COX11 | 0.62 |  |  |  | lightcyan | ANKRD10 | -0.61 | blue | YIPF5 | 0.80 | pink | SCAP | 0.71 |
| tan | FDFT1 | 0.65 | greenyellow | MT-TF | 0.74 | salmon | KMT5B | 0.62 |  |  |  | lightcyan | MAP3K7 | -0.61 | blue | ACOT9 | 0.80 | pink | MAEA | 0.70 |
| tan | PCLAF | 0.65 | greenyellow | CD34 | 0.73 | salmon | ZNF418 | 0.62 |  |  |  | lightcyan | WDR6 | -0.63 | blue | TMEM167B | 0.80 | pink | SOX12 | 0.70 |
| tan | TMEM144 | 0.65 | greenyellow | MXRA5 | 0.73 | salmon | ZSCAN30 | 0.62 |  |  |  | lightcyan | VPS13A | -0.63 | blue | TMEM126B | 0.80 | pink | ZNF440 | 0.70 |
| tan | SPR | 0.64 | greenyellow | COL18A1 | 0.73 | salmon | FBXO25 | 0.61 |  |  |  | lightcyan | WDR26 | -0.64 | blue | TMEM50A | 0.80 | pink | GALT | 0.70 |
| tan | ANLN | 0.64 | greenyellow | FRZB | 0.73 | salmon | CARF | 0.61 |  |  |  | lightcyan | ZFC3H1 | -0.64 | blue | SKAP2 | 0.80 | pink | PPP6R2 | 0.70 |
| tan | MAN1A1 | 0.64 | greenyellow | SAMD11 | 0.73 | salmon | CLASP2 | 0.61 |  |  |  | lightcyan | FAM96B | -0.64 | blue | SSR1 | 0.80 | pink | PCNX3 | 0.70 |
| tan | CD163L1 | 0.64 | greenyellow | SNCAIP | 0.72 | salmon | HMGB1 | 0.61 |  |  |  | lightcyan | ORMDL1 | -0.64 | blue | TRAPPC11 | 0.80 | pink | INTS3 | 0.69 |
| tan | ARSB | 0.63 | greenyellow | PLPPR4 | 0.72 | salmon | ARMCX3 | 0.61 |  |  |  | lightcyan | ZNF23 | -0.65 | blue | ARMC10 | 0.79 | pink | ARFGAP2 | 0.69 |
| tan | NANS | 0.63 | greenyellow | LRRC17 | 0.72 | salmon | AQR | 0.61 |  |  |  | lightcyan | GTF2H1 | -0.65 | blue | THAP12 | 0.79 | pink | UBAP2L | 0.69 |
| tan | CDK1 | 0.63 | greenyellow | CTHRC1 | 0.72 | salmon | HECTD2 | 0.60 |  |  |  | lightcyan | OFD1 | -0.65 | blue | PSMD10 | 0.79 | pink | PHF12 | 0.69 |
| tan | PPA2 | 0.63 | greenyellow | GSN | 0.72 | salmon | ZC3HC1 | 0.60 |  |  |  | lightcyan | NLK | -0.66 | blue | NBR1 | 0.79 | pink | TRERF1 | 0.69 |
| tan | CST3 | 0.63 | greenyellow | ZNF521 | 0.72 | salmon | NIPBL | 0.60 |  |  |  | lightcyan | GPATCH2 | -0.66 | blue | TXNDC12 | 0.79 | pink | GAK | 0.69 |
| tan | ALAS1 | 0.63 | greenyellow | OSR2 | 0.72 | salmon | KIAA1143 | 0.60 |  |  |  | lightcyan | EWSR1 | -0.66 | blue | SHOC2 | 0.79 | pink | CIB1 | 0.69 |
| tan | CD80 | 0.62 | greenyellow | DNM1 | 0.72 | salmon | UBXN7 | 0.60 |  |  |  | lightcyan | MDN1 | -0.66 | blue | UBE2I | 0.79 | pink | RAD9A | 0.69 |
| tan | OSCAR | 0.62 | greenyellow | COL1A1 | 0.71 | salmon | ESCO1 | 0.60 |  |  |  | lightcyan | AAGAB | -0.67 | blue | IMPA1 | 0.79 | pink | DHPS | 0.69 |
| tan | HLA-DQA2 | 0.62 | greenyellow | FOXRED2 | 0.71 | salmon | TYW5 | 0.60 |  |  |  | lightcyan | ABCA5 | -0.67 | blue | LAMP2 | 0.79 | pink | LZTR1 | 0.69 |
| tan | DDO | 0.62 | greenyellow | IGFBP6 | 0.71 | salmon | XKR6 | 0.59 |  |  |  | lightcyan | RBX1 | -0.68 | blue | UHMK1 | 0.79 | pink | SETDB1 | 0.69 |
| tan | MPND | 0.62 | greenyellow | ADCY3 | 0.71 | salmon | RNF8 | 0.59 |  |  |  | lightcyan | PSMG4 | -0.68 | blue | ARL6IP1 | 0.79 | pink | TMUB1 | 0.69 |
| tan | PCNA | 0.62 | greenyellow | MFAP2 | 0.71 | salmon | CAPRIN2 | 0.59 |  |  |  | lightcyan | LZTS3 | -0.69 | blue | ARCN1 | 0.79 | pink | SH3BP5L | 0.68 |
| tan | NR1H3 | 0.62 | greenyellow | PARD6G | 0.71 | salmon | ZCCHC7 | 0.59 |  |  |  | lightcyan | AGO3 | -0.70 | blue | HBP1 | 0.79 | pink | FLYWCH1 | 0.68 |
| tan | TEX14 | 0.61 | greenyellow | GPX8 | 0.71 | salmon | ING5 | 0.59 |  |  |  | lightcyan | PCBP2 | -0.71 | blue | RNF6 | 0.79 | pink | LZIC | 0.68 |
| tan | NAGLU | 0.61 | greenyellow | GPRC5B | 0.70 | salmon | ZNF3 | 0.59 |  |  |  | lightcyan | CCDC183 | -0.72 | blue | NCOR1 | 0.79 | pink | DEPDC5 | 0.68 |
| tan | TRIM36 | 0.61 | greenyellow | AOX1 | 0.70 | salmon | WDR11 | 0.59 |  |  |  | lightcyan | RBM6 | -0.72 | blue | NRDC | 0.79 | pink | OSBPL7 | 0.68 |
| tan | TYMS | 0.61 | greenyellow | DCLK1 | 0.70 | salmon | APPL1 | 0.59 |  |  |  | lightcyan | GLMN | -0.73 | blue | RAP1B | 0.78 | pink | IQSEC1 | 0.68 |
| tan | KLHDC8B | 0.61 | greenyellow | SFRP2 | 0.70 | salmon | ZNF761 | 0.59 |  |  |  | lightcyan | EMG1 | -0.73 | blue | SFT2D1 | 0.78 | pink | RBCK1 | 0.68 |
| tan | OPN3 | 0.61 | greenyellow | OMD | 0.69 | salmon | SS18L1 | 0.58 |  |  |  | lightcyan | GABBR1 | -0.74 | blue | RAD23B | 0.78 | pink | TYK2 | 0.68 |
| tan | APOC2 | 0.61 | greenyellow | OSBPL10 | 0.69 | salmon | ARSD | 0.58 |  |  |  | lightcyan | ITSN2 | -0.75 | blue | UBE2K | 0.78 | pink | AAK1 | 0.68 |
| tan | AGPAT2 | 0.60 | greenyellow | B3GNT9 | 0.69 | salmon | ZNF682 | 0.58 |  |  |  | lightcyan | CCNL2 | -0.76 | blue | NIPA2 | 0.78 | pink | ZFAT | 0.67 |
| tan | PNPLA6 | 0.60 | greenyellow | PDE1A | 0.69 | salmon | BAZ1B | 0.58 |  |  |  | lightcyan | RPAIN | -0.76 | blue | SRP72 | 0.78 | pink | KIAA0907 | 0.67 |
| tan | IL18 | 0.59 | greenyellow | OAF | 0.68 | salmon | EXTL2 | 0.57 |  |  |  | lightcyan | HIST2H2AC | -0.77 | blue | NIPSNAP3A | 0.78 | pink | MINK1 | 0.67 |
| tan | TMEM53 | 0.59 | greenyellow | FMO1 | 0.68 | salmon | ZNF558 | 0.57 |  |  |  | lightcyan | ATP5I | -0.83 | blue | MRPS30 | 0.78 | pink | MAN2C1 | 0.67 |
| tan | GAS2L3 | 0.59 | greenyellow | SRPX2 | 0.67 | salmon | GGPS1 | 0.57 |  |  |  |  |  |  | blue | RRM2B | 0.78 | pink | TTC38 | 0.67 |
| tan | ATP6V1F | 0.59 | greenyellow | SFRP1 | 0.67 | salmon | ZNF548 | 0.57 |  |  |  |  |  |  | blue | TM2D3 | 0.78 | pink | NSUN5P1 | 0.67 |
| tan | NHLRC3 | 0.59 | greenyellow | RCN3 | 0.66 | salmon | LRP6 | 0.57 |  |  |  |  |  |  | blue | SNW1 | 0.77 | pink | TRIM38 | 0.67 |
| tan | LIMK1 | 0.59 | greenyellow | SFRP4 | 0.66 | salmon | RFC1 | 0.57 |  |  |  |  |  |  | blue | DSCR3 | 0.77 | pink | LMF2 | 0.67 |
| tan | NARS | 0.59 | greenyellow | C1QTNF3 | 0.65 | salmon | EIF1AD | 0.57 |  |  |  |  |  |  | blue | TMEM59 | 0.77 | pink | TADA3 | 0.67 |
| tan | C15orf48 | 0.59 | greenyellow | ESR1 | 0.65 | salmon | FAM160B1 | 0.56 |  |  |  |  |  |  | blue | CLDND1 | 0.77 | pink | PAK2 | 0.67 |
| tan | PLD3 | 0.58 | greenyellow | CHID1 | 0.65 | salmon | RICTOR | 0.56 |  |  |  |  |  |  | blue | USP8 | 0.77 | pink | HIST1H1D | 0.67 |
| tan | SERINC2 | 0.58 | greenyellow | CILP | 0.65 | salmon | XRN1 | 0.56 |  |  |  |  |  |  | blue | CPSF2 | 0.77 | pink | ELMOD3 | 0.67 |
| tan | SPNS1 | 0.58 | greenyellow | DBN1 | 0.64 | salmon | TIPRL | 0.56 |  |  |  |  |  |  | blue | APMAP | 0.77 | pink | NPRL2 | 0.67 |
| tan | ICMT | 0.58 | greenyellow | GLI2 | 0.64 | salmon | PUS7L | 0.56 |  |  |  |  |  |  | blue | SEC24B | 0.77 | pink | SMG9 | 0.66 |
| tan | RAB42 | 0.58 | greenyellow | ZBTB47 | 0.63 | salmon | C21orf91 | 0.56 |  |  |  |  |  |  | blue | ARPC5 | 0.77 | pink | UBTF | 0.66 |
| tan | LHFPL2 | 0.58 | greenyellow | ADM5 | 0.63 | salmon | ZNF547 | 0.56 |  |  |  |  |  |  | blue | ERGIC1 | 0.77 | pink | SCARF2 | 0.66 |
| tan | DCSTAMP | 0.58 | greenyellow | KIAA1211 | 0.63 | salmon | AMDHD1 | 0.55 |  |  |  |  |  |  | blue | U2SURP | 0.77 | pink | LEMD2 | 0.66 |
| tan | TM7SF3 | 0.57 | greenyellow | TENM3 | 0.62 | salmon | ZNF471 | 0.55 |  |  |  |  |  |  | blue | VAMP3 | 0.76 | pink | MTRF1 | 0.66 |
| tan | PRC1 | 0.57 | greenyellow | IL17RD | 0.62 | salmon | RREB1 | 0.54 |  |  |  |  |  |  | blue | UBE2V2 | 0.76 | pink | IKBKB | 0.66 |
| tan | AKAP5 | 0.57 | greenyellow | MEOX1 | 0.61 | salmon | BDP1 | 0.54 |  |  |  |  |  |  | blue | NUP160 | 0.76 | pink | UNC13D | 0.66 |
| tan | CD209 | 0.56 | greenyellow | ANGPT2 | 0.61 | salmon | ZNF789 | 0.54 |  |  |  |  |  |  | blue | RTFDC1 | 0.76 | pink | APBA3 | 0.66 |
| tan | FAM102B | 0.56 | greenyellow | BOC | 0.61 | salmon | PIWIL4 | 0.53 |  |  |  |  |  |  | blue | MFN2 | 0.76 | pink | SART1 | 0.66 |
| tan | MRPS27 | 0.55 | greenyellow | EGFLAM | 0.61 | salmon | TMEM123 | 0.52 |  |  |  |  |  |  | blue | NAP1L4 | 0.76 | pink | LRRC14 | 0.66 |
| tan | AGPAT4 | 0.55 | greenyellow | SMO | 0.60 | salmon | POGZ | 0.52 |  |  |  |  |  |  | blue | PPP3CA | 0.76 | pink | ARHGAP25 | 0.66 |
| tan | HSD3B7 | 0.55 | greenyellow | HOXD8 | 0.60 | salmon | ZNF629 | 0.52 |  |  |  |  |  |  | blue | SLBP | 0.76 | pink | FAM78A | 0.66 |
| tan | PLEKHA7 | 0.55 | greenyellow | CLEC11A | 0.60 | salmon | PEX1 | 0.52 |  |  |  |  |  |  | blue | ZNF791 | 0.76 | pink | FAM120B | 0.65 |
| tan | ACYP2 | 0.55 | greenyellow | QSOX1 | 0.60 | salmon | NINL | 0.52 |  |  |  |  |  |  | blue | ARFGEF1 | 0.76 | pink | LPCAT4 | 0.65 |
| tan | VRK2 | 0.55 | greenyellow | AK4 | 0.60 | salmon | SGCD | 0.51 |  |  |  |  |  |  | blue | PLEKHF2 | 0.76 | pink | HPS1 | 0.65 |
| tan | DEFB1 | 0.55 | greenyellow | TSHZ2 | 0.59 | salmon | PYGB | 0.51 |  |  |  |  |  |  | blue | ZDHHC17 | 0.76 | pink | VPS18 | 0.65 |
| tan | KIF16B | 0.54 | greenyellow | PAPPA | 0.59 | salmon | ICE2 | 0.50 |  |  |  |  |  |  | blue | KIAA1429 | 0.76 | pink | E2F7 | 0.65 |
| tan | HFE | 0.54 | greenyellow | MZB1 | 0.59 | salmon | ZNF33A | 0.50 |  |  |  |  |  |  | blue | ARMC1 | 0.76 | pink | NELFCD | 0.65 |
| tan | RETN | 0.53 | greenyellow | SPAG4 | 0.59 | salmon | FAM169A | 0.49 |  |  |  |  |  |  | blue | RIOK3 | 0.76 | pink | SETD1A | 0.65 |
| tan | INHBA | 0.53 | greenyellow | CXCL14 | 0.59 | salmon | KDM5A | 0.48 |  |  |  |  |  |  | blue | TMEM33 | 0.75 | pink | ZNF337 | 0.65 |
| tan | IL17RB | 0.53 | greenyellow | ADRA2A | 0.59 | salmon | RSBN1 | 0.48 |  |  |  |  |  |  | blue | IQGAP1 | 0.75 | pink | AZU1 | 0.65 |
| tan | CRIP1 | 0.53 | greenyellow | DERL3 | 0.59 | salmon | BOD1L1 | 0.46 |  |  |  |  |  |  | blue | MRPL44 | 0.75 | pink | TRAPPC9 | 0.65 |
| tan | TCF7L2 | 0.53 | greenyellow | UCHL1 | 0.58 | salmon | PRPF38B | 0.43 |  |  |  |  |  |  | blue | TAB2 | 0.75 | pink | SEC31B | 0.65 |
| tan | FAM89A | 0.52 | greenyellow | MYRIP | 0.58 | salmon | KATNBL1 | 0.41 |  |  |  |  |  |  | blue | YWHAZ | 0.75 | pink | ARID1A | 0.65 |
| tan | SULT1C2 | 0.51 | greenyellow | TUBB3 | 0.58 | salmon | MAMDC2 | 0.37 |  |  |  |  |  |  | blue | POLD3 | 0.75 | pink | MROH6 | 0.65 |
| tan | TRIM6 | 0.51 | greenyellow | KIF26B | 0.57 | salmon | SP1 | 0.37 |  |  |  |  |  |  | blue | PSMB2 | 0.74 | pink | CCDC34 | 0.65 |
| tan | SRD5A3 | 0.51 | greenyellow | P3H4 | 0.57 | salmon | ZNF641 | 0.31 |  |  |  |  |  |  | blue | TFDP1 | 0.74 | pink | BTN3A1 | 0.64 |
| tan | MRPS25 | 0.51 | greenyellow | FNDC1 | 0.57 | salmon | ASNS | 0.30 |  |  |  |  |  |  | blue | TMCO3 | 0.74 | pink | INO80E | 0.64 |
| tan | SLC7A8 | 0.49 | greenyellow | SLC16A2 | 0.57 | salmon | CYLD | 0.28 |  |  |  |  |  |  | blue | FBXL17 | 0.74 | pink | AKR1E2 | 0.64 |
| tan | GPCPD1 | 0.49 | greenyellow | CPXM1 | 0.56 | salmon | SAMD3 | 0.28 |  |  |  |  |  |  | blue | CDC42SE2 | 0.74 | pink | C9orf142 | 0.64 |
| tan | AGRP | 0.48 | greenyellow | GFRA2 | 0.56 | salmon | LBR | 0.11 |  |  |  |  |  |  | blue | BECN1 | 0.74 | pink | STYXL1 | 0.64 |
| tan | MRPS15 | 0.48 | greenyellow | SNAP25 | 0.55 | salmon | ARFGEF2 | -0.44 |  |  |  |  |  |  | blue | RNF13 | 0.74 | pink | ATP13A2 | 0.64 |
| tan | VMO1 | 0.48 | greenyellow | SCN1B | 0.55 | salmon | FAM168B | -0.48 |  |  |  |  |  |  | blue | DBR1 | 0.74 | pink | STX1B | 0.63 |
| tan | C1orf122 | 0.44 | greenyellow | SPOCK1 | 0.55 | salmon | NPLOC4 | -0.49 |  |  |  |  |  |  | blue | ZNF611 | 0.74 | pink | PGGHG | 0.63 |
| tan | SPOCD1 | 0.43 | greenyellow | DOK5 | 0.54 | salmon | SLC39A1 | -0.51 |  |  |  |  |  |  | blue | AP1M1 | 0.74 | pink | VILL | 0.63 |
| tan | GPA33 | 0.43 | greenyellow | CD99 | 0.54 | salmon | TBC1D9 | -0.51 |  |  |  |  |  |  | blue | PLPBP | 0.74 | pink | SNAPC4 | 0.63 |
| tan | PUSL1 | 0.39 | greenyellow | AMPD1 | 0.54 | salmon | ARF3 | -0.52 |  |  |  |  |  |  | blue | SEPHS2 | 0.73 | pink | MED12 | 0.63 |
| tan | DPP7 | 0.38 | greenyellow | TNFRSF17 | 0.54 | salmon | ALDH18A1 | -0.54 |  |  |  |  |  |  | blue | SLC25A3 | 0.73 | pink | SP110 | 0.63 |
| tan | GRB2 | 0.25 | greenyellow | NOVA1 | 0.54 | salmon | CHMP4B | -0.55 |  |  |  |  |  |  | blue | SCAF8 | 0.73 | pink | C17orf67 | 0.63 |
| tan | LILRB1 | 0.22 | greenyellow | ADAMTSL1 | 0.54 | salmon | SWAP70 | -0.56 |  |  |  |  |  |  | blue | DAD1 | 0.73 | pink | IQSEC2 | 0.63 |
| tan | MAPKAPK3 | 0.22 | greenyellow | PDGFD | 0.54 | salmon | DUSP1 | -0.61 |  |  |  |  |  |  | blue | MSN | 0.73 | pink | RPP25L | 0.62 |
| tan | ATP6V0B | 0.20 | greenyellow | CYTL1 | 0.53 | salmon | CALR | -0.61 |  |  |  |  |  |  | blue | ITCH | 0.73 | pink | TPCN2 | 0.62 |
| tan | KHDRBS3 | -0.01 | greenyellow | CDKN2C | 0.53 | salmon | NARF | -0.63 |  |  |  |  |  |  | blue | COPS5 | 0.73 | pink | AIP | 0.62 |
| tan | PGM3 | -0.04 | greenyellow | ZNF385D | 0.52 | salmon | ERO1A | -0.64 |  |  |  |  |  |  | blue | NDUFA5 | 0.73 | pink | UBXN11 | 0.62 |
| tan | TGIF2 | -0.45 | greenyellow | LDLRAD3 | 0.52 | salmon | PKM | -0.64 |  |  |  |  |  |  | blue | FBXL3 | 0.73 | pink | CSAD | 0.62 |
| tan | PDE1C | -0.48 | greenyellow | VASH2 | 0.52 | salmon | CDV3 | -0.66 |  |  |  |  |  |  | blue | TSPYL1 | 0.73 | pink | HAGH | 0.62 |
| tan | CDC42EP1 | -0.48 | greenyellow | PLA2G2A | 0.51 | salmon | ADSS | -0.69 |  |  |  |  |  |  | blue | CNOT7 | 0.73 | pink | IRF3 | 0.62 |
| tan | DEFA5 | -0.50 | greenyellow | P3H1 | 0.51 | salmon | SH3GLB1 | -0.70 |  |  |  |  |  |  | blue | XPO7 | 0.73 | pink | MDM4 | 0.62 |
| tan | MECP2 | -0.51 | greenyellow | HRC | 0.50 | salmon | UBE2A | -0.76 |  |  |  |  |  |  | blue | TXNDC9 | 0.73 | pink | MED16 | 0.61 |
| tan | PHLPP1 | -0.54 | greenyellow | PCDH19 | 0.50 | salmon | SNRPB | -0.84 |  |  |  |  |  |  | blue | AKTIP | 0.73 | pink | DPH7 | 0.61 |
| tan | SUPT6H | -0.54 | greenyellow | SMPDL3A | 0.49 |  |  |  |  |  |  |  |  |  | blue | SLC25A46 | 0.73 | pink | WDR27 | 0.61 |
| tan | RPL37 | -0.55 | greenyellow | POU2AF1 | 0.49 |  |  |  |  |  |  |  |  |  | blue | XRN2 | 0.72 | pink | SLC6A19 | 0.61 |
| tan | AMN1 | -0.56 | greenyellow | EDA2R | 0.49 |  |  |  |  |  |  |  |  |  | blue | COG5 | 0.72 | pink | NMRAL1 | 0.61 |
| tan | SF1 | -0.57 | greenyellow | CCDC178 | 0.48 |  |  |  |  |  |  |  |  |  | blue | ANP32A | 0.72 | pink | CCDC130 | 0.61 |
| tan | NEUROD4 | -0.59 | greenyellow | MMP16 | 0.47 |  |  |  |  |  |  |  |  |  | blue | LTN1 | 0.72 | pink | ALDH16A1 | 0.61 |
| tan | EXTL3 | -0.59 | greenyellow | BEND6 | 0.47 |  |  |  |  |  |  |  |  |  | blue | COMMD10 | 0.72 | pink | CACNB1 | 0.61 |
| tan | CACNG8 | -0.59 | greenyellow | FAM198B | 0.46 |  |  |  |  |  |  |  |  |  | blue | EIF4G2 | 0.72 | pink | REST | 0.61 |
| tan | ING3 | -0.60 | greenyellow | FAM43B | 0.46 |  |  |  |  |  |  |  |  |  | blue | POT1 | 0.72 | pink | ZNF862 | 0.60 |
| tan | ETS1 | -0.60 | greenyellow | PI16 | 0.46 |  |  |  |  |  |  |  |  |  | blue | HNRNPDL | 0.72 | pink | CRAMP1 | 0.60 |
| tan | CSNK1D | -0.60 | greenyellow | MFAP5 | 0.46 |  |  |  |  |  |  |  |  |  | blue | MRPL19 | 0.72 | pink | CC2D1A | 0.60 |
| tan | METTL9 | -0.65 | greenyellow | GREB1 | 0.45 |  |  |  |  |  |  |  |  |  | blue | COA1 | 0.72 | pink | COA5 | 0.60 |
| tan | IRF2BP1 | -0.67 | greenyellow | NLGN1 | 0.44 |  |  |  |  |  |  |  |  |  | blue | CUL2 | 0.72 | pink | PIP5K1C | 0.60 |
|  |  |  | greenyellow | SDK1 | 0.44 |  |  |  |  |  |  |  |  |  | blue | PPP4R3B | 0.72 | pink | ABHD8 | 0.60 |
|  |  |  | greenyellow | TWIST1 | 0.43 |  |  |  |  |  |  |  |  |  | blue | TSG101 | 0.72 | pink | PRRT3 | 0.60 |
|  |  |  | greenyellow | GLI1 | 0.42 |  |  |  |  |  |  |  |  |  | blue | ATF1 | 0.72 | pink | C14orf180 | 0.60 |
|  |  |  | greenyellow | HOXC4 | 0.41 |  |  |  |  |  |  |  |  |  | blue | PTPN12 | 0.72 | pink | ZBTB7B | 0.60 |
|  |  |  | greenyellow | TMEM132C | 0.41 |  |  |  |  |  |  |  |  |  | blue | CNOT10 | 0.71 | pink | NOXA1 | 0.60 |
|  |  |  | greenyellow | TUBB2B | 0.40 |  |  |  |  |  |  |  |  |  | blue | DNAJC14 | 0.71 | pink | CCDC166 | 0.60 |
|  |  |  | greenyellow | ATP6V1G2 | 0.39 |  |  |  |  |  |  |  |  |  | blue | R3HCC1L | 0.71 | pink | HEXDC | 0.59 |
|  |  |  | greenyellow | SLN | 0.39 |  |  |  |  |  |  |  |  |  | blue | ASH2L | 0.71 | pink | UBR4 | 0.59 |
|  |  |  | greenyellow | CASC10 | 0.35 |  |  |  |  |  |  |  |  |  | blue | DNAJB6 | 0.71 | pink | GSDMD | 0.59 |
|  |  |  | greenyellow | ZNF469 | 0.32 |  |  |  |  |  |  |  |  |  | blue | MSL2 | 0.71 | pink | TELO2 | 0.59 |
|  |  |  | greenyellow | COL24A1 | 0.30 |  |  |  |  |  |  |  |  |  | blue | ZBTB1 | 0.71 | pink | AKAP8L | 0.59 |
|  |  |  | greenyellow | GAS7 | 0.20 |  |  |  |  |  |  |  |  |  | blue | SCAMP2 | 0.71 | pink | MYOZ3 | 0.59 |
|  |  |  | greenyellow | SULF2 | 0.10 |  |  |  |  |  |  |  |  |  | blue | ZBTB11 | 0.71 | pink | LDB1 | 0.59 |
|  |  |  | greenyellow | VCAN | -0.04 |  |  |  |  |  |  |  |  |  | blue | PIGF | 0.71 | pink | MARCH9 | 0.58 |
|  |  |  | greenyellow | TAF11 | -0.46 |  |  |  |  |  |  |  |  |  | blue | TOMM70 | 0.71 | pink | NCDN | 0.58 |
|  |  |  | greenyellow | MME | -0.63 |  |  |  |  |  |  |  |  |  | blue | CCT5 | 0.70 | pink | TTLL3 | 0.58 |
|  |  |  |  |  |  |  |  |  |  |  |  |  |  |  | blue | SERP1 | 0.70 | pink | SSH3 | 0.58 |
|  |  |  |  |  |  |  |  |  |  |  |  |  |  |  | blue | JKAMP | 0.70 | pink | FMNL3 | 0.58 |
|  |  |  |  |  |  |  |  |  |  |  |  |  |  |  | blue | LPCAT3 | 0.70 | pink | SLX4 | 0.58 |
|  |  |  |  |  |  |  |  |  |  |  |  |  |  |  | blue | RPL7L1 | 0.70 | pink | ENO3 | 0.57 |
|  |  |  |  |  |  |  |  |  |  |  |  |  |  |  | blue | PRPSAP2 | 0.70 | pink | COMMD3 | 0.57 |
|  |  |  |  |  |  |  |  |  |  |  |  |  |  |  | blue | ANP32E | 0.70 | pink | MAX | 0.55 |
|  |  |  |  |  |  |  |  |  |  |  |  |  |  |  | blue | WDR82 | 0.70 | pink | HIST1H1E | 0.55 |
|  |  |  |  |  |  |  |  |  |  |  |  |  |  |  | blue | PUM2 | 0.70 | pink | HES7 | 0.55 |
|  |  |  |  |  |  |  |  |  |  |  |  |  |  |  | blue | ZDHHC20 | 0.70 | pink | UHRF2 | 0.55 |
|  |  |  |  |  |  |  |  |  |  |  |  |  |  |  | blue | TMEM167A | 0.70 | pink | ARID3B | 0.54 |
|  |  |  |  |  |  |  |  |  |  |  |  |  |  |  | blue | CNOT8 | 0.70 | pink | LRFN3 | 0.53 |
|  |  |  |  |  |  |  |  |  |  |  |  |  |  |  | blue | BTF3L4 | 0.69 | pink | RPA2 | 0.52 |
|  |  |  |  |  |  |  |  |  |  |  |  |  |  |  | blue | SNX3 | 0.69 | pink | ECHDC2 | 0.33 |
|  |  |  |  |  |  |  |  |  |  |  |  |  |  |  | blue | GPR160 | 0.69 | pink | DVL1 | 0.33 |
|  |  |  |  |  |  |  |  |  |  |  |  |  |  |  | blue | MRPL18 | 0.69 | pink | EPB41L1 | 0.32 |
|  |  |  |  |  |  |  |  |  |  |  |  |  |  |  | blue | EID2 | 0.69 | pink | ECI1 | 0.29 |
|  |  |  |  |  |  |  |  |  |  |  |  |  |  |  | blue | PIGM | 0.69 | pink | ASAP3 | 0.13 |
|  |  |  |  |  |  |  |  |  |  |  |  |  |  |  | blue | MARCH5 | 0.69 | pink | HNRNPD | -0.48 |
|  |  |  |  |  |  |  |  |  |  |  |  |  |  |  | blue | CBFB | 0.69 | pink | RPL35A | -0.54 |
|  |  |  |  |  |  |  |  |  |  |  |  |  |  |  | blue | CRKL | 0.69 | pink | UTP15 | -0.54 |
|  |  |  |  |  |  |  |  |  |  |  |  |  |  |  | blue | GPBP1L1 | 0.69 | pink | EIF5 | -0.57 |
|  |  |  |  |  |  |  |  |  |  |  |  |  |  |  | blue | PTPRA | 0.69 | pink | TCEAL9 | -0.57 |
|  |  |  |  |  |  |  |  |  |  |  |  |  |  |  | blue | SERBP1 | 0.69 | pink | RETREG3 | -0.57 |
|  |  |  |  |  |  |  |  |  |  |  |  |  |  |  | blue | PAFAH1B2 | 0.69 | pink | HSPE1 | -0.58 |
|  |  |  |  |  |  |  |  |  |  |  |  |  |  |  | blue | CDYL | 0.69 | pink | UBR1 | -0.58 |
|  |  |  |  |  |  |  |  |  |  |  |  |  |  |  | blue | FBXW11 | 0.68 | pink | EIF4G1 | -0.58 |
|  |  |  |  |  |  |  |  |  |  |  |  |  |  |  | blue | INIP | 0.68 | pink | SPG20 | -0.59 |
|  |  |  |  |  |  |  |  |  |  |  |  |  |  |  | blue | CASP3 | 0.68 | pink | PRDX2 | -0.59 |
|  |  |  |  |  |  |  |  |  |  |  |  |  |  |  | blue | ZDHHC7 | 0.68 | pink | CKS1B | -0.60 |
|  |  |  |  |  |  |  |  |  |  |  |  |  |  |  | blue | NT5C2 | 0.68 | pink | DPH2 | -0.60 |
|  |  |  |  |  |  |  |  |  |  |  |  |  |  |  | blue | HNRNPH3 | 0.68 | pink | G3BP1 | -0.61 |
|  |  |  |  |  |  |  |  |  |  |  |  |  |  |  | blue | AP3M1 | 0.68 | pink | RIOX2 | -0.61 |
|  |  |  |  |  |  |  |  |  |  |  |  |  |  |  | blue | ARV1 | 0.68 | pink | TAF13 | -0.61 |
|  |  |  |  |  |  |  |  |  |  |  |  |  |  |  | blue | BRCC3 | 0.68 | pink | FBXO34 | -0.62 |
|  |  |  |  |  |  |  |  |  |  |  |  |  |  |  | blue | PRKAR2A | 0.68 | pink | ADO | -0.62 |
|  |  |  |  |  |  |  |  |  |  |  |  |  |  |  | blue | TMOD3 | 0.68 | pink | BLOC1S6 | -0.62 |
|  |  |  |  |  |  |  |  |  |  |  |  |  |  |  | blue | SLC9A6 | 0.68 | pink | TMEM168 | -0.63 |
|  |  |  |  |  |  |  |  |  |  |  |  |  |  |  | blue | ALG8 | 0.68 | pink | PEX3 | -0.63 |
|  |  |  |  |  |  |  |  |  |  |  |  |  |  |  | blue | FCF1 | 0.68 | pink | UBTD2 | -0.64 |
|  |  |  |  |  |  |  |  |  |  |  |  |  |  |  | blue | SBNO1 | 0.68 | pink | SPCS2 | -0.66 |
|  |  |  |  |  |  |  |  |  |  |  |  |  |  |  | blue | MFF | 0.68 | pink | MAPK1IP1L | -0.66 |
|  |  |  |  |  |  |  |  |  |  |  |  |  |  |  | blue | CIAO1 | 0.67 | pink | NGDN | -0.66 |
|  |  |  |  |  |  |  |  |  |  |  |  |  |  |  | blue | GPN1 | 0.67 | pink | AHCYL1 | -0.67 |
|  |  |  |  |  |  |  |  |  |  |  |  |  |  |  | blue | DHX40 | 0.67 | pink | IMPAD1 | -0.67 |
|  |  |  |  |  |  |  |  |  |  |  |  |  |  |  | blue | COL4A3BP | 0.67 | pink | EEF1A1 | -0.67 |
|  |  |  |  |  |  |  |  |  |  |  |  |  |  |  | blue | RPF1 | 0.67 | pink | UFM1 | -0.68 |
|  |  |  |  |  |  |  |  |  |  |  |  |  |  |  | blue | KDM3B | 0.67 | pink | TARS | -0.68 |
|  |  |  |  |  |  |  |  |  |  |  |  |  |  |  | blue | LAMTOR3 | 0.67 | pink | SPAG9 | -0.68 |
|  |  |  |  |  |  |  |  |  |  |  |  |  |  |  | blue | TRIP12 | 0.67 | pink | TCEA1 | -0.69 |
|  |  |  |  |  |  |  |  |  |  |  |  |  |  |  | blue | IMP3 | 0.67 | pink | PSMD7 | -0.69 |
|  |  |  |  |  |  |  |  |  |  |  |  |  |  |  | blue | OAZ2 | 0.66 | pink | NUP54 | -0.69 |
|  |  |  |  |  |  |  |  |  |  |  |  |  |  |  | blue | XRCC5 | 0.66 | pink | DPY19L1 | -0.69 |
|  |  |  |  |  |  |  |  |  |  |  |  |  |  |  | blue | KPNB1 | 0.66 | pink | SLC30A9 | -0.70 |
|  |  |  |  |  |  |  |  |  |  |  |  |  |  |  | blue | SLC35E1 | 0.66 | pink | PSMD2 | -0.71 |
|  |  |  |  |  |  |  |  |  |  |  |  |  |  |  | blue | UBE3C | 0.66 | pink | ACBD3 | -0.72 |
|  |  |  |  |  |  |  |  |  |  |  |  |  |  |  | blue | ZYG11B | 0.66 | pink | CCDC6 | -0.72 |
|  |  |  |  |  |  |  |  |  |  |  |  |  |  |  | blue | GATC | 0.66 | pink | RNF19A | -0.72 |
|  |  |  |  |  |  |  |  |  |  |  |  |  |  |  | blue | ZFAND1 | 0.66 | pink | RRN3 | -0.72 |
|  |  |  |  |  |  |  |  |  |  |  |  |  |  |  | blue | PIK3CA | 0.66 | pink | CD59 | -0.72 |
|  |  |  |  |  |  |  |  |  |  |  |  |  |  |  | blue | VTI1B | 0.66 | pink | STARD4 | -0.73 |
|  |  |  |  |  |  |  |  |  |  |  |  |  |  |  | blue | THUMPD1 | 0.66 | pink | NRAS | -0.73 |
|  |  |  |  |  |  |  |  |  |  |  |  |  |  |  | blue | SENP2 | 0.66 | pink | SELENOS | -0.73 |
|  |  |  |  |  |  |  |  |  |  |  |  |  |  |  | blue | PECR | 0.66 | pink | DNAJB9 | -0.73 |
|  |  |  |  |  |  |  |  |  |  |  |  |  |  |  | blue | SRSF5 | 0.65 | pink | IDH3A | -0.73 |
|  |  |  |  |  |  |  |  |  |  |  |  |  |  |  | blue | ZBTB33 | 0.65 | pink | CDKL3 | -0.74 |
|  |  |  |  |  |  |  |  |  |  |  |  |  |  |  | blue | MRPS22 | 0.65 | pink | HSP90AA1 | -0.74 |
|  |  |  |  |  |  |  |  |  |  |  |  |  |  |  | blue | METAP1 | 0.65 | pink | SS18 | -0.74 |
|  |  |  |  |  |  |  |  |  |  |  |  |  |  |  | blue | ATP5S | 0.65 | pink | CCNYL1 | -0.75 |
|  |  |  |  |  |  |  |  |  |  |  |  |  |  |  | blue | CMTR2 | 0.65 | pink | PRPS1 | -0.75 |
|  |  |  |  |  |  |  |  |  |  |  |  |  |  |  | blue | AMACR | 0.65 | pink | TPM4 | -0.75 |
|  |  |  |  |  |  |  |  |  |  |  |  |  |  |  | blue | ADAT1 | 0.65 | pink | YWHAG | -0.76 |
|  |  |  |  |  |  |  |  |  |  |  |  |  |  |  | blue | AKT1 | 0.65 | pink | BTBD1 | -0.76 |
|  |  |  |  |  |  |  |  |  |  |  |  |  |  |  | blue | PDCD10 | 0.65 | pink | CHCHD7 | -0.76 |
|  |  |  |  |  |  |  |  |  |  |  |  |  |  |  | blue | BAG4 | 0.65 | pink | FASTKD2 | -0.77 |
|  |  |  |  |  |  |  |  |  |  |  |  |  |  |  | blue | RAPGEF6 | 0.65 | pink | XBP1 | -0.77 |
|  |  |  |  |  |  |  |  |  |  |  |  |  |  |  | blue | PITPNA | 0.65 | pink | NCBP2 | -0.78 |
|  |  |  |  |  |  |  |  |  |  |  |  |  |  |  | blue | PPP6C | 0.64 | pink | TRA2B | -0.78 |
|  |  |  |  |  |  |  |  |  |  |  |  |  |  |  | blue | KMT2E | 0.64 | pink | TIMM17A | -0.79 |
|  |  |  |  |  |  |  |  |  |  |  |  |  |  |  | blue | ZFR | 0.64 | pink | EIF3I | -0.80 |
|  |  |  |  |  |  |  |  |  |  |  |  |  |  |  | blue | ATF2 | 0.64 | pink | IPO7 | -0.81 |
|  |  |  |  |  |  |  |  |  |  |  |  |  |  |  | blue | APOL2 | 0.64 | pink | SEC63 | -0.82 |
|  |  |  |  |  |  |  |  |  |  |  |  |  |  |  | blue | TNPO1 | 0.64 | pink | PSMD11 | -0.82 |
|  |  |  |  |  |  |  |  |  |  |  |  |  |  |  | blue | HSD17B12 | 0.64 | pink | PRKRA | -0.83 |
|  |  |  |  |  |  |  |  |  |  |  |  |  |  |  | blue | NRBF2 | 0.64 | pink | ARF4 | -0.83 |
|  |  |  |  |  |  |  |  |  |  |  |  |  |  |  | blue | HSBP1 | 0.64 | pink | BZW1 | -0.84 |
|  |  |  |  |  |  |  |  |  |  |  |  |  |  |  | blue | OGFOD1 | 0.63 | pink | METAP2 | -0.86 |
|  |  |  |  |  |  |  |  |  |  |  |  |  |  |  | blue | CDC40 | 0.63 | pink | YWHAQ | -0.86 |
|  |  |  |  |  |  |  |  |  |  |  |  |  |  |  | blue | TRPM7 | 0.63 | pink | HNRNPF | -0.86 |
|  |  |  |  |  |  |  |  |  |  |  |  |  |  |  | blue | RNF114 | 0.63 | pink | DHX15 | -0.86 |
|  |  |  |  |  |  |  |  |  |  |  |  |  |  |  | blue | SF3B1 | 0.63 | pink | PRNP | -0.89 |
|  |  |  |  |  |  |  |  |  |  |  |  |  |  |  | blue | AGTPBP1 | 0.63 | pink | CCDC47 | -0.94 |
|  |  |  |  |  |  |  |  |  |  |  |  |  |  |  | blue | TLK1 | 0.63 |  |  |  |
|  |  |  |  |  |  |  |  |  |  |  |  |  |  |  | blue | CKAP2 | 0.63 |  |  |  |
|  |  |  |  |  |  |  |  |  |  |  |  |  |  |  | blue | EIF3A | 0.63 |  |  |  |
|  |  |  |  |  |  |  |  |  |  |  |  |  |  |  | blue | CLPX | 0.63 |  |  |  |
|  |  |  |  |  |  |  |  |  |  |  |  |  |  |  | blue | CNOT1 | 0.62 |  |  |  |
|  |  |  |  |  |  |  |  |  |  |  |  |  |  |  | blue | RCOR1 | 0.62 |  |  |  |
|  |  |  |  |  |  |  |  |  |  |  |  |  |  |  | blue | SSNA1 | 0.62 |  |  |  |
|  |  |  |  |  |  |  |  |  |  |  |  |  |  |  | blue | ENTPD4 | 0.62 |  |  |  |
|  |  |  |  |  |  |  |  |  |  |  |  |  |  |  | blue | QRSL1 | 0.62 |  |  |  |
|  |  |  |  |  |  |  |  |  |  |  |  |  |  |  | blue | CASC4 | 0.62 |  |  |  |
|  |  |  |  |  |  |  |  |  |  |  |  |  |  |  | blue | SLC39A9 | 0.62 |  |  |  |
|  |  |  |  |  |  |  |  |  |  |  |  |  |  |  | blue | USP25 | 0.62 |  |  |  |
|  |  |  |  |  |  |  |  |  |  |  |  |  |  |  | blue | NAA50 | 0.62 |  |  |  |
|  |  |  |  |  |  |  |  |  |  |  |  |  |  |  | blue | TRPC4AP | 0.62 |  |  |  |
|  |  |  |  |  |  |  |  |  |  |  |  |  |  |  | blue | NKRF | 0.62 |  |  |  |
|  |  |  |  |  |  |  |  |  |  |  |  |  |  |  | blue | B3GNT2 | 0.62 |  |  |  |
|  |  |  |  |  |  |  |  |  |  |  |  |  |  |  | blue | ZNF765 | 0.62 |  |  |  |
|  |  |  |  |  |  |  |  |  |  |  |  |  |  |  | blue | SKIV2L2 | 0.62 |  |  |  |
|  |  |  |  |  |  |  |  |  |  |  |  |  |  |  | blue | DNAJB14 | 0.62 |  |  |  |
|  |  |  |  |  |  |  |  |  |  |  |  |  |  |  | blue | STX11 | 0.62 |  |  |  |
|  |  |  |  |  |  |  |  |  |  |  |  |  |  |  | blue | GLTP | 0.61 |  |  |  |
|  |  |  |  |  |  |  |  |  |  |  |  |  |  |  | blue | SLC35A5 | 0.61 |  |  |  |
|  |  |  |  |  |  |  |  |  |  |  |  |  |  |  | blue | SEC11C | 0.61 |  |  |  |
|  |  |  |  |  |  |  |  |  |  |  |  |  |  |  | blue | YTHDF1 | 0.61 |  |  |  |
|  |  |  |  |  |  |  |  |  |  |  |  |  |  |  | blue | ZNF24 | 0.61 |  |  |  |
|  |  |  |  |  |  |  |  |  |  |  |  |  |  |  | blue | ACLY | 0.61 |  |  |  |
|  |  |  |  |  |  |  |  |  |  |  |  |  |  |  | blue | INSIG2 | 0.61 |  |  |  |
|  |  |  |  |  |  |  |  |  |  |  |  |  |  |  | blue | TSNAX | 0.60 |  |  |  |
|  |  |  |  |  |  |  |  |  |  |  |  |  |  |  | blue | CLINT1 | 0.60 |  |  |  |
|  |  |  |  |  |  |  |  |  |  |  |  |  |  |  | blue | USP38 | 0.60 |  |  |  |
|  |  |  |  |  |  |  |  |  |  |  |  |  |  |  | blue | GALK2 | 0.60 |  |  |  |
|  |  |  |  |  |  |  |  |  |  |  |  |  |  |  | blue | TMEM50B | 0.60 |  |  |  |
|  |  |  |  |  |  |  |  |  |  |  |  |  |  |  | blue | FEM1B | 0.60 |  |  |  |
|  |  |  |  |  |  |  |  |  |  |  |  |  |  |  | blue | HACD4 | 0.60 |  |  |  |
|  |  |  |  |  |  |  |  |  |  |  |  |  |  |  | blue | DTX3L | 0.60 |  |  |  |
|  |  |  |  |  |  |  |  |  |  |  |  |  |  |  | blue | PDE6D | 0.60 |  |  |  |
|  |  |  |  |  |  |  |  |  |  |  |  |  |  |  | blue | ZNF92 | 0.60 |  |  |  |
|  |  |  |  |  |  |  |  |  |  |  |  |  |  |  | blue | C1D | 0.60 |  |  |  |
|  |  |  |  |  |  |  |  |  |  |  |  |  |  |  | blue | DIRC2 | 0.60 |  |  |  |
|  |  |  |  |  |  |  |  |  |  |  |  |  |  |  | blue | OXSR1 | 0.60 |  |  |  |
|  |  |  |  |  |  |  |  |  |  |  |  |  |  |  | blue | SPTY2D1 | 0.60 |  |  |  |
|  |  |  |  |  |  |  |  |  |  |  |  |  |  |  | blue | HERPUD2 | 0.60 |  |  |  |
|  |  |  |  |  |  |  |  |  |  |  |  |  |  |  | blue | PRPF38A | 0.59 |  |  |  |
|  |  |  |  |  |  |  |  |  |  |  |  |  |  |  | blue | SEC24D | 0.59 |  |  |  |
|  |  |  |  |  |  |  |  |  |  |  |  |  |  |  | blue | RNASEH1 | 0.59 |  |  |  |
|  |  |  |  |  |  |  |  |  |  |  |  |  |  |  | blue | SCO1 | 0.59 |  |  |  |
|  |  |  |  |  |  |  |  |  |  |  |  |  |  |  | blue | KDM4C | 0.59 |  |  |  |
|  |  |  |  |  |  |  |  |  |  |  |  |  |  |  | blue | PATL1 | 0.59 |  |  |  |
|  |  |  |  |  |  |  |  |  |  |  |  |  |  |  | blue | UCHL5 | 0.59 |  |  |  |
|  |  |  |  |  |  |  |  |  |  |  |  |  |  |  | blue | ZDHHC2 | 0.59 |  |  |  |
|  |  |  |  |  |  |  |  |  |  |  |  |  |  |  | blue | MTMR12 | 0.59 |  |  |  |
|  |  |  |  |  |  |  |  |  |  |  |  |  |  |  | blue | ANKRD13A | 0.58 |  |  |  |
|  |  |  |  |  |  |  |  |  |  |  |  |  |  |  | blue | DLG1 | 0.58 |  |  |  |
|  |  |  |  |  |  |  |  |  |  |  |  |  |  |  | blue | TM9SF4 | 0.58 |  |  |  |
|  |  |  |  |  |  |  |  |  |  |  |  |  |  |  | blue | ZNF143 | 0.58 |  |  |  |
|  |  |  |  |  |  |  |  |  |  |  |  |  |  |  | blue | KIDINS220 | 0.58 |  |  |  |
|  |  |  |  |  |  |  |  |  |  |  |  |  |  |  | blue | WNK1 | 0.58 |  |  |  |
|  |  |  |  |  |  |  |  |  |  |  |  |  |  |  | blue | THAP1 | 0.58 |  |  |  |
|  |  |  |  |  |  |  |  |  |  |  |  |  |  |  | blue | PDCD2 | 0.58 |  |  |  |
|  |  |  |  |  |  |  |  |  |  |  |  |  |  |  | blue | BROX | 0.58 |  |  |  |
|  |  |  |  |  |  |  |  |  |  |  |  |  |  |  | blue | TBCE | 0.58 |  |  |  |
|  |  |  |  |  |  |  |  |  |  |  |  |  |  |  | blue | PHF11 | 0.58 |  |  |  |
|  |  |  |  |  |  |  |  |  |  |  |  |  |  |  | blue | ZNF770 | 0.58 |  |  |  |
|  |  |  |  |  |  |  |  |  |  |  |  |  |  |  | blue | LRRC41 | 0.58 |  |  |  |
|  |  |  |  |  |  |  |  |  |  |  |  |  |  |  | blue | ABHD17B | 0.57 |  |  |  |
|  |  |  |  |  |  |  |  |  |  |  |  |  |  |  | blue | MRPL58 | 0.57 |  |  |  |
|  |  |  |  |  |  |  |  |  |  |  |  |  |  |  | blue | SLC30A6 | 0.57 |  |  |  |
|  |  |  |  |  |  |  |  |  |  |  |  |  |  |  | blue | CUL4A | 0.57 |  |  |  |
|  |  |  |  |  |  |  |  |  |  |  |  |  |  |  | blue | AK2 | 0.57 |  |  |  |
|  |  |  |  |  |  |  |  |  |  |  |  |  |  |  | blue | YY1AP1 | 0.57 |  |  |  |
|  |  |  |  |  |  |  |  |  |  |  |  |  |  |  | blue | C5orf51 | 0.57 |  |  |  |
|  |  |  |  |  |  |  |  |  |  |  |  |  |  |  | blue | TNKS2 | 0.57 |  |  |  |
|  |  |  |  |  |  |  |  |  |  |  |  |  |  |  | blue | KPNA1 | 0.56 |  |  |  |
|  |  |  |  |  |  |  |  |  |  |  |  |  |  |  | blue | BICD2 | 0.56 |  |  |  |
|  |  |  |  |  |  |  |  |  |  |  |  |  |  |  | blue | TMEM209 | 0.56 |  |  |  |
|  |  |  |  |  |  |  |  |  |  |  |  |  |  |  | blue | SNX4 | 0.56 |  |  |  |
|  |  |  |  |  |  |  |  |  |  |  |  |  |  |  | blue | GBE1 | 0.56 |  |  |  |
|  |  |  |  |  |  |  |  |  |  |  |  |  |  |  | blue | NECAP1 | 0.56 |  |  |  |
|  |  |  |  |  |  |  |  |  |  |  |  |  |  |  | blue | ZMYM2 | 0.56 |  |  |  |
|  |  |  |  |  |  |  |  |  |  |  |  |  |  |  | blue | EPC2 | 0.56 |  |  |  |
|  |  |  |  |  |  |  |  |  |  |  |  |  |  |  | blue | PRKAA1 | 0.56 |  |  |  |
|  |  |  |  |  |  |  |  |  |  |  |  |  |  |  | blue | RAB5B | 0.56 |  |  |  |
|  |  |  |  |  |  |  |  |  |  |  |  |  |  |  | blue | CBX3 | 0.56 |  |  |  |
|  |  |  |  |  |  |  |  |  |  |  |  |  |  |  | blue | C5orf24 | 0.56 |  |  |  |
|  |  |  |  |  |  |  |  |  |  |  |  |  |  |  | blue | ABHD18 | 0.56 |  |  |  |
|  |  |  |  |  |  |  |  |  |  |  |  |  |  |  | blue | CCDC117 | 0.55 |  |  |  |
|  |  |  |  |  |  |  |  |  |  |  |  |  |  |  | blue | TLK2 | 0.55 |  |  |  |
|  |  |  |  |  |  |  |  |  |  |  |  |  |  |  | blue | GALNT10 | 0.55 |  |  |  |
|  |  |  |  |  |  |  |  |  |  |  |  |  |  |  | blue | FUBP1 | 0.55 |  |  |  |
|  |  |  |  |  |  |  |  |  |  |  |  |  |  |  | blue | PLAA | 0.55 |  |  |  |
|  |  |  |  |  |  |  |  |  |  |  |  |  |  |  | blue | BRWD1 | 0.54 |  |  |  |
|  |  |  |  |  |  |  |  |  |  |  |  |  |  |  | blue | RMND1 | 0.54 |  |  |  |
|  |  |  |  |  |  |  |  |  |  |  |  |  |  |  | blue | EIF2AK1 | 0.54 |  |  |  |
|  |  |  |  |  |  |  |  |  |  |  |  |  |  |  | blue | RNF38 | 0.54 |  |  |  |
|  |  |  |  |  |  |  |  |  |  |  |  |  |  |  | blue | KCTD18 | 0.53 |  |  |  |
|  |  |  |  |  |  |  |  |  |  |  |  |  |  |  | blue | TSN | 0.53 |  |  |  |
|  |  |  |  |  |  |  |  |  |  |  |  |  |  |  | blue | UBE2W | 0.53 |  |  |  |
|  |  |  |  |  |  |  |  |  |  |  |  |  |  |  | blue | RBM26 | 0.53 |  |  |  |
|  |  |  |  |  |  |  |  |  |  |  |  |  |  |  | blue | MMGT1 | 0.53 |  |  |  |
|  |  |  |  |  |  |  |  |  |  |  |  |  |  |  | blue | MRPL13 | 0.53 |  |  |  |
|  |  |  |  |  |  |  |  |  |  |  |  |  |  |  | blue | KRAS | 0.53 |  |  |  |
|  |  |  |  |  |  |  |  |  |  |  |  |  |  |  | blue | PRKAG1 | 0.52 |  |  |  |
|  |  |  |  |  |  |  |  |  |  |  |  |  |  |  | blue | MTMR6 | 0.52 |  |  |  |
|  |  |  |  |  |  |  |  |  |  |  |  |  |  |  | blue | YKT6 | 0.52 |  |  |  |
|  |  |  |  |  |  |  |  |  |  |  |  |  |  |  | blue | PTCD2 | 0.52 |  |  |  |
|  |  |  |  |  |  |  |  |  |  |  |  |  |  |  | blue | TRAM1 | 0.52 |  |  |  |
|  |  |  |  |  |  |  |  |  |  |  |  |  |  |  | blue | MFSD14B | 0.52 |  |  |  |
|  |  |  |  |  |  |  |  |  |  |  |  |  |  |  | blue | MRPL48 | 0.52 |  |  |  |
|  |  |  |  |  |  |  |  |  |  |  |  |  |  |  | blue | UBE2H | 0.52 |  |  |  |
|  |  |  |  |  |  |  |  |  |  |  |  |  |  |  | blue | CCDC22 | 0.51 |  |  |  |
|  |  |  |  |  |  |  |  |  |  |  |  |  |  |  | blue | WDR44 | 0.51 |  |  |  |
|  |  |  |  |  |  |  |  |  |  |  |  |  |  |  | blue | MFAP3 | 0.51 |  |  |  |
|  |  |  |  |  |  |  |  |  |  |  |  |  |  |  | blue | PNMA1 | 0.51 |  |  |  |
|  |  |  |  |  |  |  |  |  |  |  |  |  |  |  | blue | PLA2G4A | 0.51 |  |  |  |
|  |  |  |  |  |  |  |  |  |  |  |  |  |  |  | blue | MCM9 | 0.50 |  |  |  |
|  |  |  |  |  |  |  |  |  |  |  |  |  |  |  | blue | RPS6KA3 | 0.50 |  |  |  |
|  |  |  |  |  |  |  |  |  |  |  |  |  |  |  | blue | GAPVD1 | 0.50 |  |  |  |
|  |  |  |  |  |  |  |  |  |  |  |  |  |  |  | blue | TEX10 | 0.49 |  |  |  |
|  |  |  |  |  |  |  |  |  |  |  |  |  |  |  | blue | BRK1 | 0.49 |  |  |  |
|  |  |  |  |  |  |  |  |  |  |  |  |  |  |  | blue | SLC33A1 | 0.48 |  |  |  |
|  |  |  |  |  |  |  |  |  |  |  |  |  |  |  | blue | SLC30A7 | 0.48 |  |  |  |
|  |  |  |  |  |  |  |  |  |  |  |  |  |  |  | blue | KRIT1 | 0.47 |  |  |  |
|  |  |  |  |  |  |  |  |  |  |  |  |  |  |  | blue | UBR7 | 0.47 |  |  |  |
|  |  |  |  |  |  |  |  |  |  |  |  |  |  |  | blue | CTNNA1 | 0.46 |  |  |  |
|  |  |  |  |  |  |  |  |  |  |  |  |  |  |  | blue | CYFIP1 | 0.46 |  |  |  |
|  |  |  |  |  |  |  |  |  |  |  |  |  |  |  | blue | TCEAL8 | 0.46 |  |  |  |
|  |  |  |  |  |  |  |  |  |  |  |  |  |  |  | blue | MRS2 | 0.44 |  |  |  |
|  |  |  |  |  |  |  |  |  |  |  |  |  |  |  | blue | RTCB | 0.43 |  |  |  |
|  |  |  |  |  |  |  |  |  |  |  |  |  |  |  | blue | IRGQ | 0.43 |  |  |  |
|  |  |  |  |  |  |  |  |  |  |  |  |  |  |  | blue | BCAP29 | 0.42 |  |  |  |
|  |  |  |  |  |  |  |  |  |  |  |  |  |  |  | blue | GPHN | 0.37 |  |  |  |
|  |  |  |  |  |  |  |  |  |  |  |  |  |  |  | blue | DAG1 | 0.37 |  |  |  |
|  |  |  |  |  |  |  |  |  |  |  |  |  |  |  | blue | PIGP | 0.34 |  |  |  |
|  |  |  |  |  |  |  |  |  |  |  |  |  |  |  | blue | SCARB2 | 0.33 |  |  |  |
|  |  |  |  |  |  |  |  |  |  |  |  |  |  |  | blue | MGST3 | 0.30 |  |  |  |
|  |  |  |  |  |  |  |  |  |  |  |  |  |  |  | blue | NENF | 0.24 |  |  |  |
|  |  |  |  |  |  |  |  |  |  |  |  |  |  |  | blue | ALDH1A1 | 0.18 |  |  |  |
|  |  |  |  |  |  |  |  |  |  |  |  |  |  |  | blue | PTPRK | -0.01 |  |  |  |
|  |  |  |  |  |  |  |  |  |  |  |  |  |  |  | blue | CCDC88B | -0.19 |  |  |  |
|  |  |  |  |  |  |  |  |  |  |  |  |  |  |  | blue | ATP2A3 | -0.20 |  |  |  |
|  |  |  |  |  |  |  |  |  |  |  |  |  |  |  | blue | KIAA1024 | -0.21 |  |  |  |
|  |  |  |  |  |  |  |  |  |  |  |  |  |  |  | blue | ZNF467 | -0.23 |  |  |  |
|  |  |  |  |  |  |  |  |  |  |  |  |  |  |  | blue | GNG8 | -0.25 |  |  |  |
|  |  |  |  |  |  |  |  |  |  |  |  |  |  |  | blue | PPP6R1 | -0.27 |  |  |  |
|  |  |  |  |  |  |  |  |  |  |  |  |  |  |  | blue | TOP3A | -0.28 |  |  |  |
|  |  |  |  |  |  |  |  |  |  |  |  |  |  |  | blue | NLRP1 | -0.30 |  |  |  |
|  |  |  |  |  |  |  |  |  |  |  |  |  |  |  | blue | XRCC3 | -0.31 |  |  |  |
|  |  |  |  |  |  |  |  |  |  |  |  |  |  |  | blue | ATG2A | -0.31 |  |  |  |
|  |  |  |  |  |  |  |  |  |  |  |  |  |  |  | blue | SLC5A10 | -0.33 |  |  |  |
|  |  |  |  |  |  |  |  |  |  |  |  |  |  |  | blue | LTB4R | -0.35 |  |  |  |
|  |  |  |  |  |  |  |  |  |  |  |  |  |  |  | blue | ULK1 | -0.36 |  |  |  |
|  |  |  |  |  |  |  |  |  |  |  |  |  |  |  | blue | KDM4B | -0.39 |  |  |  |
|  |  |  |  |  |  |  |  |  |  |  |  |  |  |  | blue | MMP11 | -0.41 |  |  |  |
|  |  |  |  |  |  |  |  |  |  |  |  |  |  |  | blue | SYNDIG1 | -0.42 |  |  |  |
|  |  |  |  |  |  |  |  |  |  |  |  |  |  |  | blue | STOML1 | -0.43 |  |  |  |
|  |  |  |  |  |  |  |  |  |  |  |  |  |  |  | blue | TMEM91 | -0.44 |  |  |  |
|  |  |  |  |  |  |  |  |  |  |  |  |  |  |  | blue | RASA3 | -0.44 |  |  |  |
|  |  |  |  |  |  |  |  |  |  |  |  |  |  |  | blue | CIDEC | -0.45 |  |  |  |
|  |  |  |  |  |  |  |  |  |  |  |  |  |  |  | blue | ABCC8 | -0.46 |  |  |  |
|  |  |  |  |  |  |  |  |  |  |  |  |  |  |  | blue | ATP1B2 | -0.46 |  |  |  |
|  |  |  |  |  |  |  |  |  |  |  |  |  |  |  | blue | C19orf25 | -0.46 |  |  |  |
|  |  |  |  |  |  |  |  |  |  |  |  |  |  |  | blue | LAMB2 | -0.46 |  |  |  |
|  |  |  |  |  |  |  |  |  |  |  |  |  |  |  | blue | U2AF1L4 | -0.46 |  |  |  |
|  |  |  |  |  |  |  |  |  |  |  |  |  |  |  | blue | TBX10 | -0.46 |  |  |  |
|  |  |  |  |  |  |  |  |  |  |  |  |  |  |  | blue | SOX11 | -0.47 |  |  |  |
|  |  |  |  |  |  |  |  |  |  |  |  |  |  |  | blue | KCNB1 | -0.48 |  |  |  |
|  |  |  |  |  |  |  |  |  |  |  |  |  |  |  | blue | LGI1 | -0.48 |  |  |  |
|  |  |  |  |  |  |  |  |  |  |  |  |  |  |  | blue | OSBPL5 | -0.48 |  |  |  |
|  |  |  |  |  |  |  |  |  |  |  |  |  |  |  | blue | RHBDL1 | -0.48 |  |  |  |
|  |  |  |  |  |  |  |  |  |  |  |  |  |  |  | blue | UGT3A1 | -0.48 |  |  |  |
|  |  |  |  |  |  |  |  |  |  |  |  |  |  |  | blue | TEKT4 | -0.49 |  |  |  |
|  |  |  |  |  |  |  |  |  |  |  |  |  |  |  | blue | SLC25A28 | -0.49 |  |  |  |
|  |  |  |  |  |  |  |  |  |  |  |  |  |  |  | blue | TMEM74 | -0.49 |  |  |  |
|  |  |  |  |  |  |  |  |  |  |  |  |  |  |  | blue | RANGRF | -0.49 |  |  |  |
|  |  |  |  |  |  |  |  |  |  |  |  |  |  |  | blue | PHC1 | -0.49 |  |  |  |
|  |  |  |  |  |  |  |  |  |  |  |  |  |  |  | blue | NTF3 | -0.49 |  |  |  |
|  |  |  |  |  |  |  |  |  |  |  |  |  |  |  | blue | ABCD4 | -0.49 |  |  |  |
|  |  |  |  |  |  |  |  |  |  |  |  |  |  |  | blue | FAM13A | -0.49 |  |  |  |
|  |  |  |  |  |  |  |  |  |  |  |  |  |  |  | blue | GADD45G | -0.50 |  |  |  |
|  |  |  |  |  |  |  |  |  |  |  |  |  |  |  | blue | NUP85 | -0.50 |  |  |  |
|  |  |  |  |  |  |  |  |  |  |  |  |  |  |  | blue | CLIP3 | -0.51 |  |  |  |
|  |  |  |  |  |  |  |  |  |  |  |  |  |  |  | blue | KCNT1 | -0.51 |  |  |  |
|  |  |  |  |  |  |  |  |  |  |  |  |  |  |  | blue | TAF1C | -0.51 |  |  |  |
|  |  |  |  |  |  |  |  |  |  |  |  |  |  |  | blue | OR52N4 | -0.52 |  |  |  |
|  |  |  |  |  |  |  |  |  |  |  |  |  |  |  | blue | RBM20 | -0.52 |  |  |  |
|  |  |  |  |  |  |  |  |  |  |  |  |  |  |  | blue | BANF1 | -0.52 |  |  |  |
|  |  |  |  |  |  |  |  |  |  |  |  |  |  |  | blue | LINC01166 | -0.52 |  |  |  |
|  |  |  |  |  |  |  |  |  |  |  |  |  |  |  | blue | CRAT | -0.52 |  |  |  |
|  |  |  |  |  |  |  |  |  |  |  |  |  |  |  | blue | AIRE | -0.52 |  |  |  |
|  |  |  |  |  |  |  |  |  |  |  |  |  |  |  | blue | COL7A1 | -0.52 |  |  |  |
|  |  |  |  |  |  |  |  |  |  |  |  |  |  |  | blue | NUTF2 | -0.52 |  |  |  |
|  |  |  |  |  |  |  |  |  |  |  |  |  |  |  | blue | ADPRHL2 | -0.53 |  |  |  |
|  |  |  |  |  |  |  |  |  |  |  |  |  |  |  | blue | BCR | -0.53 |  |  |  |
|  |  |  |  |  |  |  |  |  |  |  |  |  |  |  | blue | KIZ | -0.53 |  |  |  |
|  |  |  |  |  |  |  |  |  |  |  |  |  |  |  | blue | BFSP1 | -0.53 |  |  |  |
|  |  |  |  |  |  |  |  |  |  |  |  |  |  |  | blue | EIF2B5 | -0.53 |  |  |  |
|  |  |  |  |  |  |  |  |  |  |  |  |  |  |  | blue | GNRH1 | -0.53 |  |  |  |
|  |  |  |  |  |  |  |  |  |  |  |  |  |  |  | blue | PIDD1 | -0.53 |  |  |  |
|  |  |  |  |  |  |  |  |  |  |  |  |  |  |  | blue | SRGAP1 | -0.53 |  |  |  |
|  |  |  |  |  |  |  |  |  |  |  |  |  |  |  | blue | FN3K | -0.53 |  |  |  |
|  |  |  |  |  |  |  |  |  |  |  |  |  |  |  | blue | BNC1 | -0.53 |  |  |  |
|  |  |  |  |  |  |  |  |  |  |  |  |  |  |  | blue | SHC2 | -0.53 |  |  |  |
|  |  |  |  |  |  |  |  |  |  |  |  |  |  |  | blue | CAPN15 | -0.53 |  |  |  |
|  |  |  |  |  |  |  |  |  |  |  |  |  |  |  | blue | SDHAF4 | -0.53 |  |  |  |
|  |  |  |  |  |  |  |  |  |  |  |  |  |  |  | blue | ADAT2 | -0.53 |  |  |  |
|  |  |  |  |  |  |  |  |  |  |  |  |  |  |  | blue | HYAL3 | -0.53 |  |  |  |
|  |  |  |  |  |  |  |  |  |  |  |  |  |  |  | blue | SLC25A47 | -0.53 |  |  |  |
|  |  |  |  |  |  |  |  |  |  |  |  |  |  |  | blue | POLR2A | -0.53 |  |  |  |
|  |  |  |  |  |  |  |  |  |  |  |  |  |  |  | blue | TOB2 | -0.53 |  |  |  |
|  |  |  |  |  |  |  |  |  |  |  |  |  |  |  | blue | ACAP3 | -0.53 |  |  |  |
|  |  |  |  |  |  |  |  |  |  |  |  |  |  |  | blue | DDX56 | -0.53 |  |  |  |
|  |  |  |  |  |  |  |  |  |  |  |  |  |  |  | blue | CDK9 | -0.53 |  |  |  |
|  |  |  |  |  |  |  |  |  |  |  |  |  |  |  | blue | CPT1C | -0.54 |  |  |  |
|  |  |  |  |  |  |  |  |  |  |  |  |  |  |  | blue | SLC9A5 | -0.54 |  |  |  |
|  |  |  |  |  |  |  |  |  |  |  |  |  |  |  | blue | TYW1B | -0.54 |  |  |  |
|  |  |  |  |  |  |  |  |  |  |  |  |  |  |  | blue | NUTM2G | -0.54 |  |  |  |
|  |  |  |  |  |  |  |  |  |  |  |  |  |  |  | blue | FBXO27 | -0.54 |  |  |  |
|  |  |  |  |  |  |  |  |  |  |  |  |  |  |  | blue | ZFP36L1 | -0.54 |  |  |  |
|  |  |  |  |  |  |  |  |  |  |  |  |  |  |  | blue | ANKRD36 | -0.54 |  |  |  |
|  |  |  |  |  |  |  |  |  |  |  |  |  |  |  | blue | ITGA7 | -0.54 |  |  |  |
|  |  |  |  |  |  |  |  |  |  |  |  |  |  |  | blue | NTHL1 | -0.55 |  |  |  |
|  |  |  |  |  |  |  |  |  |  |  |  |  |  |  | blue | CCDC102A | -0.55 |  |  |  |
|  |  |  |  |  |  |  |  |  |  |  |  |  |  |  | blue | CIB2 | -0.55 |  |  |  |
|  |  |  |  |  |  |  |  |  |  |  |  |  |  |  | blue | CDH23 | -0.55 |  |  |  |
|  |  |  |  |  |  |  |  |  |  |  |  |  |  |  | blue | GAS6 | -0.55 |  |  |  |
|  |  |  |  |  |  |  |  |  |  |  |  |  |  |  | blue | DHX38 | -0.55 |  |  |  |
|  |  |  |  |  |  |  |  |  |  |  |  |  |  |  | blue | PAK3 | -0.55 |  |  |  |
|  |  |  |  |  |  |  |  |  |  |  |  |  |  |  | blue | RCE1 | -0.55 |  |  |  |
|  |  |  |  |  |  |  |  |  |  |  |  |  |  |  | blue | TBC1D16 | -0.55 |  |  |  |
|  |  |  |  |  |  |  |  |  |  |  |  |  |  |  | blue | IRF2BP2 | -0.55 |  |  |  |
|  |  |  |  |  |  |  |  |  |  |  |  |  |  |  | blue | LENG9 | -0.55 |  |  |  |
|  |  |  |  |  |  |  |  |  |  |  |  |  |  |  | blue | C21orf2 | -0.55 |  |  |  |
|  |  |  |  |  |  |  |  |  |  |  |  |  |  |  | blue | ALKBH6 | -0.55 |  |  |  |
|  |  |  |  |  |  |  |  |  |  |  |  |  |  |  | blue | C11orf84 | -0.56 |  |  |  |
|  |  |  |  |  |  |  |  |  |  |  |  |  |  |  | blue | OBSL1 | -0.56 |  |  |  |
|  |  |  |  |  |  |  |  |  |  |  |  |  |  |  | blue | TEAD3 | -0.56 |  |  |  |
|  |  |  |  |  |  |  |  |  |  |  |  |  |  |  | blue | KRT33B | -0.56 |  |  |  |
|  |  |  |  |  |  |  |  |  |  |  |  |  |  |  | blue | PRPH | -0.56 |  |  |  |
|  |  |  |  |  |  |  |  |  |  |  |  |  |  |  | blue | PYY2 | -0.56 |  |  |  |
|  |  |  |  |  |  |  |  |  |  |  |  |  |  |  | blue | LRRC14B | -0.56 |  |  |  |
|  |  |  |  |  |  |  |  |  |  |  |  |  |  |  | blue | MRNIP | -0.57 |  |  |  |
|  |  |  |  |  |  |  |  |  |  |  |  |  |  |  | blue | ZCWPW1 | -0.57 |  |  |  |
|  |  |  |  |  |  |  |  |  |  |  |  |  |  |  | blue | NARFL | -0.57 |  |  |  |
|  |  |  |  |  |  |  |  |  |  |  |  |  |  |  | blue | B9D1 | -0.57 |  |  |  |
|  |  |  |  |  |  |  |  |  |  |  |  |  |  |  | blue | ANKRD35 | -0.57 |  |  |  |
|  |  |  |  |  |  |  |  |  |  |  |  |  |  |  | blue | PRRT2 | -0.57 |  |  |  |
|  |  |  |  |  |  |  |  |  |  |  |  |  |  |  | blue | MROH7 | -0.57 |  |  |  |
|  |  |  |  |  |  |  |  |  |  |  |  |  |  |  | blue | HAUS7 | -0.57 |  |  |  |
|  |  |  |  |  |  |  |  |  |  |  |  |  |  |  | blue | ZCCHC11 | -0.57 |  |  |  |
|  |  |  |  |  |  |  |  |  |  |  |  |  |  |  | blue | GABRA3 | -0.57 |  |  |  |
|  |  |  |  |  |  |  |  |  |  |  |  |  |  |  | blue | ZMYND19 | -0.57 |  |  |  |
|  |  |  |  |  |  |  |  |  |  |  |  |  |  |  | blue | PLEKHH3 | -0.57 |  |  |  |
|  |  |  |  |  |  |  |  |  |  |  |  |  |  |  | blue | OPTN | -0.57 |  |  |  |
|  |  |  |  |  |  |  |  |  |  |  |  |  |  |  | blue | LAMA5 | -0.57 |  |  |  |
|  |  |  |  |  |  |  |  |  |  |  |  |  |  |  | blue | KIAA0895L | -0.57 |  |  |  |
|  |  |  |  |  |  |  |  |  |  |  |  |  |  |  | blue | KIF17 | -0.58 |  |  |  |
|  |  |  |  |  |  |  |  |  |  |  |  |  |  |  | blue | CHPF | -0.58 |  |  |  |
|  |  |  |  |  |  |  |  |  |  |  |  |  |  |  | blue | CPLX1 | -0.58 |  |  |  |
|  |  |  |  |  |  |  |  |  |  |  |  |  |  |  | blue | LINGO2 | -0.58 |  |  |  |
|  |  |  |  |  |  |  |  |  |  |  |  |  |  |  | blue | SPHK2 | -0.58 |  |  |  |
|  |  |  |  |  |  |  |  |  |  |  |  |  |  |  | blue | SFMBT2 | -0.58 |  |  |  |
|  |  |  |  |  |  |  |  |  |  |  |  |  |  |  | blue | ANKRD53 | -0.58 |  |  |  |
|  |  |  |  |  |  |  |  |  |  |  |  |  |  |  | blue | C2orf72 | -0.58 |  |  |  |
|  |  |  |  |  |  |  |  |  |  |  |  |  |  |  | blue | DRD3 | -0.58 |  |  |  |
|  |  |  |  |  |  |  |  |  |  |  |  |  |  |  | blue | PHGDH | -0.58 |  |  |  |
|  |  |  |  |  |  |  |  |  |  |  |  |  |  |  | blue | TLN2 | -0.58 |  |  |  |
|  |  |  |  |  |  |  |  |  |  |  |  |  |  |  | blue | PRSS53 | -0.58 |  |  |  |
|  |  |  |  |  |  |  |  |  |  |  |  |  |  |  | blue | C15orf57 | -0.58 |  |  |  |
|  |  |  |  |  |  |  |  |  |  |  |  |  |  |  | blue | ARMC5 | -0.58 |  |  |  |
|  |  |  |  |  |  |  |  |  |  |  |  |  |  |  | blue | RSPO4 | -0.58 |  |  |  |
|  |  |  |  |  |  |  |  |  |  |  |  |  |  |  | blue | PEX11G | -0.58 |  |  |  |
|  |  |  |  |  |  |  |  |  |  |  |  |  |  |  | blue | A4GALT | -0.58 |  |  |  |
|  |  |  |  |  |  |  |  |  |  |  |  |  |  |  | blue | ROM1 | -0.58 |  |  |  |
|  |  |  |  |  |  |  |  |  |  |  |  |  |  |  | blue | FAAP20 | -0.58 |  |  |  |
|  |  |  |  |  |  |  |  |  |  |  |  |  |  |  | blue | RAB3IL1 | -0.59 |  |  |  |
|  |  |  |  |  |  |  |  |  |  |  |  |  |  |  | blue | NAA60 | -0.59 |  |  |  |
|  |  |  |  |  |  |  |  |  |  |  |  |  |  |  | blue | FAM71E2 | -0.59 |  |  |  |
|  |  |  |  |  |  |  |  |  |  |  |  |  |  |  | blue | CEP95 | -0.59 |  |  |  |
|  |  |  |  |  |  |  |  |  |  |  |  |  |  |  | blue | ICA1 | -0.59 |  |  |  |
|  |  |  |  |  |  |  |  |  |  |  |  |  |  |  | blue | PIMREG | -0.59 |  |  |  |
|  |  |  |  |  |  |  |  |  |  |  |  |  |  |  | blue | ACACB | -0.59 |  |  |  |
|  |  |  |  |  |  |  |  |  |  |  |  |  |  |  | blue | EFEMP2 | -0.59 |  |  |  |
|  |  |  |  |  |  |  |  |  |  |  |  |  |  |  | blue | NAT6 | -0.59 |  |  |  |
|  |  |  |  |  |  |  |  |  |  |  |  |  |  |  | blue | CDK5RAP2 | -0.59 |  |  |  |
|  |  |  |  |  |  |  |  |  |  |  |  |  |  |  | blue | C7orf26 | -0.59 |  |  |  |
|  |  |  |  |  |  |  |  |  |  |  |  |  |  |  | blue | INPP5B | -0.59 |  |  |  |
|  |  |  |  |  |  |  |  |  |  |  |  |  |  |  | blue | PPP1R3G | -0.59 |  |  |  |
|  |  |  |  |  |  |  |  |  |  |  |  |  |  |  | blue | SHISA4 | -0.60 |  |  |  |
|  |  |  |  |  |  |  |  |  |  |  |  |  |  |  | blue | PTPA | -0.60 |  |  |  |
|  |  |  |  |  |  |  |  |  |  |  |  |  |  |  | blue | AC138028.1 | -0.60 |  |  |  |
|  |  |  |  |  |  |  |  |  |  |  |  |  |  |  | blue | LHX3 | -0.60 |  |  |  |
|  |  |  |  |  |  |  |  |  |  |  |  |  |  |  | blue | SPATA20 | -0.60 |  |  |  |
|  |  |  |  |  |  |  |  |  |  |  |  |  |  |  | blue | SMARCD3 | -0.60 |  |  |  |
|  |  |  |  |  |  |  |  |  |  |  |  |  |  |  | blue | MAP1A | -0.60 |  |  |  |
|  |  |  |  |  |  |  |  |  |  |  |  |  |  |  | blue | MTA1 | -0.60 |  |  |  |
|  |  |  |  |  |  |  |  |  |  |  |  |  |  |  | blue | HGH1 | -0.60 |  |  |  |
|  |  |  |  |  |  |  |  |  |  |  |  |  |  |  | blue | ITGB4 | -0.60 |  |  |  |
|  |  |  |  |  |  |  |  |  |  |  |  |  |  |  | blue | WIZ | -0.60 |  |  |  |
|  |  |  |  |  |  |  |  |  |  |  |  |  |  |  | blue | INPP5E | -0.60 |  |  |  |
|  |  |  |  |  |  |  |  |  |  |  |  |  |  |  | blue | PRAF2 | -0.60 |  |  |  |
|  |  |  |  |  |  |  |  |  |  |  |  |  |  |  | blue | DUS1L | -0.61 |  |  |  |
|  |  |  |  |  |  |  |  |  |  |  |  |  |  |  | blue | PIAS3 | -0.61 |  |  |  |
|  |  |  |  |  |  |  |  |  |  |  |  |  |  |  | blue | LZTS1 | -0.61 |  |  |  |
|  |  |  |  |  |  |  |  |  |  |  |  |  |  |  | blue | MICALL2 | -0.61 |  |  |  |
|  |  |  |  |  |  |  |  |  |  |  |  |  |  |  | blue | ALDH4A1 | -0.61 |  |  |  |
|  |  |  |  |  |  |  |  |  |  |  |  |  |  |  | blue | EXOC4 | -0.61 |  |  |  |
|  |  |  |  |  |  |  |  |  |  |  |  |  |  |  | blue | C1RL-AS1 | -0.61 |  |  |  |
|  |  |  |  |  |  |  |  |  |  |  |  |  |  |  | blue | SLC12A4 | -0.61 |  |  |  |
|  |  |  |  |  |  |  |  |  |  |  |  |  |  |  | blue | SSC5D | -0.61 |  |  |  |
|  |  |  |  |  |  |  |  |  |  |  |  |  |  |  | blue | DLL1 | -0.61 |  |  |  |
|  |  |  |  |  |  |  |  |  |  |  |  |  |  |  | blue | HNRNPM | -0.61 |  |  |  |
|  |  |  |  |  |  |  |  |  |  |  |  |  |  |  | blue | DCHS1 | -0.61 |  |  |  |
|  |  |  |  |  |  |  |  |  |  |  |  |  |  |  | blue | NTN5 | -0.61 |  |  |  |
|  |  |  |  |  |  |  |  |  |  |  |  |  |  |  | blue | GPR162 | -0.61 |  |  |  |
|  |  |  |  |  |  |  |  |  |  |  |  |  |  |  | blue | PHLDB3 | -0.61 |  |  |  |
|  |  |  |  |  |  |  |  |  |  |  |  |  |  |  | blue | HID1 | -0.61 |  |  |  |
|  |  |  |  |  |  |  |  |  |  |  |  |  |  |  | blue | CLIP2 | -0.61 |  |  |  |
|  |  |  |  |  |  |  |  |  |  |  |  |  |  |  | blue | ALDOB | -0.61 |  |  |  |
|  |  |  |  |  |  |  |  |  |  |  |  |  |  |  | blue | AHDC1 | -0.62 |  |  |  |
|  |  |  |  |  |  |  |  |  |  |  |  |  |  |  | blue | PDE9A | -0.62 |  |  |  |
|  |  |  |  |  |  |  |  |  |  |  |  |  |  |  | blue | SPATS2 | -0.62 |  |  |  |
|  |  |  |  |  |  |  |  |  |  |  |  |  |  |  | blue | TRIM28 | -0.62 |  |  |  |
|  |  |  |  |  |  |  |  |  |  |  |  |  |  |  | blue | CNTNAP1 | -0.62 |  |  |  |
|  |  |  |  |  |  |  |  |  |  |  |  |  |  |  | blue | ST5 | -0.62 |  |  |  |
|  |  |  |  |  |  |  |  |  |  |  |  |  |  |  | blue | CCDC84 | -0.62 |  |  |  |
|  |  |  |  |  |  |  |  |  |  |  |  |  |  |  | blue | HES4 | -0.62 |  |  |  |
|  |  |  |  |  |  |  |  |  |  |  |  |  |  |  | blue | SLC26A10 | -0.62 |  |  |  |
|  |  |  |  |  |  |  |  |  |  |  |  |  |  |  | blue | PACS2 | -0.62 |  |  |  |
|  |  |  |  |  |  |  |  |  |  |  |  |  |  |  | blue | DAAM2 | -0.62 |  |  |  |
|  |  |  |  |  |  |  |  |  |  |  |  |  |  |  | blue | CLSTN3 | -0.62 |  |  |  |
|  |  |  |  |  |  |  |  |  |  |  |  |  |  |  | blue | LTBP3 | -0.63 |  |  |  |
|  |  |  |  |  |  |  |  |  |  |  |  |  |  |  | blue | F9 | -0.63 |  |  |  |
|  |  |  |  |  |  |  |  |  |  |  |  |  |  |  | blue | C22orf34 | -0.63 |  |  |  |
|  |  |  |  |  |  |  |  |  |  |  |  |  |  |  | blue | HRK | -0.63 |  |  |  |
|  |  |  |  |  |  |  |  |  |  |  |  |  |  |  | blue | SH3GLB2 | -0.63 |  |  |  |
|  |  |  |  |  |  |  |  |  |  |  |  |  |  |  | blue | NPDC1 | -0.63 |  |  |  |
|  |  |  |  |  |  |  |  |  |  |  |  |  |  |  | blue | TRAF7 | -0.63 |  |  |  |
|  |  |  |  |  |  |  |  |  |  |  |  |  |  |  | blue | DOC2A | -0.63 |  |  |  |
|  |  |  |  |  |  |  |  |  |  |  |  |  |  |  | blue | CHST8 | -0.63 |  |  |  |
|  |  |  |  |  |  |  |  |  |  |  |  |  |  |  | blue | MYEF2 | -0.63 |  |  |  |
|  |  |  |  |  |  |  |  |  |  |  |  |  |  |  | blue | POFUT2 | -0.64 |  |  |  |
|  |  |  |  |  |  |  |  |  |  |  |  |  |  |  | blue | KALRN | -0.64 |  |  |  |
|  |  |  |  |  |  |  |  |  |  |  |  |  |  |  | blue | MRPL2 | -0.64 |  |  |  |
|  |  |  |  |  |  |  |  |  |  |  |  |  |  |  | blue | ZNF83 | -0.64 |  |  |  |
|  |  |  |  |  |  |  |  |  |  |  |  |  |  |  | blue | MYLPF | -0.64 |  |  |  |
|  |  |  |  |  |  |  |  |  |  |  |  |  |  |  | blue | SLC11A2 | -0.64 |  |  |  |
|  |  |  |  |  |  |  |  |  |  |  |  |  |  |  | blue | CDK16 | -0.64 |  |  |  |
|  |  |  |  |  |  |  |  |  |  |  |  |  |  |  | blue | TRAP1 | -0.64 |  |  |  |
|  |  |  |  |  |  |  |  |  |  |  |  |  |  |  | blue | GRIA3 | -0.64 |  |  |  |
|  |  |  |  |  |  |  |  |  |  |  |  |  |  |  | blue | EXOSC10 | -0.64 |  |  |  |
|  |  |  |  |  |  |  |  |  |  |  |  |  |  |  | blue | GDPD5 | -0.64 |  |  |  |
|  |  |  |  |  |  |  |  |  |  |  |  |  |  |  | blue | PNKD | -0.64 |  |  |  |
|  |  |  |  |  |  |  |  |  |  |  |  |  |  |  | blue | DNMT3B | -0.65 |  |  |  |
|  |  |  |  |  |  |  |  |  |  |  |  |  |  |  | blue | ELAVL3 | -0.65 |  |  |  |
|  |  |  |  |  |  |  |  |  |  |  |  |  |  |  | blue | CLTB | -0.65 |  |  |  |
|  |  |  |  |  |  |  |  |  |  |  |  |  |  |  | blue | NOD1 | -0.65 |  |  |  |
|  |  |  |  |  |  |  |  |  |  |  |  |  |  |  | blue | SCNN1D | -0.65 |  |  |  |
|  |  |  |  |  |  |  |  |  |  |  |  |  |  |  | blue | KAT2A | -0.65 |  |  |  |
|  |  |  |  |  |  |  |  |  |  |  |  |  |  |  | blue | MTG1 | -0.65 |  |  |  |
|  |  |  |  |  |  |  |  |  |  |  |  |  |  |  | blue | MFGE8 | -0.65 |  |  |  |
|  |  |  |  |  |  |  |  |  |  |  |  |  |  |  | blue | SRL | -0.65 |  |  |  |
|  |  |  |  |  |  |  |  |  |  |  |  |  |  |  | blue | ADRA2C | -0.65 |  |  |  |
|  |  |  |  |  |  |  |  |  |  |  |  |  |  |  | blue | NDUFB7 | -0.65 |  |  |  |
|  |  |  |  |  |  |  |  |  |  |  |  |  |  |  | blue | WDR90 | -0.65 |  |  |  |
|  |  |  |  |  |  |  |  |  |  |  |  |  |  |  | blue | SMTN | -0.65 |  |  |  |
|  |  |  |  |  |  |  |  |  |  |  |  |  |  |  | blue | ERVW-1 | -0.65 |  |  |  |
|  |  |  |  |  |  |  |  |  |  |  |  |  |  |  | blue | NEK8 | -0.65 |  |  |  |
|  |  |  |  |  |  |  |  |  |  |  |  |  |  |  | blue | CNTROB | -0.66 |  |  |  |
|  |  |  |  |  |  |  |  |  |  |  |  |  |  |  | blue | TJAP1 | -0.66 |  |  |  |
|  |  |  |  |  |  |  |  |  |  |  |  |  |  |  | blue | NUCB2 | -0.66 |  |  |  |
|  |  |  |  |  |  |  |  |  |  |  |  |  |  |  | blue | ZNF580 | -0.66 |  |  |  |
|  |  |  |  |  |  |  |  |  |  |  |  |  |  |  | blue | SNX21 | -0.66 |  |  |  |
|  |  |  |  |  |  |  |  |  |  |  |  |  |  |  | blue | FOXRED1 | -0.66 |  |  |  |
|  |  |  |  |  |  |  |  |  |  |  |  |  |  |  | blue | AKT1S1 | -0.66 |  |  |  |
|  |  |  |  |  |  |  |  |  |  |  |  |  |  |  | blue | MTFP1 | -0.66 |  |  |  |
|  |  |  |  |  |  |  |  |  |  |  |  |  |  |  | blue | ADAM15 | -0.66 |  |  |  |
|  |  |  |  |  |  |  |  |  |  |  |  |  |  |  | blue | INO80B | -0.67 |  |  |  |
|  |  |  |  |  |  |  |  |  |  |  |  |  |  |  | blue | FOXL1 | -0.67 |  |  |  |
|  |  |  |  |  |  |  |  |  |  |  |  |  |  |  | blue | MICA | -0.67 |  |  |  |
|  |  |  |  |  |  |  |  |  |  |  |  |  |  |  | blue | CALU | -0.67 |  |  |  |
|  |  |  |  |  |  |  |  |  |  |  |  |  |  |  | blue | SNX33 | -0.67 |  |  |  |
|  |  |  |  |  |  |  |  |  |  |  |  |  |  |  | blue | NKTR | -0.67 |  |  |  |
|  |  |  |  |  |  |  |  |  |  |  |  |  |  |  | blue | RCC2 | -0.67 |  |  |  |
|  |  |  |  |  |  |  |  |  |  |  |  |  |  |  | blue | ISYNA1 | -0.67 |  |  |  |
|  |  |  |  |  |  |  |  |  |  |  |  |  |  |  | blue | FBXO44 | -0.67 |  |  |  |
|  |  |  |  |  |  |  |  |  |  |  |  |  |  |  | blue | CCDC85B | -0.67 |  |  |  |
|  |  |  |  |  |  |  |  |  |  |  |  |  |  |  | blue | ETNK2 | -0.67 |  |  |  |
|  |  |  |  |  |  |  |  |  |  |  |  |  |  |  | blue | METTL26 | -0.68 |  |  |  |
|  |  |  |  |  |  |  |  |  |  |  |  |  |  |  | blue | SF3B3 | -0.68 |  |  |  |
|  |  |  |  |  |  |  |  |  |  |  |  |  |  |  | blue | ZNF444 | -0.68 |  |  |  |
|  |  |  |  |  |  |  |  |  |  |  |  |  |  |  | blue | ACADVL | -0.68 |  |  |  |
|  |  |  |  |  |  |  |  |  |  |  |  |  |  |  | blue | CDK10 | -0.68 |  |  |  |
|  |  |  |  |  |  |  |  |  |  |  |  |  |  |  | blue | ELAC2 | -0.68 |  |  |  |
|  |  |  |  |  |  |  |  |  |  |  |  |  |  |  | blue | SEMA6C | -0.68 |  |  |  |
|  |  |  |  |  |  |  |  |  |  |  |  |  |  |  | blue | MYL6B | -0.68 |  |  |  |
|  |  |  |  |  |  |  |  |  |  |  |  |  |  |  | blue | PLXNB1 | -0.68 |  |  |  |
|  |  |  |  |  |  |  |  |  |  |  |  |  |  |  | blue | SNRNP70 | -0.68 |  |  |  |
|  |  |  |  |  |  |  |  |  |  |  |  |  |  |  | blue | NAT14 | -0.69 |  |  |  |
|  |  |  |  |  |  |  |  |  |  |  |  |  |  |  | blue | DDX27 | -0.69 |  |  |  |
|  |  |  |  |  |  |  |  |  |  |  |  |  |  |  | blue | PTMS | -0.69 |  |  |  |
|  |  |  |  |  |  |  |  |  |  |  |  |  |  |  | blue | SOD3 | -0.69 |  |  |  |
|  |  |  |  |  |  |  |  |  |  |  |  |  |  |  | blue | PSG6 | -0.69 |  |  |  |
|  |  |  |  |  |  |  |  |  |  |  |  |  |  |  | blue | MTSS1L | -0.69 |  |  |  |
|  |  |  |  |  |  |  |  |  |  |  |  |  |  |  | blue | EVI5L | -0.69 |  |  |  |
|  |  |  |  |  |  |  |  |  |  |  |  |  |  |  | blue | PGAP3 | -0.69 |  |  |  |
|  |  |  |  |  |  |  |  |  |  |  |  |  |  |  | blue | SORBS3 | -0.69 |  |  |  |
|  |  |  |  |  |  |  |  |  |  |  |  |  |  |  | blue | TRO | -0.69 |  |  |  |
|  |  |  |  |  |  |  |  |  |  |  |  |  |  |  | blue | ADRM1 | -0.69 |  |  |  |
|  |  |  |  |  |  |  |  |  |  |  |  |  |  |  | blue | GBF1 | -0.69 |  |  |  |
|  |  |  |  |  |  |  |  |  |  |  |  |  |  |  | blue | GH1 | -0.69 |  |  |  |
|  |  |  |  |  |  |  |  |  |  |  |  |  |  |  | blue | LENEP | -0.70 |  |  |  |
|  |  |  |  |  |  |  |  |  |  |  |  |  |  |  | blue | CTIF | -0.70 |  |  |  |
|  |  |  |  |  |  |  |  |  |  |  |  |  |  |  | blue | MLXIPL | -0.70 |  |  |  |
|  |  |  |  |  |  |  |  |  |  |  |  |  |  |  | blue | BCL2L12 | -0.70 |  |  |  |
|  |  |  |  |  |  |  |  |  |  |  |  |  |  |  | blue | RAPGEF3 | -0.70 |  |  |  |
|  |  |  |  |  |  |  |  |  |  |  |  |  |  |  | blue | ARFGAP1 | -0.70 |  |  |  |
|  |  |  |  |  |  |  |  |  |  |  |  |  |  |  | blue | SDCCAG3 | -0.70 |  |  |  |
|  |  |  |  |  |  |  |  |  |  |  |  |  |  |  | blue | MAPK8IP2 | -0.70 |  |  |  |
|  |  |  |  |  |  |  |  |  |  |  |  |  |  |  | blue | SCYL1 | -0.71 |  |  |  |
|  |  |  |  |  |  |  |  |  |  |  |  |  |  |  | blue | SAMD4B | -0.71 |  |  |  |
|  |  |  |  |  |  |  |  |  |  |  |  |  |  |  | blue | TXN2 | -0.71 |  |  |  |
|  |  |  |  |  |  |  |  |  |  |  |  |  |  |  | blue | SWI5 | -0.71 |  |  |  |
|  |  |  |  |  |  |  |  |  |  |  |  |  |  |  | blue | PHF1 | -0.71 |  |  |  |
|  |  |  |  |  |  |  |  |  |  |  |  |  |  |  | blue | PELP1 | -0.71 |  |  |  |
|  |  |  |  |  |  |  |  |  |  |  |  |  |  |  | blue | APBB1 | -0.71 |  |  |  |
|  |  |  |  |  |  |  |  |  |  |  |  |  |  |  | blue | D2HGDH | -0.71 |  |  |  |
|  |  |  |  |  |  |  |  |  |  |  |  |  |  |  | blue | ARL6IP4 | -0.71 |  |  |  |
|  |  |  |  |  |  |  |  |  |  |  |  |  |  |  | blue | MAPK11 | -0.71 |  |  |  |
|  |  |  |  |  |  |  |  |  |  |  |  |  |  |  | blue | SGSM2 | -0.72 |  |  |  |
|  |  |  |  |  |  |  |  |  |  |  |  |  |  |  | blue | HIP1R | -0.72 |  |  |  |
|  |  |  |  |  |  |  |  |  |  |  |  |  |  |  | blue | RPL9 | -0.72 |  |  |  |
|  |  |  |  |  |  |  |  |  |  |  |  |  |  |  | blue | HP1BP3 | -0.72 |  |  |  |
|  |  |  |  |  |  |  |  |  |  |  |  |  |  |  | blue | SERGEF | -0.72 |  |  |  |
|  |  |  |  |  |  |  |  |  |  |  |  |  |  |  | blue | KDELR1 | -0.72 |  |  |  |
|  |  |  |  |  |  |  |  |  |  |  |  |  |  |  | blue | NFATC4 | -0.72 |  |  |  |
|  |  |  |  |  |  |  |  |  |  |  |  |  |  |  | blue | LUC7L | -0.72 |  |  |  |
|  |  |  |  |  |  |  |  |  |  |  |  |  |  |  | blue | MAST4 | -0.72 |  |  |  |
|  |  |  |  |  |  |  |  |  |  |  |  |  |  |  | blue | PDAP1 | -0.72 |  |  |  |
|  |  |  |  |  |  |  |  |  |  |  |  |  |  |  | blue | PTDSS2 | -0.72 |  |  |  |
|  |  |  |  |  |  |  |  |  |  |  |  |  |  |  | blue | NLGN2 | -0.72 |  |  |  |
|  |  |  |  |  |  |  |  |  |  |  |  |  |  |  | blue | DLG4 | -0.72 |  |  |  |
|  |  |  |  |  |  |  |  |  |  |  |  |  |  |  | blue | METRN | -0.73 |  |  |  |
|  |  |  |  |  |  |  |  |  |  |  |  |  |  |  | blue | MCRIP1 | -0.74 |  |  |  |
|  |  |  |  |  |  |  |  |  |  |  |  |  |  |  | blue | DFFA | -0.74 |  |  |  |
|  |  |  |  |  |  |  |  |  |  |  |  |  |  |  | blue | TRPT1 | -0.74 |  |  |  |
|  |  |  |  |  |  |  |  |  |  |  |  |  |  |  | blue | ZNRF1 | -0.75 |  |  |  |
|  |  |  |  |  |  |  |  |  |  |  |  |  |  |  | blue | MBD3 | -0.75 |  |  |  |
|  |  |  |  |  |  |  |  |  |  |  |  |  |  |  | blue | ZBTB17 | -0.75 |  |  |  |
|  |  |  |  |  |  |  |  |  |  |  |  |  |  |  | blue | PTOV1 | -0.75 |  |  |  |
|  |  |  |  |  |  |  |  |  |  |  |  |  |  |  | blue | SLC25A29 | -0.76 |  |  |  |
|  |  |  |  |  |  |  |  |  |  |  |  |  |  |  | blue | ZNF579 | -0.76 |  |  |  |
|  |  |  |  |  |  |  |  |  |  |  |  |  |  |  | blue | CIZ1 | -0.76 |  |  |  |
|  |  |  |  |  |  |  |  |  |  |  |  |  |  |  | blue | SLC22A17 | -0.76 |  |  |  |
|  |  |  |  |  |  |  |  |  |  |  |  |  |  |  | blue | CCDC25 | -0.76 |  |  |  |
|  |  |  |  |  |  |  |  |  |  |  |  |  |  |  | blue | TCEA2 | -0.76 |  |  |  |
|  |  |  |  |  |  |  |  |  |  |  |  |  |  |  | blue | HSF4 | -0.77 |  |  |  |
|  |  |  |  |  |  |  |  |  |  |  |  |  |  |  | blue | LRP3 | -0.77 |  |  |  |
|  |  |  |  |  |  |  |  |  |  |  |  |  |  |  | blue | SUPT5H | -0.78 |  |  |  |
|  |  |  |  |  |  |  |  |  |  |  |  |  |  |  | blue | CPSF4 | -0.78 |  |  |  |
|  |  |  |  |  |  |  |  |  |  |  |  |  |  |  | blue | STK25 | -0.78 |  |  |  |
|  |  |  |  |  |  |  |  |  |  |  |  |  |  |  | blue | EXT2 | -0.78 |  |  |  |
|  |  |  |  |  |  |  |  |  |  |  |  |  |  |  | blue | GTF3C1 | -0.78 |  |  |  |
|  |  |  |  |  |  |  |  |  |  |  |  |  |  |  | blue | NFIC | -0.80 |  |  |  |
|  |  |  |  |  |  |  |  |  |  |  |  |  |  |  | blue | SRRM2 | -0.80 |  |  |  |
|  |  |  |  |  |  |  |  |  |  |  |  |  |  |  | blue | UBE2Z | -0.80 |  |  |  |
|  |  |  |  |  |  |  |  |  |  |  |  |  |  |  | blue | POLD2 | -0.82 |  |  |  |
|  |  |  |  |  |  |  |  |  |  |  |  |  |  |  | blue | RABL6 | -0.84 |  |  |  |
|  |  |  |  |  |  |  |  |  |  |  |  |  |  |  | blue | SPTAN1 | -0.84 |  |  |  |
|  |  |  |  |  |  |  |  |  |  |  |  |  |  |  | blue | RAD23A | -0.88 |  |  |  |
|  |  |  |  |  |  |  |  |  |  |  |  |  |  |  | blue | FAM3A | -0.88 |  |  |  |
|  |  |  |  |  |  |  |  |  |  |  |  |  |  |  | blue | KHSRP | -0.89 |  |  |  |

**Table S5:** Gene ontology Biological Processes enrichment for each of the modules significantly associated with the percentage of macrophages.

|  | | |  |  |
| --- | --- | --- | --- | --- |
| **Module** | **Gene ontology biological process ID** | **Description** | **p.adjust** | **Count** |
| BLUE | GO:0006888 | ER to Golgi vesicle-mediated transport | 2.75549E-05 | 27 |
| BLUE | GO:0048193 | Golgi vesicle transport | 0.000112751 | 36 |
| BLUE | GO:0006900 | membrane budding | 0.011648268 | 16 |
| BLUE | GO:0006901 | vesicle coating | 0.01189045 | 12 |
| BLUE | GO:0048199 | vesicle targeting; to; from or within Golgi | 0.01237261 | 12 |
| BLUE | GO:0006903 | vesicle targeting | 0.01237261 | 13 |
| BLUE | GO:0006417 | regulation of translation | 0.01237261 | 32 |
| BLUE | GO:0034248 | regulation of cellular amide metabolic process | 0.01237261 | 34 |
| BLUE | GO:0016050 | vesicle organization | 0.015058466 | 31 |
| BLUE | GO:0048207 | vesicle targeting; rough ER to cis-Golgi | 0.016973053 | 11 |
| BLUE | GO:0048208 | COPII vesicle coating | 0.016973053 | 11 |
| BLUE | GO:0007034 | vacuolar transport | 0.018153659 | 27 |
| BLUE | GO:0007009 | plasma membrane organization | 0.020066317 | 26 |
| BLUE | GO:0006397 | mRNA processing | 0.020066317 | 39 |
| BLUE | GO:0090114 | COPII-coated vesicle budding | 0.022745069 | 11 |
| BLUE | GO:1903311 | regulation of mRNA metabolic process | 0.022745069 | 16 |
| BLUE | GO:0010608 | posttranscriptional regulation of gene expression | 0.024473352 | 38 |
| BLUE | GO:0072659 | protein localization to plasma membrane | 0.031866047 | 21 |
| BLUE | GO:0048313 | Golgi inheritance | 0.045221961 | 5 |
| BLUE | GO:0008380 | RNA splicing | 0.045221961 | 33 |
| Green Yellow | GO:0030198 | extracellular matrix organization | 6.76576E-25 | 40 |
| Green Yellow | GO:0043062 | extracellular structure organization | 6.76576E-25 | 40 |
| Green Yellow | GO:0032963 | collagen metabolic process | 9.83947E-13 | 18 |
| Green Yellow | GO:0001501 | skeletal system development | 9.83947E-13 | 33 |
| Green Yellow | GO:0030574 | collagen catabolic process | 1.55077E-12 | 15 |
| Green Yellow | GO:0044259 | multicellular organismal macromolecule metabolic process | 1.55077E-12 | 18 |
| Green Yellow | GO:0044243 | multicellular organism catabolic process | 6.49535E-12 | 15 |
| Green Yellow | GO:0030199 | collagen fibril organization | 1.06448E-11 | 12 |
| Green Yellow | GO:0044236 | multicellular organism metabolic process | 1.06448E-11 | 18 |
| Green Yellow | GO:0048514 | blood vessel morphogenesis | 1.30459E-10 | 30 |
| Green Yellow | GO:0001525 | angiogenesis | 2.71393E-09 | 26 |
| Green Yellow | GO:0001503 | ossification | 5.86586E-09 | 24 |
| Green Yellow | GO:0061448 | connective tissue development | 1.98241E-08 | 19 |
| Green Yellow | GO:0051216 | cartilage development | 1.91436E-07 | 16 |
| Green Yellow | GO:1901342 | regulation of vasculature development | 1.02001E-06 | 17 |
| Green Yellow | GO:0045765 | regulation of angiogenesis | 1.57184E-06 | 16 |
| Green Yellow | GO:0010975 | regulation of neuron projection development | 4.75268E-06 | 21 |
| Green Yellow | GO:0001822 | kidney development | 5.19171E-06 | 17 |
| Green Yellow | GO:0060348 | bone development | 6.14194E-06 | 14 |
| Green Yellow | GO:0031589 | cell-substrate adhesion | 6.14194E-06 | 18 |
| Green Yellow | GO:0050673 | epithelial cell proliferation | 6.14194E-06 | 19 |
| Green Yellow | GO:0060485 | mesenchyme development | 7.51611E-06 | 16 |
| Green Yellow | GO:0072001 | renal system development | 8.68187E-06 | 17 |
| Green Yellow | GO:2000027 | regulation of organ morphogenesis | 8.80296E-06 | 16 |
| Green Yellow | GO:0001655 | urogenital system development | 8.80296E-06 | 18 |
| Green Yellow | GO:0061564 | axon development | 1.6389E-05 | 21 |
| Green Yellow | GO:0001649 | osteoblast differentiation | 2.47996E-05 | 14 |
| Green Yellow | GO:0010810 | regulation of cell-substrate adhesion | 2.81299E-05 | 13 |
| Green Yellow | GO:0007423 | sensory organ development | 2.81299E-05 | 22 |
| Green Yellow | GO:0071230 | cellular response to amino acid stimulus | 3.01168E-05 | 8 |
| Green Yellow | GO:0090288 | negative regulation of cellular response to growth factor stimulus | 3.01168E-05 | 11 |
| Green Yellow | GO:0030278 | regulation of ossification | 3.01168E-05 | 13 |
| Green Yellow | GO:0048705 | skeletal system morphogenesis | 3.01168E-05 | 14 |
| Green Yellow | GO:0090596 | sensory organ morphogenesis | 3.64329E-05 | 15 |
| Green Yellow | GO:0048762 | mesenchymal cell differentiation | 4.16493E-05 | 13 |
| Green Yellow | GO:0006026 | aminoglycan catabolic process | 0.000125584 | 8 |
| Green Yellow | GO:0042476 | odontogenesis | 0.000125584 | 10 |
| Green Yellow | GO:0002062 | chondrocyte differentiation | 0.000195196 | 9 |
| Green Yellow | GO:0048562 | embryonic organ morphogenesis | 0.000195196 | 15 |
| Green Yellow | GO:0040013 | negative regulation of locomotion | 0.000207557 | 14 |
| Green Yellow | GO:0051271 | negative regulation of cellular component movement | 0.000212227 | 14 |
| Green Yellow | GO:0007409 | axonogenesis | 0.000224315 | 18 |
| Green Yellow | GO:2000146 | negative regulation of cell motility | 0.000262941 | 13 |
| Green Yellow | GO:0048568 | embryonic organ development | 0.000262941 | 18 |
| Green Yellow | GO:0033002 | muscle cell proliferation | 0.000302432 | 11 |
| Green Yellow | GO:0097435 | fibril organization | 0.000303788 | 5 |
| Green Yellow | GO:0090287 | regulation of cellular response to growth factor stimulus | 0.000303788 | 13 |
| Green Yellow | GO:0043583 | ear development | 0.000438088 | 12 |
| Green Yellow | GO:0070206 | protein trimerization | 0.000465436 | 6 |
| Green Yellow | GO:1904018 | positive regulation of vasculature development | 0.000465436 | 10 |
| Green Yellow | GO:0060828 | regulation of canonical Wnt signaling pathway | 0.000483454 | 13 |
| Green Yellow | GO:0006022 | aminoglycan metabolic process | 0.000498699 | 11 |
| Green Yellow | GO:0006027 | glycosaminoglycan catabolic process | 0.000566913 | 7 |
| Green Yellow | GO:0030336 | negative regulation of cell migration | 0.000621489 | 12 |
| Green Yellow | GO:0050678 | regulation of epithelial cell proliferation | 0.000621489 | 14 |
| Green Yellow | GO:1905330 | regulation of morphogenesis of an epithelium | 0.000633048 | 11 |
| Green Yellow | GO:0022617 | extracellular matrix disassembly | 0.00066987 | 8 |
| Green Yellow | GO:0045995 | regulation of embryonic development | 0.00066987 | 9 |
| Green Yellow | GO:0048667 | cell morphogenesis involved in neuron differentiation | 0.00066987 | 19 |
| Green Yellow | GO:0008589 | regulation of smoothened signaling pathway | 0.000679552 | 7 |
| Green Yellow | GO:0007224 | smoothened signaling pathway | 0.00082871 | 9 |
| Green Yellow | GO:0022612 | gland morphogenesis | 0.000859073 | 9 |
| Green Yellow | GO:0045766 | positive regulation of angiogenesis | 0.000859073 | 9 |
| Green Yellow | GO:0043200 | response to amino acid | 0.000911729 | 8 |
| Green Yellow | GO:0032102 | negative regulation of response to external stimulus | 0.000911729 | 13 |
| Green Yellow | GO:0030500 | regulation of bone mineralization | 0.000912073 | 7 |
| Green Yellow | GO:0010717 | regulation of epithelial to mesenchymal transition | 0.001088332 | 7 |
| Green Yellow | GO:0071229 | cellular response to acid chemical | 0.001279136 | 10 |
| Green Yellow | GO:0030111 | regulation of Wnt signaling pathway | 0.001310511 | 14 |
| Green Yellow | GO:0030282 | bone mineralization | 0.001322595 | 8 |
| Green Yellow | GO:0031214 | biomineral tissue development | 0.001427283 | 9 |
| Green Yellow | GO:0030203 | glycosaminoglycan metabolic process | 0.001427283 | 10 |
| Green Yellow | GO:0070167 | regulation of biomineral tissue development | 0.001714297 | 7 |
| Green Yellow | GO:0071560 | cellular response to transforming growth factor beta stimulus | 0.002239475 | 11 |
| Green Yellow | GO:0048839 | inner ear development | 0.002268042 | 10 |
| Green Yellow | GO:0048660 | regulation of smooth muscle cell proliferation | 0.002281326 | 8 |
| Green Yellow | GO:0071559 | response to transforming growth factor beta | 0.002349695 | 11 |
| Green Yellow | GO:0045667 | regulation of osteoblast differentiation | 0.002375108 | 8 |
| Green Yellow | GO:0001837 | epithelial to mesenchymal transition | 0.002472515 | 8 |
| Green Yellow | GO:0048659 | smooth muscle cell proliferation | 0.002472515 | 8 |
| Green Yellow | GO:0060349 | bone morphogenesis | 0.002521582 | 7 |
| Green Yellow | GO:1901136 | carbohydrate derivative catabolic process | 0.002521582 | 10 |
| Green Yellow | GO:0010470 | regulation of gastrulation | 0.002617676 | 5 |
| Green Yellow | GO:0006024 | glycosaminoglycan biosynthetic process | 0.002617676 | 8 |
| Green Yellow | GO:0042471 | ear morphogenesis | 0.002617676 | 8 |
| Green Yellow | GO:0060070 | canonical Wnt signaling pathway | 0.002617676 | 13 |
| Green Yellow | GO:0006029 | proteoglycan metabolic process | 0.002725421 | 7 |
| Green Yellow | GO:0006023 | aminoglycan biosynthetic process | 0.002725421 | 8 |
| Green Yellow | GO:0007411 | axon guidance | 0.002741067 | 11 |
| Green Yellow | GO:0097485 | neuron projection guidance | 0.002825324 | 11 |
| Green Yellow | GO:0061053 | somite development | 0.002839637 | 7 |
| Green Yellow | GO:0035924 | cellular response to vascular endothelial growth factor stimulus | 0.003153452 | 5 |
| Green Yellow | GO:0030177 | positive regulation of Wnt signaling pathway | 0.003187163 | 9 |
| Green Yellow | GO:0051604 | protein maturation | 0.003187163 | 12 |
| Green Yellow | GO:0090263 | positive regulation of canonical Wnt signaling pathway | 0.003428075 | 8 |
| Green Yellow | GO:0043542 | endothelial cell migration | 0.003626315 | 9 |
| Green Yellow | GO:0050679 | positive regulation of epithelial cell proliferation | 0.003760989 | 9 |
| Green Yellow | GO:0007178 | transmembrane receptor protein serine/threonine kinase signaling pathway | 0.003760989 | 13 |
| Green Yellow | GO:0044273 | sulfur compound catabolic process | 0.003770377 | 5 |
| Green Yellow | GO:2000147 | positive regulation of cell motility | 0.004239642 | 15 |
| Green Yellow | GO:0048706 | embryonic skeletal system development | 0.004310271 | 8 |
| Green Yellow | GO:0007160 | cell-matrix adhesion | 0.004365442 | 10 |
| Green Yellow | GO:0016485 | protein processing | 0.004636619 | 11 |
| Green Yellow | GO:0031099 | regeneration | 0.004807743 | 9 |
| Green Yellow | GO:0048608 | reproductive structure development | 0.004807743 | 15 |
| Green Yellow | GO:0048732 | gland development | 0.004807743 | 15 |
| Green Yellow | GO:0051272 | positive regulation of cellular component movement | 0.005143239 | 15 |
| Green Yellow | GO:0061458 | reproductive system development | 0.005215111 | 15 |
| Green Yellow | GO:0040017 | positive regulation of locomotion | 0.005230885 | 15 |
| Green Yellow | GO:0090090 | negative regulation of canonical Wnt signaling pathway | 0.005238643 | 9 |
| Green Yellow | GO:0044706 | multi-multicellular organism process | 0.005395909 | 10 |
| Green Yellow | GO:0060560 | developmental growth involved in morphogenesis | 0.006114041 | 10 |
| Green Yellow | GO:0030850 | prostate gland development | 0.006119927 | 5 |
| Green Yellow | GO:0016525 | negative regulation of angiogenesis | 0.006352174 | 6 |
| Green Yellow | GO:0001764 | neuron migration | 0.006352174 | 8 |
| Green Yellow | GO:0016055 | Wnt signaling pathway | 0.006352174 | 16 |
| Green Yellow | GO:0198738 | cell-cell signaling by wnt | 0.006352174 | 16 |
| Green Yellow | GO:0001823 | mesonephros development | 0.006359149 | 7 |
| Green Yellow | GO:0010811 | positive regulation of cell-substrate adhesion | 0.006359149 | 7 |
| Green Yellow | GO:2000181 | negative regulation of blood vessel morphogenesis | 0.006888253 | 6 |
| Green Yellow | GO:0030509 | BMP signaling pathway | 0.006888253 | 8 |
| Green Yellow | GO:0001558 | regulation of cell growth | 0.006888253 | 14 |
| Green Yellow | GO:0030335 | positive regulation of cell migration | 0.007589879 | 14 |
| Green Yellow | GO:0016049 | cell growth | 0.007589879 | 16 |
| Green Yellow | GO:0014033 | neural crest cell differentiation | 0.007659112 | 6 |
| Green Yellow | GO:0051962 | positive regulation of nervous system development | 0.007659112 | 15 |
| Green Yellow | GO:0007369 | gastrulation | 0.007712881 | 9 |
| Green Yellow | GO:0048592 | eye morphogenesis | 0.007776592 | 8 |
| Green Yellow | GO:0060840 | artery development | 0.007817785 | 6 |
| Green Yellow | GO:0007565 | female pregnancy | 0.007861281 | 9 |
| Green Yellow | GO:0010812 | negative regulation of cell-substrate adhesion | 0.008219996 | 5 |
| Green Yellow | GO:1903034 | regulation of response to wounding | 0.008316985 | 8 |
| Green Yellow | GO:0060688 | regulation of morphogenesis of a branching structure | 0.008857311 | 5 |
| Green Yellow | GO:0007596 | blood coagulation | 0.008857311 | 13 |
| Green Yellow | GO:0061138 | morphogenesis of a branching epithelium | 0.008897009 | 9 |
| Green Yellow | GO:0007507 | heart development | 0.008897009 | 16 |
| Green Yellow | GO:0070613 | regulation of protein processing | 0.008958983 | 6 |
| Green Yellow | GO:1901343 | negative regulation of vasculature development | 0.008958983 | 6 |
| Green Yellow | GO:0001101 | response to acid chemical | 0.008958983 | 11 |
| Green Yellow | GO:2000242 | negative regulation of reproductive process | 0.00903189 | 5 |
| Green Yellow | GO:0045778 | positive regulation of ossification | 0.00903189 | 6 |
| Green Yellow | GO:1903317 | regulation of protein maturation | 0.00903189 | 6 |
| Green Yellow | GO:0071772 | response to BMP | 0.00903189 | 8 |
| Green Yellow | GO:0071773 | cellular response to BMP stimulus | 0.00903189 | 8 |
| Green Yellow | GO:0050817 | coagulation | 0.00903189 | 13 |
| Green Yellow | GO:0007599 | hemostasis | 0.009150761 | 13 |
| Green Yellow | GO:0010594 | regulation of endothelial cell migration | 0.009171818 | 7 |
| Green Yellow | GO:0010721 | negative regulation of cell development | 0.010359464 | 11 |
| Green Yellow | GO:0001704 | formation of primary germ layer | 0.011097601 | 7 |
| Green Yellow | GO:0035567 | non-canonical Wnt signaling pathway | 0.011756902 | 8 |
| Green Yellow | GO:0071417 | cellular response to organonitrogen compound | 0.011756902 | 15 |
| Green Yellow | GO:0001763 | morphogenesis of a branching structure | 0.012291264 | 9 |
| Green Yellow | GO:0001667 | ameboidal-type cell migration | 0.012291264 | 12 |
| Green Yellow | GO:0045785 | positive regulation of cell adhesion | 0.012852416 | 13 |
| Green Yellow | GO:2000241 | regulation of reproductive process | 0.012872902 | 7 |
| Green Yellow | GO:0001656 | metanephros development | 0.013584744 | 6 |
| Green Yellow | GO:0030178 | negative regulation of Wnt signaling pathway | 0.013587171 | 9 |
| Green Yellow | GO:0003002 | regionalization | 0.01365076 | 12 |
| Green Yellow | GO:0032835 | glomerulus development | 0.013964458 | 5 |
| Green Yellow | GO:0061041 | regulation of wound healing | 0.014316915 | 7 |
| Green Yellow | GO:0009952 | anterior/posterior pattern specification | 0.014316915 | 9 |
| Green Yellow | GO:0042733 | embryonic digit morphogenesis | 0.014740393 | 5 |
| Green Yellow | GO:0042472 | inner ear morphogenesis | 0.015008964 | 6 |
| Green Yellow | GO:0030326 | embryonic limb morphogenesis | 0.015008964 | 7 |
| Green Yellow | GO:0035113 | embryonic appendage morphogenesis | 0.015008964 | 7 |
| Green Yellow | GO:0035282 | segmentation | 0.015799468 | 6 |
| Green Yellow | GO:0009100 | glycoprotein metabolic process | 0.01697614 | 13 |
| Green Yellow | GO:0042306 | regulation of protein import into nucleus | 0.017127292 | 8 |
| Green Yellow | GO:0050727 | regulation of inflammatory response | 0.017500004 | 11 |
| Green Yellow | GO:0001657 | ureteric bud development | 0.018799306 | 6 |
| Green Yellow | GO:0048565 | digestive tract development | 0.018799306 | 7 |
| Green Yellow | GO:0030324 | lung development | 0.018799306 | 8 |
| Green Yellow | GO:1904589 | regulation of protein import | 0.018799306 | 8 |
| Green Yellow | GO:0001756 | somitogenesis | 0.019227341 | 5 |
| Green Yellow | GO:1900180 | regulation of protein localization to nucleus | 0.01928499 | 9 |
| Green Yellow | GO:0045185 | maintenance of protein location | 0.0193512 | 6 |
| Green Yellow | GO:0072163 | mesonephric epithelium development | 0.0193512 | 6 |
| Green Yellow | GO:0072164 | mesonephric tubule development | 0.0193512 | 6 |
| Green Yellow | GO:0070482 | response to oxygen levels | 0.019484912 | 11 |
| Green Yellow | GO:0072006 | nephron development | 0.019746055 | 7 |
| Green Yellow | GO:0030323 | respiratory tube development | 0.020697159 | 8 |
| Green Yellow | GO:0014812 | muscle cell migration | 0.020968347 | 5 |
| Green Yellow | GO:1903035 | negative regulation of response to wounding | 0.020968347 | 5 |
| Green Yellow | GO:0090101 | negative regulation of transmembrane receptor protein serine/threonine kinase signaling pathway | 0.021867093 | 6 |
| Green Yellow | GO:0007045 | cell-substrate adherens junction assembly | 0.022075661 | 5 |
| Green Yellow | GO:0048041 | focal adhesion assembly | 0.022075661 | 5 |
| Green Yellow | GO:0050920 | regulation of chemotaxis | 0.02324015 | 8 |
| Green Yellow | GO:0035265 | organ growth | 0.024027771 | 7 |
| Green Yellow | GO:0010631 | epithelial cell migration | 0.024354978 | 9 |
| Green Yellow | GO:0072073 | kidney epithelium development | 0.024800704 | 7 |
| Green Yellow | GO:0048771 | tissue remodeling | 0.025479854 | 7 |
| Green Yellow | GO:0090132 | epithelium migration | 0.026102255 | 9 |
| Green Yellow | GO:0055123 | digestive system development | 0.026879107 | 7 |
| Green Yellow | GO:0006469 | negative regulation of protein kinase activity | 0.027010872 | 9 |
| Green Yellow | GO:0043271 | negative regulation of ion transport | 0.028068218 | 6 |
| Green Yellow | GO:0035107 | appendage morphogenesis | 0.028333788 | 7 |
| Green Yellow | GO:0035108 | limb morphogenesis | 0.028333788 | 7 |
| Green Yellow | GO:0001935 | endothelial cell proliferation | 0.028711375 | 6 |
| Green Yellow | GO:0060071 | Wnt signaling pathway; planar cell polarity pathway | 0.028711375 | 6 |
| Green Yellow | GO:0090130 | tissue migration | 0.029033369 | 9 |
| Green Yellow | GO:0090175 | regulation of establishment of planar polarity | 0.029314278 | 6 |
| Green Yellow | GO:0001666 | response to hypoxia | 0.029314278 | 10 |
| Green Yellow | GO:0050769 | positive regulation of neurogenesis | 0.030702923 | 12 |
| Green Yellow | GO:0001654 | eye development | 0.031414353 | 11 |
| Green Yellow | GO:0030510 | regulation of BMP signaling pathway | 0.0323122 | 5 |
| Green Yellow | GO:1903510 | mucopolysaccharide metabolic process | 0.032433276 | 6 |
| Green Yellow | GO:0001933 | negative regulation of protein phosphorylation | 0.032433276 | 12 |
| Green Yellow | GO:0009612 | response to mechanical stimulus | 0.032492572 | 8 |
| Green Yellow | GO:0051051 | negative regulation of transport | 0.032492572 | 13 |
| Green Yellow | GO:0034333 | adherens junction assembly | 0.033375974 | 5 |
| Green Yellow | GO:0036293 | response to decreased oxygen levels | 0.033756699 | 10 |
| Green Yellow | GO:0007389 | pattern specification process | 0.033756699 | 13 |
| Green Yellow | GO:0046620 | regulation of organ growth | 0.033960464 | 5 |
| Green Yellow | GO:0010720 | positive regulation of cell development | 0.034831484 | 13 |
| Green Yellow | GO:0060541 | respiratory system development | 0.034839691 | 8 |
| Green Yellow | GO:0048638 | regulation of developmental growth | 0.035087455 | 10 |
| Green Yellow | GO:0045165 | cell fate commitment | 0.035288426 | 9 |
| Green Yellow | GO:0033673 | negative regulation of kinase activity | 0.036722941 | 9 |
| Green Yellow | GO:0001736 | establishment of planar polarity | 0.037828091 | 6 |
| Green Yellow | GO:0007164 | establishment of tissue polarity | 0.037828091 | 6 |
| Green Yellow | GO:0071901 | negative regulation of protein serine/threonine kinase activity | 0.037828091 | 6 |
| Green Yellow | GO:0050878 | regulation of body fluid levels | 0.037828091 | 14 |
| Green Yellow | GO:0030193 | regulation of blood coagulation | 0.039637453 | 5 |
| Green Yellow | GO:1900046 | regulation of hemostasis | 0.039637453 | 5 |
| Green Yellow | GO:0007517 | muscle organ development | 0.039637453 | 11 |
| Green Yellow | GO:1902532 | negative regulation of intracellular signal transduction | 0.040308291 | 13 |
| Green Yellow | GO:0007044 | cell-substrate junction assembly | 0.040941995 | 5 |
| Green Yellow | GO:0060021 | palate development | 0.040941995 | 5 |
| Green Yellow | GO:0010977 | negative regulation of neuron projection development | 0.041435156 | 6 |
| Green Yellow | GO:0030308 | negative regulation of cell growth | 0.042486842 | 7 |
| Green Yellow | GO:0045666 | positive regulation of neuron differentiation | 0.043295516 | 10 |
| Green Yellow | GO:1901565 | organonitrogen compound catabolic process | 0.043587945 | 11 |
| Green Yellow | GO:0045861 | negative regulation of proteolysis | 0.043723647 | 10 |
| Green Yellow | GO:0046822 | regulation of nucleocytoplasmic transport | 0.043862883 | 8 |
| Green Yellow | GO:0007179 | transforming growth factor beta receptor signaling pathway | 0.044061348 | 7 |
| Green Yellow | GO:0048863 | stem cell differentiation | 0.044061348 | 7 |
| Green Yellow | GO:0010632 | regulation of epithelial cell migration | 0.045378661 | 7 |
| Green Yellow | GO:0050818 | regulation of coagulation | 0.045453178 | 5 |
| Green Yellow | GO:0048736 | appendage development | 0.047634066 | 7 |
| Green Yellow | GO:0060173 | limb development | 0.047634066 | 7 |
| Light Cyan | GO:0006397 | mRNA processing | 0.013185013 | 13 |
| Light Cyan | GO:0034248 | regulation of cellular amide metabolic process | 0.021020447 | 11 |
| Light Cyan | GO:0046034 | ATP metabolic process | 0.045852059 | 8 |
| PINK | GO:0010498 | proteasomal protein catabolic process | 0.004606497 | 22 |
| PINK | GO:0051568 | histone H3-K4 methylation | 0.02652121 | 7 |
| PINK | GO:0043161 | proteasome-mediated ubiquitin-dependent protein catabolic process | 0.02652121 | 19 |
| Royal Blue | GO:0007159 | leukocyte cell-cell adhesion | 1.86505E-24 | 32 |
| Royal Blue | GO:0070486 | leukocyte aggregation | 2.35407E-24 | 31 |
| Royal Blue | GO:0042110 | T cell activation | 1.27605E-23 | 30 |
| Royal Blue | GO:0070489 | T cell aggregation | 1.27605E-23 | 30 |
| Royal Blue | GO:0071593 | lymphocyte aggregation | 1.27605E-23 | 30 |
| Royal Blue | GO:0051249 | regulation of lymphocyte activation | 2.26743E-18 | 25 |
| Royal Blue | GO:0002250 | adaptive immune response | 6.5859E-18 | 24 |
| Royal Blue | GO:0030098 | lymphocyte differentiation | 3.14141E-17 | 22 |
| Royal Blue | GO:0002694 | regulation of leukocyte activation | 3.16715E-17 | 25 |
| Royal Blue | GO:0030217 | T cell differentiation | 9.97021E-17 | 19 |
| Royal Blue | GO:0050865 | regulation of cell activation | 1.42021E-16 | 25 |
| Royal Blue | GO:0002696 | positive regulation of leukocyte activation | 2.70644E-16 | 21 |
| Royal Blue | GO:0050867 | positive regulation of cell activation | 4.58874E-16 | 21 |
| Royal Blue | GO:0051251 | positive regulation of lymphocyte activation | 8.42422E-16 | 20 |
| Royal Blue | GO:0050863 | regulation of T cell activation | 3.51315E-14 | 19 |
| Royal Blue | GO:0002521 | leukocyte differentiation | 6.84915E-14 | 22 |
| Royal Blue | GO:1903037 | regulation of leukocyte cell-cell adhesion | 7.42152E-14 | 19 |
| Royal Blue | GO:0050851 | antigen receptor-mediated signaling pathway | 1.06398E-13 | 17 |
| Royal Blue | GO:0002768 | immune response-regulating cell surface receptor signaling pathway | 1.13943E-13 | 20 |
| Royal Blue | GO:0002429 | immune response-activating cell surface receptor signaling pathway | 2.85452E-13 | 19 |
| Royal Blue | GO:0050870 | positive regulation of T cell activation | 4.00848E-13 | 16 |
| Royal Blue | GO:1903039 | positive regulation of leukocyte cell-cell adhesion | 7.04248E-13 | 16 |
| Royal Blue | GO:0022407 | regulation of cell-cell adhesion | 2.665E-12 | 19 |
| Royal Blue | GO:0022409 | positive regulation of cell-cell adhesion | 4.5447E-12 | 16 |
| Royal Blue | GO:0050852 | T cell receptor signaling pathway | 8.20728E-12 | 14 |
| Royal Blue | GO:0002764 | immune response-regulating signaling pathway | 2.63207E-11 | 20 |
| Royal Blue | GO:0045058 | T cell selection | 3.45856E-11 | 9 |
| Royal Blue | GO:0002757 | immune response-activating signal transduction | 7.40912E-11 | 19 |
| Royal Blue | GO:0045785 | positive regulation of cell adhesion | 2.70955E-10 | 17 |
| Royal Blue | GO:0002443 | leukocyte mediated immunity | 1.96755E-09 | 15 |
| Royal Blue | GO:0050900 | leukocyte migration | 1.96755E-09 | 16 |
| Royal Blue | GO:0043368 | positive T cell selection | 3.67236E-09 | 7 |
| Royal Blue | GO:0050670 | regulation of lymphocyte proliferation | 7.8962E-09 | 12 |
| Royal Blue | GO:0032944 | regulation of mononuclear cell proliferation | 8.15875E-09 | 12 |
| Royal Blue | GO:0002449 | lymphocyte mediated immunity | 1.04189E-08 | 13 |
| Royal Blue | GO:0046631 | alpha-beta T cell activation | 1.04452E-08 | 10 |
| Royal Blue | GO:0070663 | regulation of leukocyte proliferation | 1.29547E-08 | 12 |
| Royal Blue | GO:0046651 | lymphocyte proliferation | 1.44304E-08 | 13 |
| Royal Blue | GO:0032943 | mononuclear cell proliferation | 1.55344E-08 | 13 |
| Royal Blue | GO:0046632 | alpha-beta T cell differentiation | 2.22596E-08 | 9 |
| Royal Blue | GO:0050864 | regulation of B cell activation | 2.34266E-08 | 10 |
| Royal Blue | GO:0050671 | positive regulation of lymphocyte proliferation | 2.68516E-08 | 10 |
| Royal Blue | GO:0032946 | positive regulation of mononuclear cell proliferation | 2.82967E-08 | 10 |
| Royal Blue | GO:0070661 | leukocyte proliferation | 2.82967E-08 | 13 |
| Royal Blue | GO:0070665 | positive regulation of leukocyte proliferation | 4.30237E-08 | 10 |
| Royal Blue | GO:0045619 | regulation of lymphocyte differentiation | 6.07511E-08 | 10 |
| Royal Blue | GO:0042113 | B cell activation | 9.47817E-08 | 12 |
| Royal Blue | GO:0002366 | leukocyte activation involved in immune response | 3.78259E-07 | 11 |
| Royal Blue | GO:0002263 | cell activation involved in immune response | 4.08069E-07 | 11 |
| Royal Blue | GO:0042102 | positive regulation of T cell proliferation | 7.33659E-07 | 8 |
| Royal Blue | GO:0002460 | adaptive immune response based on somatic recombination of immune receptors built from immunoglobulin superfamily domains | 1.06549E-06 | 11 |
| Royal Blue | GO:0042129 | regulation of T cell proliferation | 1.17186E-06 | 9 |
| Royal Blue | GO:0033077 | T cell differentiation in thymus | 1.50901E-06 | 7 |
| Royal Blue | GO:0071594 | thymocyte aggregation | 1.50901E-06 | 7 |
| Royal Blue | GO:0002703 | regulation of leukocyte mediated immunity | 1.89999E-06 | 9 |
| Royal Blue | GO:0002285 | lymphocyte activation involved in immune response | 2.09345E-06 | 9 |
| Royal Blue | GO:0045580 | regulation of T cell differentiation | 2.41188E-06 | 8 |
| Royal Blue | GO:0031295 | T cell costimulation | 4.01338E-06 | 7 |
| Royal Blue | GO:0031294 | lymphocyte costimulation | 4.234E-06 | 7 |
| Royal Blue | GO:0042098 | T cell proliferation | 4.234E-06 | 9 |
| Royal Blue | GO:0002705 | positive regulation of leukocyte mediated immunity | 6.87082E-06 | 7 |
| Royal Blue | GO:1902105 | regulation of leukocyte differentiation | 6.98331E-06 | 10 |
| Royal Blue | GO:0050871 | positive regulation of B cell activation | 7.20483E-06 | 7 |
| Royal Blue | GO:0002228 | natural killer cell mediated immunity | 1.03686E-05 | 6 |
| Royal Blue | GO:1903706 | regulation of hemopoiesis | 1.31828E-05 | 11 |
| Royal Blue | GO:0032609 | interferon-gamma production | 1.78504E-05 | 7 |
| Royal Blue | GO:0002708 | positive regulation of lymphocyte mediated immunity | 3.5832E-05 | 6 |
| Royal Blue | GO:0002706 | regulation of lymphocyte mediated immunity | 3.62396E-05 | 7 |
| Royal Blue | GO:0030101 | natural killer cell activation | 4.449E-05 | 6 |
| Royal Blue | GO:0002683 | negative regulation of immune system process | 5.05645E-05 | 11 |
| Royal Blue | GO:2000107 | negative regulation of leukocyte apoptotic process | 5.81701E-05 | 5 |
| Royal Blue | GO:0045621 | positive regulation of lymphocyte differentiation | 6.10947E-05 | 6 |
| Royal Blue | GO:2000106 | regulation of leukocyte apoptotic process | 6.10947E-05 | 6 |
| Royal Blue | GO:0001909 | leukocyte mediated cytotoxicity | 6.49231E-05 | 6 |
| Royal Blue | GO:0072676 | lymphocyte migration | 6.8934E-05 | 6 |
| Royal Blue | GO:0002697 | regulation of immune effector process | 7.85647E-05 | 10 |
| Royal Blue | GO:0032655 | regulation of interleukin-12 production | 9.2586E-05 | 5 |
| Royal Blue | GO:0032615 | interleukin-12 production | 0.000110377 | 5 |
| Royal Blue | GO:0032649 | regulation of interferon-gamma production | 0.000126174 | 6 |
| Royal Blue | GO:0030888 | regulation of B cell proliferation | 0.000127532 | 5 |
| Royal Blue | GO:0042267 | natural killer cell mediated cytotoxicity | 0.000127532 | 5 |
| Royal Blue | GO:0070228 | regulation of lymphocyte apoptotic process | 0.000138369 | 5 |
| Royal Blue | GO:0019722 | calcium-mediated signaling | 0.000167544 | 7 |
| Royal Blue | GO:0071887 | leukocyte apoptotic process | 0.000172243 | 6 |
| Royal Blue | GO:0002699 | positive regulation of immune effector process | 0.000224512 | 7 |
| Royal Blue | GO:0001906 | cell killing | 0.000254302 | 6 |
| Royal Blue | GO:0001819 | positive regulation of cytokine production | 0.000336536 | 10 |
| Royal Blue | GO:0043122 | regulation of I-kappaB kinase/NF-kappaB signaling | 0.000353422 | 8 |
| Royal Blue | GO:0030183 | B cell differentiation | 0.000356327 | 6 |
| Royal Blue | GO:0070227 | lymphocyte apoptotic process | 0.000360539 | 5 |
| Royal Blue | GO:0009615 | response to virus | 0.00036235 | 9 |
| Royal Blue | GO:0051250 | negative regulation of lymphocyte activation | 0.00036398 | 6 |
| Royal Blue | GO:0046634 | regulation of alpha-beta T cell activation | 0.00037328 | 5 |
| Royal Blue | GO:0043123 | positive regulation of I-kappaB kinase/NF-kappaB signaling | 0.000462063 | 7 |
| Royal Blue | GO:0031349 | positive regulation of defense response | 0.000462063 | 10 |
| Royal Blue | GO:0002819 | regulation of adaptive immune response | 0.000534509 | 6 |
| Royal Blue | GO:0030595 | leukocyte chemotaxis | 0.000541361 | 7 |
| Royal Blue | GO:0050848 | regulation of calcium-mediated signaling | 0.000545267 | 5 |
| Royal Blue | GO:1902107 | positive regulation of leukocyte differentiation | 0.000555956 | 6 |
| Royal Blue | GO:0007249 | I-kappaB kinase/NF-kappaB signaling | 0.000642438 | 8 |
| Royal Blue | GO:0070098 | chemokine-mediated signaling pathway | 0.000650519 | 5 |
| Royal Blue | GO:0002695 | negative regulation of leukocyte activation | 0.000747338 | 6 |
| Royal Blue | GO:0042100 | B cell proliferation | 0.000896073 | 5 |
| Royal Blue | GO:0002286 | T cell activation involved in immune response | 0.001031332 | 5 |
| Royal Blue | GO:0002685 | regulation of leukocyte migration | 0.001158237 | 6 |
| Royal Blue | GO:0001818 | negative regulation of cytokine production | 0.001158237 | 7 |
| Royal Blue | GO:0050866 | negative regulation of cell activation | 0.001285111 | 6 |
| Royal Blue | GO:0048872 | homeostasis of number of cells | 0.001310915 | 7 |
| Royal Blue | GO:1903708 | positive regulation of hemopoiesis | 0.001526713 | 6 |
| Royal Blue | GO:0032680 | regulation of tumor necrosis factor production | 0.001718359 | 5 |
| Royal Blue | GO:0032640 | tumor necrosis factor production | 0.001822077 | 5 |
| Royal Blue | GO:0019932 | second-messenger-mediated signaling | 0.001861062 | 7 |
| Royal Blue | GO:1903555 | regulation of tumor necrosis factor superfamily cytokine production | 0.001881518 | 5 |
| Royal Blue | GO:0060326 | cell chemotaxis | 0.002131926 | 7 |
| Royal Blue | GO:0071706 | tumor necrosis factor superfamily cytokine production | 0.002185078 | 5 |
| Royal Blue | GO:0032845 | negative regulation of homeostatic process | 0.002453093 | 6 |
| Royal Blue | GO:0002822 | regulation of adaptive immune response based on somatic recombination of immune receptors built from immunoglobulin superfamily domains | 0.002476957 | 5 |
| Royal Blue | GO:0050777 | negative regulation of immune response | 0.002670898 | 5 |
| Royal Blue | GO:0045088 | regulation of innate immune response | 0.00359451 | 8 |
| Royal Blue | GO:0018108 | peptidyl-tyrosine phosphorylation | 0.003977141 | 8 |
| Royal Blue | GO:0018212 | peptidyl-tyrosine modification | 0.004103899 | 8 |
| Royal Blue | GO:0019724 | B cell mediated immunity | 0.004537731 | 5 |
| Royal Blue | GO:0045089 | positive regulation of innate immune response | 0.005473838 | 7 |
| Royal Blue | GO:0014066 | regulation of phosphatidylinositol 3-kinase signaling | 0.006163385 | 5 |
| Royal Blue | GO:0032496 | response to lipopolysaccharide | 0.006615772 | 7 |
| Royal Blue | GO:0051607 | defense response to virus | 0.008117154 | 6 |
| Royal Blue | GO:0002237 | response to molecule of bacterial origin | 0.008176827 | 7 |
| Royal Blue | GO:0002440 | production of molecular mediator of immune response | 0.009060965 | 5 |
| Royal Blue | GO:0014065 | phosphatidylinositol 3-kinase signaling | 0.009648743 | 5 |
| Royal Blue | GO:0007259 | JAK-STAT cascade | 0.010767913 | 5 |
| Royal Blue | GO:0097696 | STAT cascade | 0.010767913 | 5 |
| Royal Blue | GO:0050920 | regulation of chemotaxis | 0.014455179 | 5 |
| Royal Blue | GO:0006874 | cellular calcium ion homeostasis | 0.018393535 | 7 |
| Royal Blue | GO:0055074 | calcium ion homeostasis | 0.021330196 | 7 |
| Royal Blue | GO:0048015 | phosphatidylinositol-mediated signaling | 0.021525263 | 5 |
| Royal Blue | GO:0048017 | inositol lipid-mediated signaling | 0.022568339 | 5 |
| Royal Blue | GO:0072503 | cellular divalent inorganic cation homeostasis | 0.024145744 | 7 |
| Royal Blue | GO:0072507 | divalent inorganic cation homeostasis | 0.030313213 | 7 |
| Royal Blue | GO:0046777 | protein autophosphorylation | 0.033073138 | 5 |
| Royal Blue | GO:0006909 | phagocytosis | 0.039332508 | 5 |
| Royal Blue | GO:0007204 | positive regulation of cytosolic calcium ion concentration | 0.046191334 | 5 |
| Royal Blue | GO:0009306 | protein secretion | 0.046191334 | 7 |
| salmon | GO:0017148 | negative regulation of translation | 0.003506682 | 10 |
| salmon | GO:0034249 | negative regulation of cellular amide metabolic process | 0.003506682 | 10 |
| salmon | GO:0006417 | regulation of translation | 0.006093621 | 14 |
| salmon | GO:0034248 | regulation of cellular amide metabolic process | 0.012296838 | 14 |
| salmon | GO:1902115 | regulation of organelle assembly | 0.023462444 | 8 |
| salmon | GO:0010608 | posttranscriptional regulation of gene expression | 0.023462444 | 15 |
| salmon | GO:0007059 | chromosome segregation | 0.027103856 | 12 |
| Tan | GO:0002495 | antigen processing and presentation of peptide antigen via MHC class II | 1.55563E-06 | 12 |
| Tan | GO:0002504 | antigen processing and presentation of peptide or polysaccharide antigen via MHC class II | 1.55563E-06 | 12 |
| Tan | GO:0019886 | antigen processing and presentation of exogenous peptide antigen via MHC class II | 7.50446E-06 | 11 |
| Tan | GO:0048002 | antigen processing and presentation of peptide antigen | 1.33918E-05 | 14 |
| Tan | GO:0006826 | iron ion transport | 0.000138355 | 8 |
| Tan | GO:0019882 | antigen processing and presentation | 0.000138355 | 14 |
| Tan | GO:1903037 | regulation of leukocyte cell-cell adhesion | 0.000175079 | 16 |
| Tan | GO:0044283 | small molecule biosynthetic process | 0.000241568 | 20 |
| Tan | GO:0009395 | phospholipid catabolic process | 0.000304492 | 6 |
| Tan | GO:0046503 | glycerolipid catabolic process | 0.000304492 | 7 |
| Tan | GO:0050863 | regulation of T cell activation | 0.000304492 | 15 |
| Tan | GO:0002694 | regulation of leukocyte activation | 0.000304492 | 19 |
| Tan | GO:0046486 | glycerolipid metabolic process | 0.000365014 | 17 |
| Tan | GO:0006644 | phospholipid metabolic process | 0.000466471 | 17 |
| Tan | GO:0002478 | antigen processing and presentation of exogenous peptide antigen | 0.000495721 | 11 |
| Tan | GO:0050865 | regulation of cell activation | 0.000524425 | 19 |
| Tan | GO:0051249 | regulation of lymphocyte activation | 0.000541634 | 17 |
| Tan | GO:0000041 | transition metal ion transport | 0.000547679 | 9 |
| Tan | GO:0019884 | antigen processing and presentation of exogenous antigen | 0.000589833 | 11 |
| Tan | GO:0016042 | lipid catabolic process | 0.000880684 | 14 |
| Tan | GO:0022407 | regulation of cell-cell adhesion | 0.00088603 | 16 |
| Tan | GO:0006639 | acylglycerol metabolic process | 0.001036554 | 9 |
| Tan | GO:0006638 | neutral lipid metabolic process | 0.001064083 | 9 |
| Tan | GO:0006641 | triglyceride metabolic process | 0.002657818 | 8 |
| Tan | GO:0042129 | regulation of T cell proliferation | 0.003481034 | 9 |
| Tan | GO:1903039 | positive regulation of leukocyte cell-cell adhesion | 0.003481034 | 11 |
| Tan | GO:0046394 | carboxylic acid biosynthetic process | 0.003607192 | 12 |
| Tan | GO:0033572 | transferrin transport | 0.004322652 | 5 |
| Tan | GO:0044242 | cellular lipid catabolic process | 0.004645673 | 10 |
| Tan | GO:0015682 | ferric iron transport | 0.004859139 | 5 |
| Tan | GO:0072512 | trivalent inorganic cation transport | 0.004859139 | 5 |
| Tan | GO:0050670 | regulation of lymphocyte proliferation | 0.004859139 | 10 |
| Tan | GO:0032944 | regulation of mononuclear cell proliferation | 0.004859139 | 10 |
| Tan | GO:0016053 | organic acid biosynthetic process | 0.004894492 | 12 |
| Tan | GO:0006790 | sulfur compound metabolic process | 0.004894492 | 14 |
| Tan | GO:0006027 | glycosaminoglycan catabolic process | 0.00508625 | 6 |
| Tan | GO:0070663 | regulation of leukocyte proliferation | 0.006243188 | 10 |
| Tan | GO:0045806 | negative regulation of endocytosis | 0.006874617 | 5 |
| Tan | GO:0022409 | positive regulation of cell-cell adhesion | 0.006874617 | 11 |
| Tan | GO:0006026 | aminoglycan catabolic process | 0.006991093 | 6 |
| Tan | GO:0030574 | collagen catabolic process | 0.007297558 | 6 |
| Tan | GO:0042098 | T cell proliferation | 0.007297558 | 9 |
| Tan | GO:0050870 | positive regulation of T cell activation | 0.007297558 | 10 |
| Tan | GO:0006898 | receptor-mediated endocytosis | 0.007297558 | 12 |
| Tan | GO:0007159 | leukocyte cell-cell adhesion | 0.00732412 | 16 |
| Tan | GO:0042632 | cholesterol homeostasis | 0.007683685 | 6 |
| Tan | GO:0055092 | sterol homeostasis | 0.007683685 | 6 |
| Tan | GO:0042110 | T cell activation | 0.008548132 | 15 |
| Tan | GO:0070489 | T cell aggregation | 0.008548132 | 15 |
| Tan | GO:0071593 | lymphocyte aggregation | 0.00859511 | 15 |
| Tan | GO:0002764 | immune response-regulating signaling pathway | 0.009039786 | 16 |
| Tan | GO:0044243 | multicellular organism catabolic process | 0.009565532 | 6 |
| Tan | GO:1901136 | carbohydrate derivative catabolic process | 0.009565532 | 9 |
| Tan | GO:0010876 | lipid localization | 0.009565532 | 13 |
| Tan | GO:0031349 | positive regulation of defense response | 0.009565532 | 14 |
| Tan | GO:0070486 | leukocyte aggregation | 0.009565532 | 15 |
| Tan | GO:0072330 | monocarboxylic acid biosynthetic process | 0.010946971 | 9 |
| Tan | GO:0032963 | collagen metabolic process | 0.011165582 | 7 |
| Tan | GO:0048511 | rhythmic process | 0.011282495 | 12 |
| Tan | GO:0002757 | immune response-activating signal transduction | 0.011666846 | 15 |
| Tan | GO:0006869 | lipid transport | 0.011926519 | 12 |
| Tan | GO:0031295 | T cell costimulation | 0.0124037 | 6 |
| Tan | GO:0031294 | lymphocyte costimulation | 0.012825248 | 6 |
| Tan | GO:0044259 | multicellular organismal macromolecule metabolic process | 0.013003538 | 7 |
| Tan | GO:0046887 | positive regulation of hormone secretion | 0.013329328 | 7 |
| Tan | GO:0051186 | cofactor metabolic process | 0.013329328 | 13 |
| Tan | GO:0006650 | glycerophospholipid metabolic process | 0.013673427 | 11 |
| Tan | GO:0002699 | positive regulation of immune effector process | 0.015175321 | 8 |
| Tan | GO:0097006 | regulation of plasma lipoprotein particle levels | 0.016175479 | 5 |
| Tan | GO:0046434 | organophosphate catabolic process | 0.016175479 | 7 |
| Tan | GO:0015850 | organic hydroxy compound transport | 0.016446855 | 9 |
| Tan | GO:0046651 | lymphocyte proliferation | 0.018457181 | 10 |
| Tan | GO:0032943 | mononuclear cell proliferation | 0.018457181 | 10 |
| Tan | GO:0046890 | regulation of lipid biosynthetic process | 0.018951948 | 7 |
| Tan | GO:0044236 | multicellular organism metabolic process | 0.021323324 | 7 |
| Tan | GO:0002696 | positive regulation of leukocyte activation | 0.021323324 | 11 |
| Tan | GO:0002695 | negative regulation of leukocyte activation | 0.025283048 | 7 |
| Tan | GO:0050867 | positive regulation of cell activation | 0.025283048 | 11 |
| Tan | GO:0070661 | leukocyte proliferation | 0.025663608 | 10 |
| Tan | GO:0002697 | regulation of immune effector process | 0.025663608 | 11 |
| Tan | GO:0002700 | regulation of production of molecular mediator of immune response | 0.029174257 | 6 |
| Tan | GO:0006633 | fatty acid biosynthetic process | 0.029174257 | 7 |
| Tan | GO:0042445 | hormone metabolic process | 0.029174257 | 8 |
| Tan | GO:0051251 | positive regulation of lymphocyte activation | 0.033836874 | 10 |
| Tan | GO:0030301 | cholesterol transport | 0.037049084 | 5 |
| Tan | GO:0015918 | sterol transport | 0.037768222 | 5 |
| Tan | GO:0007623 | circadian rhythm | 0.037768222 | 8 |
| Tan | GO:0006066 | alcohol metabolic process | 0.037768222 | 10 |
| Tan | GO:0044723 | single-organism carbohydrate metabolic process | 0.040089504 | 13 |
| Tan | GO:0070301 | cellular response to hydrogen peroxide | 0.04118313 | 5 |
| Tan | GO:0050866 | negative regulation of cell activation | 0.04118313 | 7 |
| Tan | GO:0051250 | negative regulation of lymphocyte activation | 0.045571367 | 6 |
| Tan | GO:0050729 | positive regulation of inflammatory response | 0.047234882 | 6 |
| Tan | GO:1903708 | positive regulation of hemopoiesis | 0.049802156 | 7 |
| Tan | GO:0044272 | sulfur compound biosynthetic process | 0.049802156 | 8 |
| Tan | GO:0044706 | multi-multicellular organism process | 0.049802156 | 8 |

**Table S6:** Genes in modules of interest differentially expressed in the LTRC dataset GEO# GSE47460; platform#GPL14550, in the comparison of Controls vs. COPD, logFC>|0.3| and FDR p value < 0.05.

|  | **Module** | **symbol** | **adj.P.Val** | **logFC** |  | **Module** | **symbol** | **adj.P.Val** | **logFC** |
| --- | --- | --- | --- | --- | --- | --- | --- | --- | --- |
| 1 | yellow | FGG | 5.54E-06 | -1.92 | 1 | yellow | KRT4 | 2.10E-06 | 0.93 |
| 2 | greenyellow | PLA2G2A | 2.21E-03 | -1.03 | 2 | yellow | SLITRK6 | 1.76E-04 | 0.76 |
| 3 | blue | BNC1 | 9.91E-04 | -0.99 | 3 | yellow | CDHR3 | 2.57E-02 | 0.70 |
| 4 | greenyellow | POU2AF1 | 6.61E-05 | -0.93 | 4 | yellow | CFAP74 | 3.68E-02 | 0.60 |
| 5 | greenyellow | MZB1 | 1.96E-04 | -0.84 | 5 | yellow | TNNI3 | 1.86E-03 | 0.56 |
| 6 | greenyellow | TNFRSF17 | 7.95E-04 | -0.76 | 6 | yellow | SEC14L4 | 7.42E-05 | 0.55 |
| 7 | greenyellow | IGDCC4 | 4.07E-05 | -0.68 | 7 | blue | PRPH | 9.61E-06 | 0.54 |
| 8 | greenyellow | CPXM1 | 3.28E-03 | -0.68 | 8 | yellow | SEC14L3 | 4.30E-02 | 0.53 |
| 9 | greenyellow | COL14A1 | 5.88E-05 | -0.64 | 9 | yellow | PRMT8 | 1.07E-02 | 0.51 |
| 10 | greenyellow | CTHRC1 | 1.50E-03 | -0.61 | 10 | yellow | VWA3A | 3.99E-02 | 0.46 |
| 11 | greenyellow | IGDCC4 | 4.07E-05 | -0.68 | 11 | blue | PRPH | 9.61E-06 | 0.54 |
| 12 | greenyellow | CPXM1 | 3.28E-03 | -0.68 | 12 | yellow | SEC14L3 | 4.30E-02 | 0.53 |
| 13 | greenyellow | COL14A1 | 5.88E-05 | -0.64 | 13 | yellow | PRMT8 | 1.07E-02 | 0.51 |
| 14 | greenyellow | CTHRC1 | 1.50E-03 | -0.61 | 14 | yellow | VWA3A | 3.99E-02 | 0.46 |
| 15 | blue | MMP11 | 3.22E-03 | -0.60 | 15 | yellow | MISP | 6.32E-04 | 0.43 |
| 16 | greenyellow | AMPD1 | 9.62E-03 | -0.59 | 16 | yellow | TPPP3 | 4.98E-03 | 0.42 |
| 17 | greenyellow | SFRP2 | 3.38E-02 | -0.57 | 17 | yellow | KIAA0319 | 3.69E-02 | 0.42 |
| 18 | royalblue | CDCA7 | 4.71E-04 | -0.55 | 18 | yellow | RP1 | 4.89E-02 | 0.37 |
| 19 | greenyellow | FRZB | 1.09E-06 | -0.55 | 19 | yellow | ZNF365 | 1.60E-04 | 0.36 |
| 20 | greenyellow | MEOX1 | 3.04E-02 | -0.55 | 20 | yellow | CXorf57 | 2.42E-04 | 0.36 |
| 21 | greenyellow | CCDC80 | 2.77E-03 | -0.54 | 21 | yellow | PPIL6 | 4.65E-02 | 0.36 |
| 22 | greenyellow | IGF1 | 9.32E-04 | -0.53 | 22 | yellow | SLC16A5 | 1.48E-07 | 0.35 |
| 23 | greenyellow | SERPINF1 | 1.89E-05 | -0.52 | 23 | yellow | MYOT | 2.61E-02 | 0.34 |
| 24 | greenyellow | SMOC2 | 1.54E-04 | -0.52 | 24 | tan | PDE1C | 9.40E-04 | 0.34 |
| 25 | greenyellow | PDGFD | 1.28E-03 | -0.51 | 25 | yellow | USP43 | 9.17E-03 | 0.33 |
| 26 | yellow | SLC2A5 | 9.44E-03 | -0.50 |  |  |  |  |  |
| 27 | greenyellow | SERPINE2 | 3.67E-05 | -0.49 |  |  |  |  |  |
| 28 | greenyellow | SULF1 | 3.92E-04 | -0.48 |  |  |  |  |  |
| 29 | tan | TREM2 | 6.55E-03 | -0.47 |  |  |  |  |  |
| 30 | royalblue | CD27 | 3.70E-03 | -0.47 |  |  |  |  |  |
| 31 | greenyellow | SPAG4 | 2.44E-03 | -0.46 |  |  |  |  |  |
| 32 | greenyellow | PODN | 1.17E-03 | -0.46 |  |  |  |  |  |
| 33 | greenyellow | ANGPT2 | 3.66E-03 | -0.45 |  |  |  |  |  |
| 34 | yellow | GAPT | 1.93E-04 | -0.44 |  |  |  |  |  |
| 35 | royalblue | GPR18 | 1.56E-03 | -0.44 |  |  |  |  |  |
| 36 | lightcyan | PFKL | 4.29E-03 | -0.43 |  |  |  |  |  |
| 37 | royalblue | SLAMF7 | 4.81E-03 | -0.43 |  |  |  |  |  |
| 38 | greenyellow | EGFLAM | 2.56E-03 | -0.43 |  |  |  |  |  |
| 39 | yellow | STK32B | 1.52E-03 | -0.43 |  |  |  |  |  |
| 40 | greenyellow | LUM | 2.01E-06 | -0.42 |  |  |  |  |  |
| 41 | greenyellow | CXCL12 | 3.98E-04 | -0.42 |  |  |  |  |  |
| 42 | greenyellow | DOK5 | 4.87E-03 | -0.41 |  |  |  |  |  |
| 43 | greenyellow | PDE1A | 4.77E-04 | -0.41 |  |  |  |  |  |
| 44 | greenyellow | FBLN2 | 1.18E-03 | -0.41 |  |  |  |  |  |
| 45 | greenyellow | CTSK | 1.76E-03 | -0.41 |  |  |  |  |  |
| 46 | greenyellow | SCN1B | 1.34E-04 | -0.40 |  |  |  |  |  |
| 47 | greenyellow | C7 | 2.36E-03 | -0.40 |  |  |  |  |  |
| 48 | yellow | C21orf58 | 1.89E-03 | -0.40 |  |  |  |  |  |
| 49 | greenyellow | ZNF385D | 4.07E-02 | -0.40 |  |  |  |  |  |
| 50 | yellow | PZP | 7.36E-03 | -0.39 |  |  |  |  |  |
| 51 | royalblue | ACAP1 | 6.43E-04 | -0.39 |  |  |  |  |  |
| 52 | greenyellow | MOXD1 | 5.71E-04 | -0.38 |  |  |  |  |  |
| 53 | greenyellow | MFAP2 | 3.38E-04 | -0.38 |  |  |  |  |  |
| 54 | greenyellow | FAP | 3.03E-03 | -0.38 |  |  |  |  |  |
| 55 | greenyellow | CYTL1 | 4.53E-02 | -0.38 |  |  |  |  |  |
| 56 | greenyellow | PLPPR4 | 3.50E-03 | -0.38 |  |  |  |  |  |
| 57 | greenyellow | OAF | 1.14E-03 | -0.38 |  |  |  |  |  |
| 58 | greenyellow | NLGN1 | 2.18E-02 | -0.38 |  |  |  |  |  |
| 59 | royalblue | JAK3 | 7.35E-04 | -0.36 |  |  |  |  |  |
| 60 | royalblue | CCR7 | 2.14E-02 | -0.36 |  |  |  |  |  |
| 61 | greenyellow | FMO1 | 1.39E-02 | -0.36 |  |  |  |  |  |
| 62 | greenyellow | CPXM2 | 2.01E-02 | -0.36 |  |  |  |  |  |
| 63 | greenyellow | SEMA3D | 6.64E-04 | -0.35 |  |  |  |  |  |
| 64 | greenyellow | SPON1 | 1.97E-06 | -0.35 |  |  |  |  |  |
| 65 | royalblue | CD48 | 7.83E-04 | -0.34 |  |  |  |  |  |
| 66 | blue | RAB3IL1 | 1.62E-04 | -0.34 |  |  |  |  |  |
| 67 | greenyellow | LOX | 1.97E-03 | -0.34 |  |  |  |  |  |
| 68 | greenyellow | PLTP | 1.56E-03 | -0.33 |  |  |  |  |  |
| 69 | tan | INHBA | 3.81E-02 | -0.33 |  |  |  |  |  |
| 70 | greenyellow | PAMR1 | 3.37E-02 | -0.32 |  |  |  |  |  |
| 71 | greenyellow | PTGIS | 3.37E-03 | -0.32 |  |  |  |  |  |
| 72 | greenyellow | SULF2 | 8.94E-04 | -0.31 |  |  |  |  |  |
| 73 | greenyellow | SRPX2 | 7.33E-04 | -0.31 |  |  |  |  |  |
| 74 | royalblue | ICAM3 | 1.29E-04 | -0.31 |  |  |  |  |  |
| 75 | tan | LIPA | 1.58E-02 | -0.30 |  |  |  |  |  |
| 76 | tan | VMO1 | 7.94E-03 | -0.30 |  |  |  |  |  |
| 77 | greenyellow | PLVAP | 4.77E-02 | -0.30 |  |  |  |  |  |
| 78 | tan | FOLR2 | 8.81E-03 | -0.30 |  |  |  |  |  |

Supplementary Figure legends

Figure S1: Flow cytometry gating strategy for the neutrophil population: A) in lung tissue and B) peripheral blood. G1 determined the cell population by FSC vs. SSC and then the exclusion of cell aggregates is performed using the FSC-A *vs.* FSC-H (not shown), G2 the hematopoietic CD45+ cells and in lung tissue (b) G3 select the no auto fluorescence cells using two empty channels with fluorochrome. Neutrophils are selected from G3 as the CD15+ and CD16+ cells.

Figure S2: Flow cytometry gating strategy for the T lymphocyte populations: A) in lung tissue and B) peripheral blood. G1 determine the cell population by FSC vs. SSC, then the exclusion of cell aggregates is performed using the FSC-A *vs.* FSC-H (not shown), G2 the hematopoietic CD45+ cells and G3 the small cells corresponding to the whole lymphocytes and avoiding bigger and more fluorescent cells. T lymphocytes were selected by the expression of CD3 (G4) and then CD4 (G5) and CD8 (G6) population were identified. Finally, in these two populations the expression of the CD28 is evaluated (G7 and G8). To fix the gate for CD28 the FMO tube of lymphocytes (tube 10) was used.

Figure S3: Flow cytometry gating strategy for the B and NK lymphocyte populations: A) in lung tissue and B) peripheral blood. G1 determine the cell population by FSC vs. SSC, then the exclusion of cell aggregates is performed using the FSC-A *vs.* FSC-H (not shown), G2 the hematopoietic CD45+ cells and G3 the small cells corresponding to the whole lymphocytes and avoiding bigger and more fluorescent cells. From the lymphocyte population (G3) the CD3^-^CD56^+^CD16^+^ population represents the NK cells (G4) and the CD45^+^CD19^+^ population the B cells (G5).

Figure S4: Flow cytometry gating strategy for the NKT lymphocyte population: A) in lung tissue and B) peripheral blood. G1 determine the cell population by FSC vs. SSC, then the exclusion of cell aggregates is performed using the FSC-A *vs.* FSC-H (not shown), G2 selects the hematopoietic CD45+ cells and G3 the lymphocytes. **From the lymphocyte population (G4) NKT-like lymphocytes are selected as CD3+CD56+ , the gate in each experiment was set with a FMO tube (Tube 10, FMO for lymphocytes without the CD56 ). Then in NKT cells the expression of the CD4 and CD8 was evaluated.**

Figure S5: Flow cytometry gating strategy for the Dendritic cells (DC) populations: A) in lung tissue and B) peripheral blood. For lung tissue (A) G1 determine the cell population by FSC vs. SSC, then the exclusion of cell aggregates is performed using the FSC-A *vs.* FSC-H (G2). Next the hematopoietic CD45+ cells were selected (G3) and the autofluorescence was excluded with two channels without any fluorochrome (G4). From G4 the DCs are first split in two populations according to the expression of the CD11c (G5 and G6) and then in each one the expression of CD11b and HLA-DR is evaluated giving the final 4 DCs population: CD11c^high^HLA-DR^high^CD11b+ (G7), CD11c^high^HLA-DR^+^CD11b^high^ (G8), CD11c^low^HLA-DR^+^CD11b^low^ (G10) and CD11c^low^HLA-DR^+^CD11b^high^ (G9). In the final analysis total DCs are analysed as the sum of these 4 populations. In peripheral blood (b) G1 select the live cells, G2 the hematopoietic CD45+ cells and mononuclear cells are selected using the FSC *vs.* SSC (G3). Then the expression of CD11c and HLA-DR is evaluated to define the DCs (G4), this gate is not able to exclude NK cells expressing these markers.

Figure S6: Flow cytometry gating strategy for the monocyte and macrophage populations: A) in lung tissue and B) peripheral blood. For lung tissue (A) G1 determines the cell population, the autofluorescence was excluded with two channels without any fluorochrome, and then the hematopoietic CD45+ cells are selected (G2). SSC vs. CD64 plot is used to select the macrophages (G3) and monocytes (G4). In these two populations the expression of the M1 and M2 markers CD80 and CD163 is evaluated. To obtain the fraction of M1 (G5), M2 (G6) and M1-2 (G7) macrophages two FMO tubes (Tubes 11 and 12) are used to set the gates. In blood (b) the CD64 and CD33 markers are evaluated for the monocyte population (G3).

Figure S7: Box plot representing T lymphocyte subtypes in lung tissue of the four study groups*: %CD3+* CD4+ (*Panel A*) and % CD3+CD8+ T lymphocytes (*Panel B*),. For further explanations, see text.

Figure S8: Box plot representing the proportion of CD80+CD163- macrophages (*Panel A*), CD80+CD163+ macrophages (*Panel B*), CD80-CD163+ macrophages (Panel C), and Monocytes (Panel D) in lung tissue of the four study groups. For further explanations, see text.

Figure S9: Heat-map of module association with: the percentage of Macrophages, and those expressing different cell surface markers, adjusted by the level of FEV_1_ % ref.. The top number in each cell corresponds to the effect from the linear regression and the bottom number is the p-value. In the box, the summarizing terms for the Gene Ontology (GO) terms significantly enriched for this module obtained from the Revigo analysis are displayed. The full list of GO terms if provided in Table S5. For further explanations, see text.

1. Vogelmeier CF, Criner GJ, Martinez FJ, Anzueto A, Barnes PJ, Bourbeau J, Celli BR, Chen R, Decramer M, Fabbri LM, et al: **Global Strategy for the Diagnosis, Management, and Prevention of Chronic Obstructive Lung Disease 2017 Report. GOLD Executive Summary.** *Am J Respir Crit Care Med* 2017, **195:**557-582.

2. Irizarry RA, Hobbs B, Collin F, Beazer-Barclay YD, Antonellis KJ, Scherf U, Speed TP: **Exploration, normalization, and summaries of high density oligonucleotide array probe level data.** *Biostatistics* 2003, **4:**249-264.

3. Miller JA, Cai C, Langfelder P, Geschwind DH, Kurian SM, Salomon DR, Horvath S: **Strategies for aggregating gene expression data: the collapseRows R function.** *BMC bioinformatics* 2011, **12:**322.

4. Ritchie ME, Phipson B, Wu D, Hu Y, Law CW, Shi W, Smyth GK: **limma powers differential expression analyses for RNA-sequencing and microarray studies.** *Nucleic Acids Res* 2015, **43:**e47.

5. Yu G, Wang LG, Han Y, He QY: **clusterProfiler: an R package for comparing biological themes among gene clusters.** *OMICS* 2012, **16:**284-287.

6. Supek F, Bosnjak M, Skunca N, Smuc T: **REVIGO summarizes and visualizes long lists of gene ontology terms.** *PLoS One* 2011, **6:**e21800.
