# Supplementary material for: Multi-level immune response network in mild-moderate Chronic Obstructive Pulmonary Disease (COPD)
Source: Respir Res. 2019 Jul 12;20:152. doi: 10.1186/s12931-019-1105-z (PMC6626346; doi:10.1186/s12931-019-1105-z)
Supplement: Supplementary file 2 — Supplementary Figures. (PDF 1969 kb) [file 12931_2019_1105_MOESM2_ESM.pdf]

Supplementary Figures

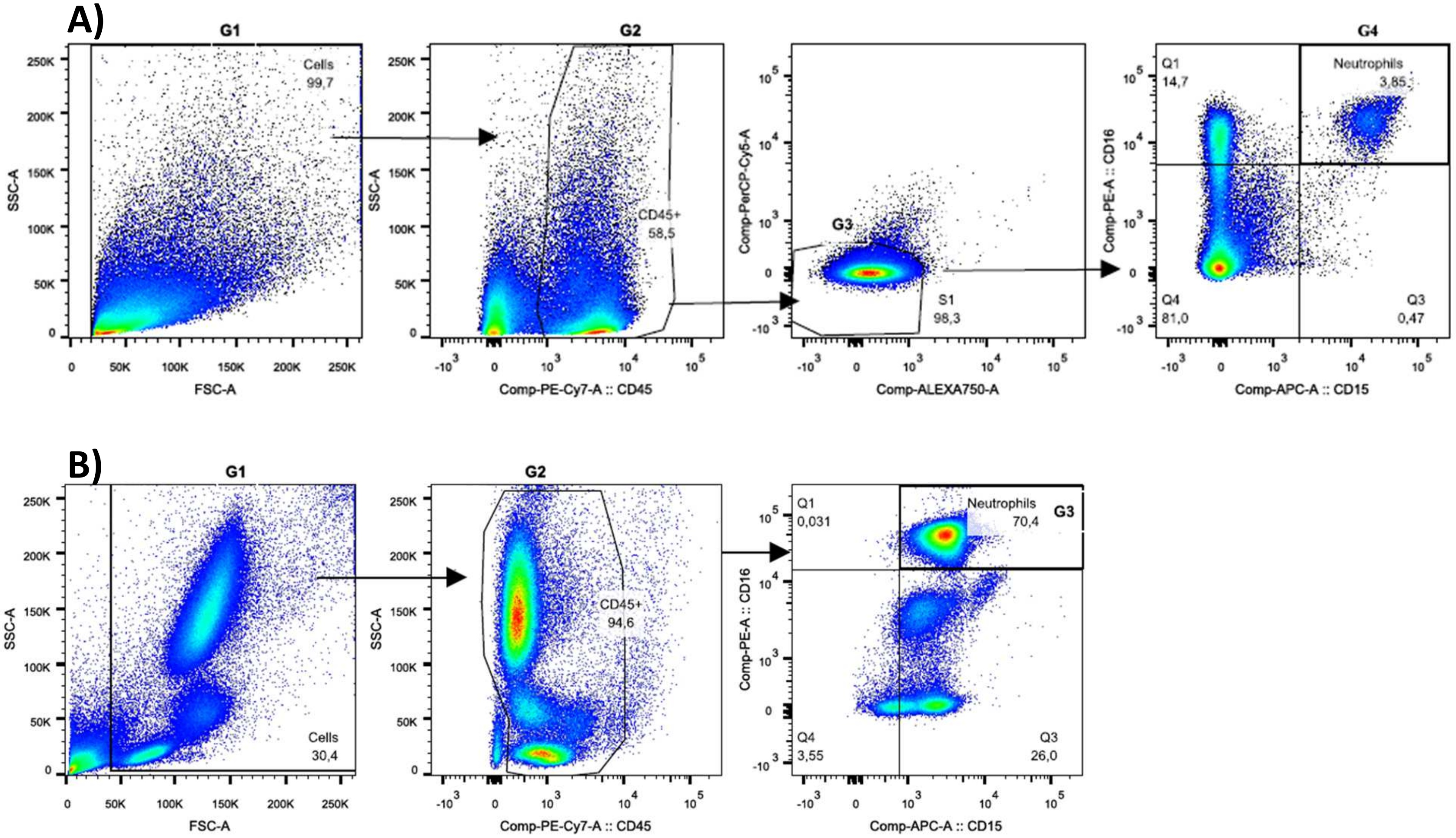
A)
G1
Cells
99,7
SSC-A
FSC-A
G2
CD45+
58,5
Comp-PE-Cy7-A :: CD45
G3
S1
98,3
Comp-PerCP-Cy5-A
Comp-ALEXA750-A
G4
Q1
14,7
Neutrophils
3,85
Q4
81,0
Q3
0,47
Comp-PE-A :: CD16
Comp-APC-A :: CD15
B)
G1
Cells
30,4
SSC-A
FSC-A
G2
CD45+
94,6
Comp-PE-Cy7-A :: CD45
Q1
0,031
Neutrophils
70,4
G3
Q4
3,55
Q3
26,0
Comp-PE-A :: CD16
Comp-APC-A :: CD15

Figure S1

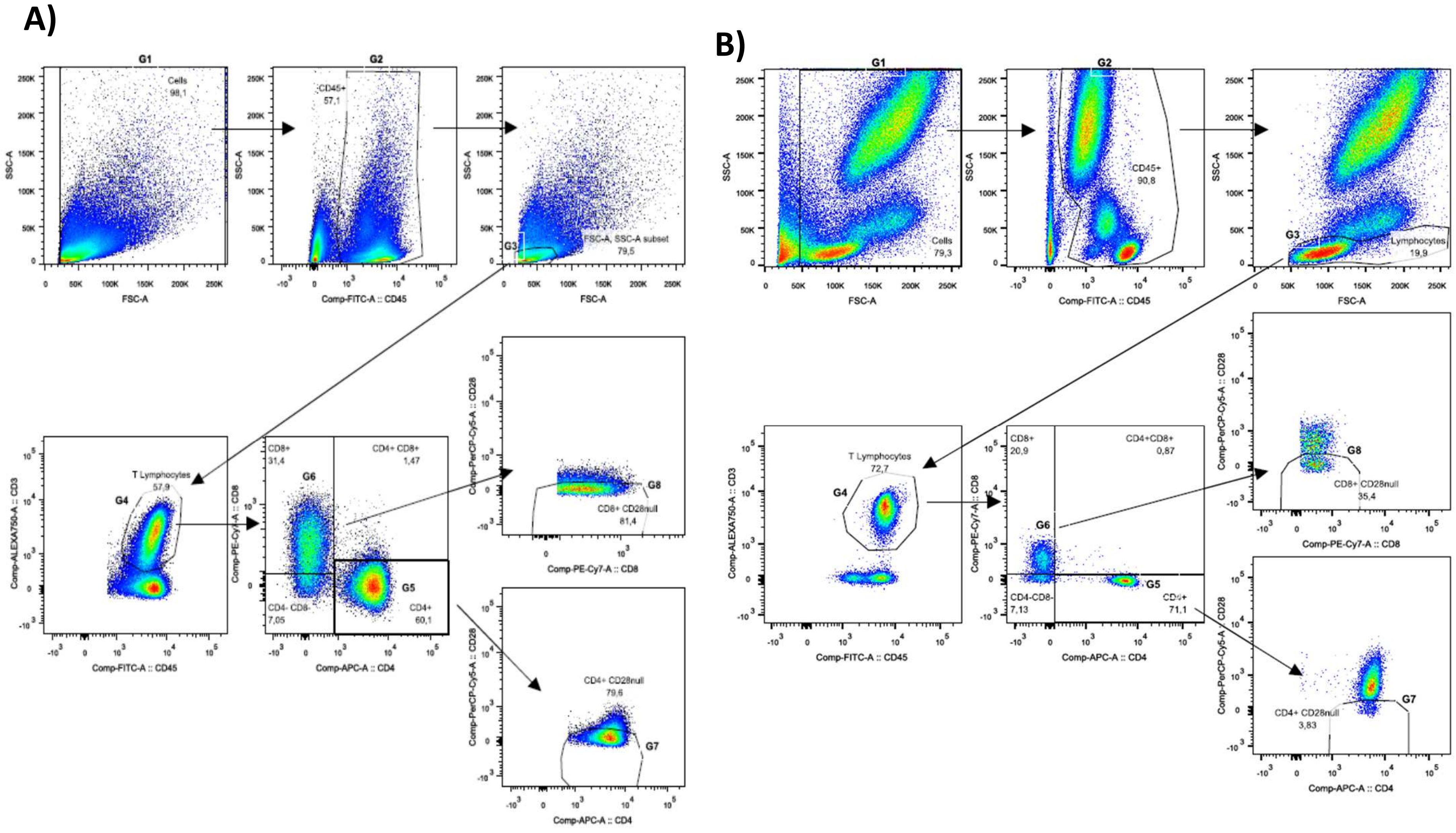
A)
B)
G1
G2
G3
G4
G5
G6
G7
G8
Cells
98,1
CD45+
57,1
FSC-A, SSC-A subset
79,5
T Lymphocytes
57,9
CD8+
31,4
CD4+ CD8+
1,47
CD4- CD8-
7,05
CD4+
60,1
CD8+ CD28null
81,4
CD4+ CD28null
79,6
Cells
79,3
CD45+
90,8
Lymphocytes
19,9
T Lymphocytes
72,7
CD8+
20,9
CD4+CD8+
0,87
CD4-CD8-
7,13
CD4+
71,1
CD8+ CD28null
35,4
CD4+ CD28null
3,83
SSC-A
FSC-A
Comp-FITC-A :: CD45
Comp-ALEXA750-A :: CD3
Comp-PE-Cy7-A :: CD8
Comp-APC-A :: CD4
Comp-PerCP-Cy5-A :: CD28

Figure S2

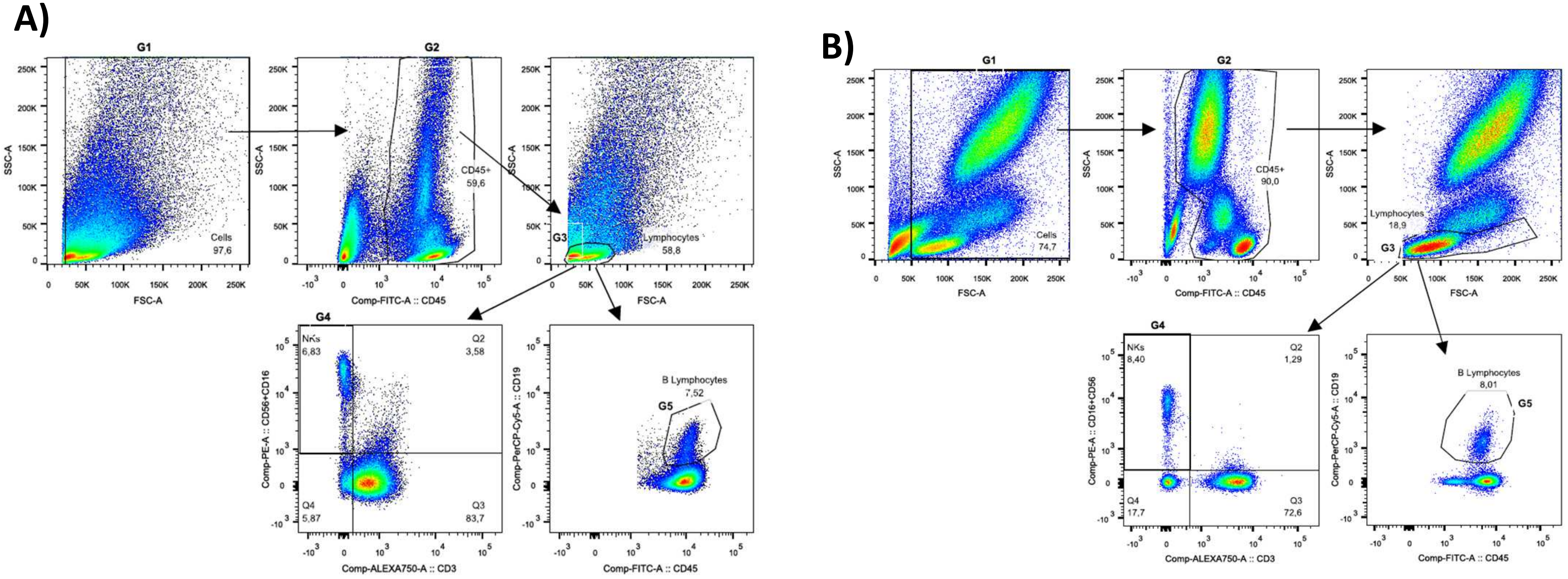
A)
G1
Cells
97,6
SSC-A
FSC-A
G2
CD45+
59,6
Comp-FITC-A :: CD45
G3
Lymphocytes
58,8
G4
NKs
6,83
Q2
3,58
Q4
5,87
Q3
83,7
Comp-PE-A :: CD56+CD16
Comp-ALEXA750-A :: CD3
B Lymphocytes
7,52
G5
Comp-PerCP-Cy5-A :: CD19
B)
G1
Cells
74,7
G2
CD45+
90,0
Lymphocytes
18,9
G3
G4
NKs
8,40
Q2
1,29
Q4
17,7
Q3
72,6
Comp-PE-A :: CD16+CD56
Comp-ALEXA750-A :: CD3
B Lymphocytes
8,01
G5
Comp-PerCP-Cy5-A :: CD19
Comp-FITC-A :: CD45

Figure S3

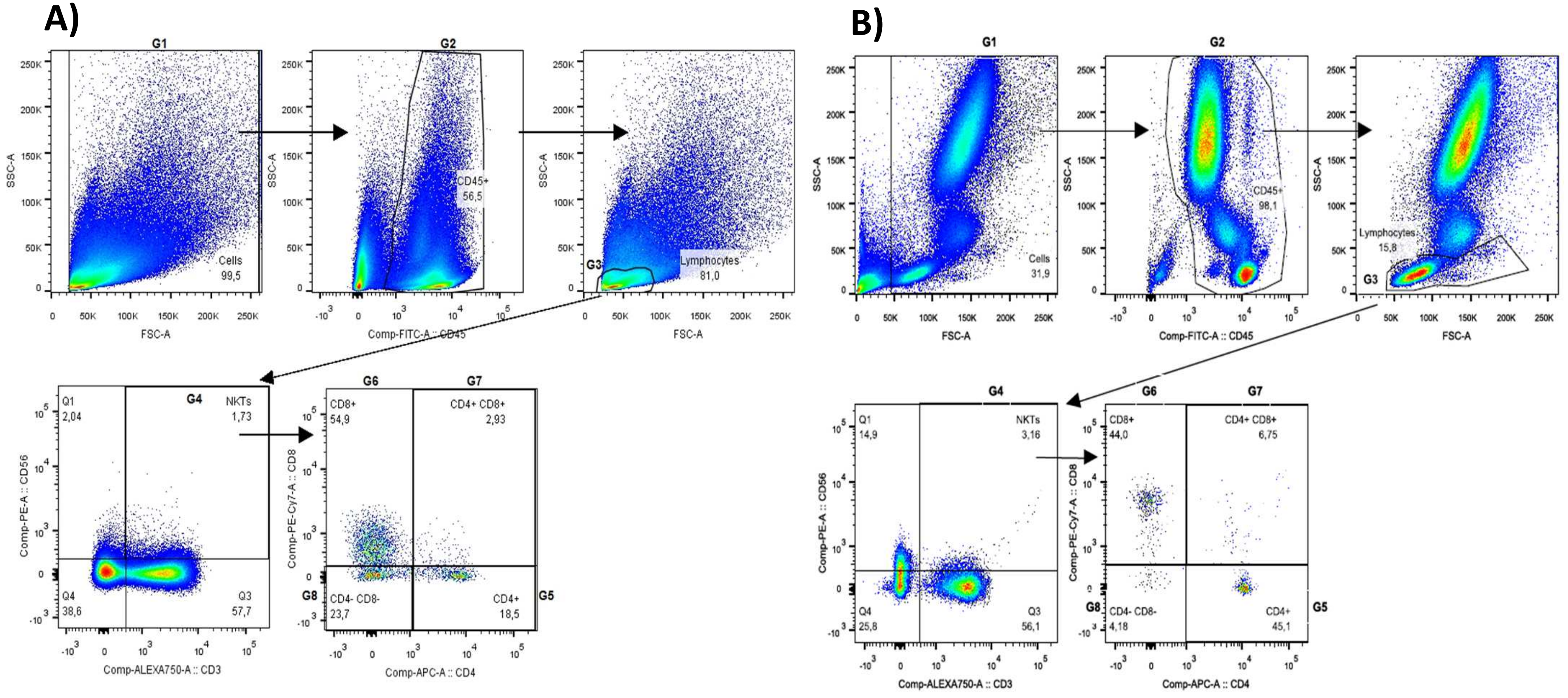
A)
B)
G1
G2
G3
G4
G5
G6
G7
G8
SSC-A
FSC-A
Comp-FITC-A :: CD45
Cells
99,5
CD45+
56,5
Lymphocytes
81,0
Comp-PE-A :: CD56
Comp-ALEXA750-A :: CD3
Q1
2,04
NKTs
1,73
Q4
38,6
Q3
57,7
Comp-PE-Cy7-A :: CD8
Comp-APC-A :: CD4
CD8+
54,9
CD4+ CD8+
2,93
CD4- CD8-
23,7
CD4+
18,5
Cells
31,9
CD45+
98,1
Lymphocytes
15,8
Q1
14,9
NKTs
3,16
Q4
25,8
Q3
56,1
CD8+
44,0
CD4+ CD8+
6,75
CD4- CD8-
4,18
CD4+
45,1

Figure S4

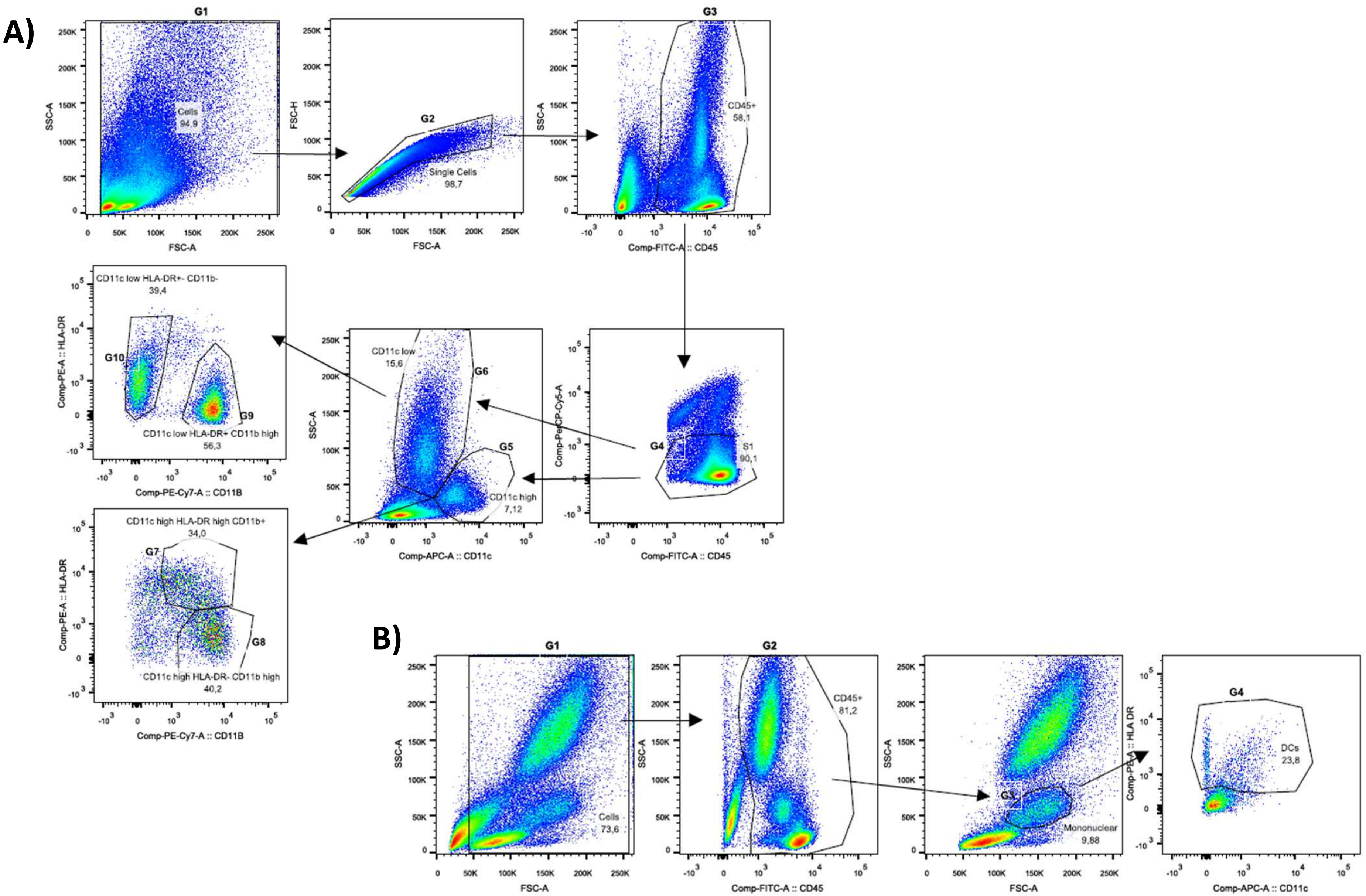
A)
G1
Cells
94,9
SSC-A
FSC-A
G2
Single Cells
98,7
FSC-H
FSC-A
G3
CD45+
58,1
SSC-A
Comp-FITC-A :: CD45
G4
S1
90,1
Comp-PerCP-Cy5-A
Comp-FITC-A :: CD45
CD11c low
15,6
G6
G5
CD11c high
7,12
SSC-A
Comp-APC-A :: CD11c
CD11c low HLA-DR+- CD11b-
39,4
G10
G9
CD11c low HLA-DR+ CD11b high
56,3
Comp-PE-A :: HLA-DR
Comp-PE-Cy7-A :: CD11B
CD11c high HLA-DR high CD11b+
34,0
G7
G8
CD11c high HLA-DR- CD11b high
40,2
Comp-PE-A :: HLA-DR
Comp-PE-Cy7-A :: CD11B
B)
G1
Cells
73,6
SSC-A
FSC-A
G2
CD45+
81,2
SSC-A
Comp-FITC-A :: CD45
G3
Mononuclear
9,88
SSC-A
FSC-A
G4
DCs
23,8
Comp-PE-A :: HLA DR
Comp-APC-A :: CD11c

Figure S5

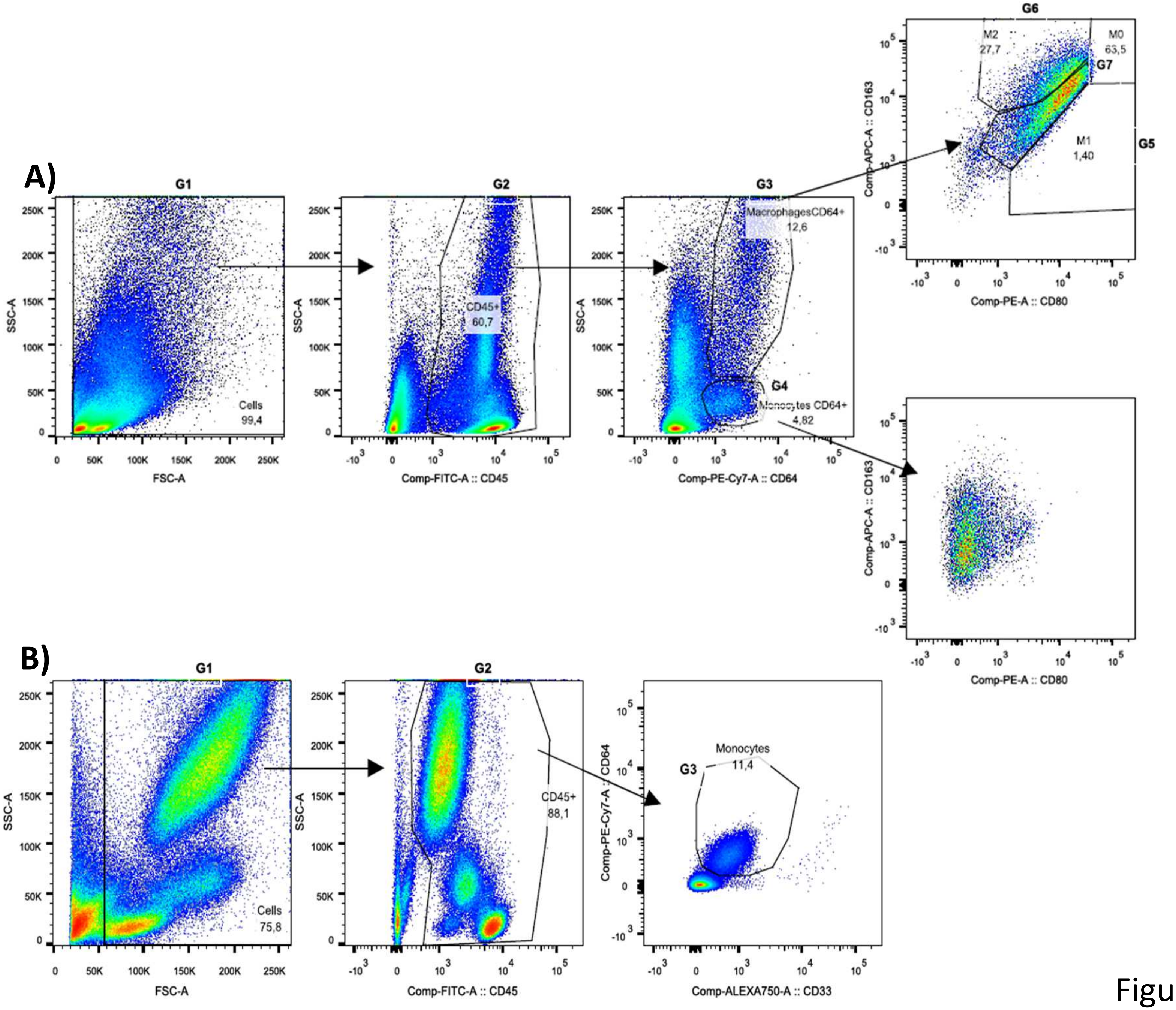

A)
G1
SSC-A
FSC-A
Cells
99,4
G2
CD45+
60,7
Comp-FITC-A :: CD45
G3
MacrophagesCD64+
12,6
G4
Monocytes CD64+
4,82
Comp-PE-Cy7-A :: CD64
G6
M2
27,7
M0
63,5
G7
M1
1,40
G5
Comp-APC-A :: CD163
Comp-PE-A :: CD80
B)
G1
SSC-A
FSC-A
Cells
75,8
G2
CD45+
88,1
Comp-FITC-A :: CD45
Monocytes
11,4
G3
Comp-PE-Cy7-A :: CD64
Comp-ALEXA750-A :: CD33

Figure S6

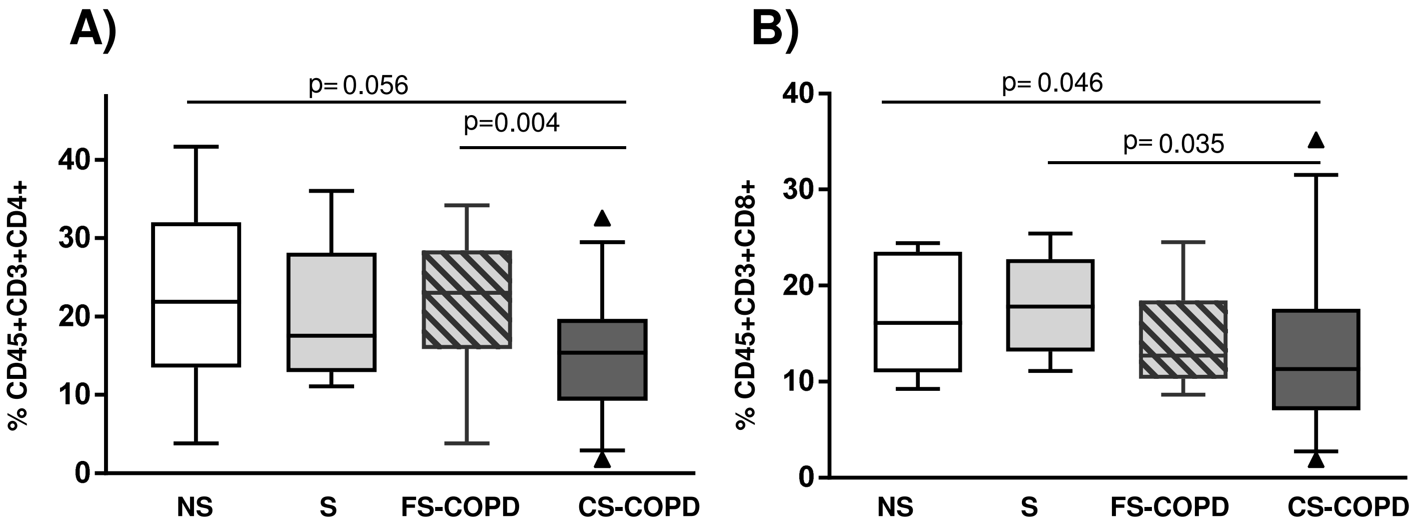
A)
p= 0.056
p=0.004
% CD45+CD3+CD4+
40
30
20
10
0
NS
S
FS-COPD
CS-COPD
B)
p= 0.046
p= 0.035
% CD45+CD3+CD8+
40
30
20
10
0
NS
S
FS-COPD
CS-COPD

Figure S7

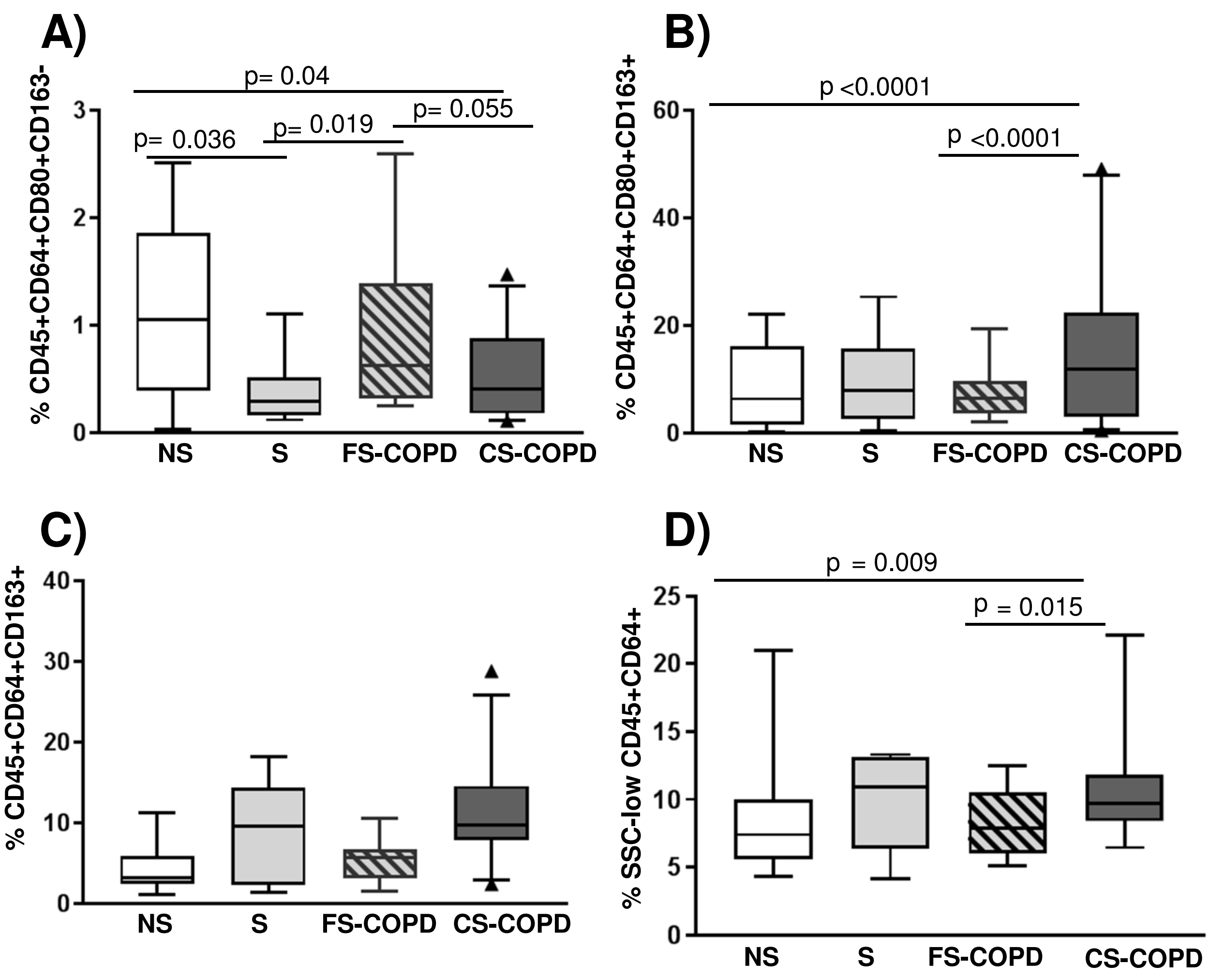
A)
p= 0.04
p= 0.036
p= 0.019
p= 0.055
% CD45+CD64+CD80+CD163-
3
2
1
0
NS
S
FS-COPD
CS-COPD
B)
p <0.0001
p <0.0001
% CD45+CD64+CD80+CD163+
60
40
20
0
NS
S
FS-COPD
CS-COPD
C)
% CD45+CD64+CD163+
40
30
20
10
0
NS
S
FS-COPD
CS-COPD
D)
p = 0.009
p = 0.015
% SSC-low CD45+CD64+
25
20
15
10
5
0
NS
S
FS-COPD
CS-COPD

Figure S8

MODULE	MΦ	MΦ CD80+	MΦ CD163+	MΦ CD80+CD163+	MAIN GENE ONTOLOGIES
Tan	0.00282 (0.0303)	0.02831 (0.4627)	0.00288 (0.0628)	0.00478 (0.2075)	- Phospholipid catabolism<br>- Iron ion transport
Greenyellow	0.00230 (0.1169)	-0.04444 (0.2990)	0.00178 (0.3087)	0.00853 (0.0403)	- Collagen metabolism<br>- Extracellular matrix organization
Yellow	-0.00112 (0.4047)	-0.00724 (0.8531)	-0.00098 (0.5358)	-0.00326 (0.3973)	- Cilium organization
Royalblue	-0.00162 (0.2068)	-0.01804 (0.6296)	-0.00184 (0.2257)	-0.00232 (0.5308)	- T cell activation
Lightcyan	0.00376 (0.0074)	-0.00896 (0.8321)	0.00498 (0.0024)	0.00168 (0.6874)	- mRNA processing
Blue	0.00264 (0.0757)	0.01240 (0.7765)	0.00400 (0.0207)	-0.00104 (0.8092)	- Regulation of translation<br>- ER to Golgi ves-med transport
Lightcyan	-0.00232 (0.1039)	0.01705 (0.6840)	-0.00308 (0.0663)	-0.00262 (0.5274)	- Proteasomal protein catabolism
Salmon	-0.00183 (0.1936)	0.03392 (0.4085)	-0.00216 (0.1936)	-0.00321 (0.4298)	- Negative Regulation of translation

Figure S9
